# Correction: The Genomic Aftermath of Hybridization in the Opportunistic Pathogen *Candida metapsilosis*

**DOI:** 10.1371/journal.pgen.1006202

**Published:** 2016-07-14

**Authors:** Leszek P. Pryszcz, Tibor Németh, Ester Saus, Ewa Ksiezopolska, Eva Hegedűsová, Jozef Nosek, Kenneth H. Wolfe, Attila Gacser, Toni Gabaldón

There is an error in the Data Availability Statement. Specifically, the accession number PRJNA238968 is obsolete, and the accession number PRJEB1698 should be used to access the sequencing data deposited in the EBI-ENA database (http://www.ebi.ac.uk/ena/data/view/PRJEB1698).
