# Supplementary figures and images for: The Genomic Aftermath of Hybridization in the Opportunistic Pathogen Candida metapsilosis
Source: PLoS Genet. 2015 Oct 30;11(10):e1005626. doi: 10.1371/journal.pgen.1005626 (PMC4627764; doi:10.1371/journal.pgen.1005626)

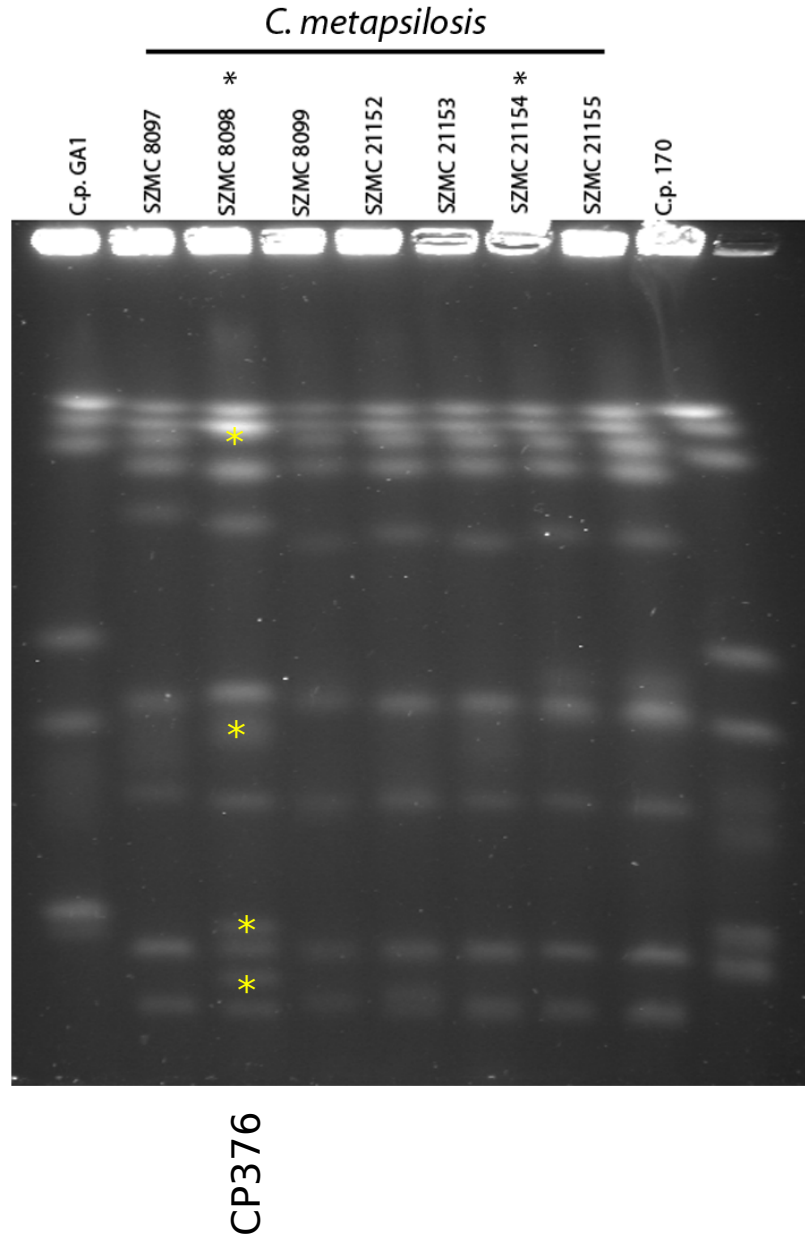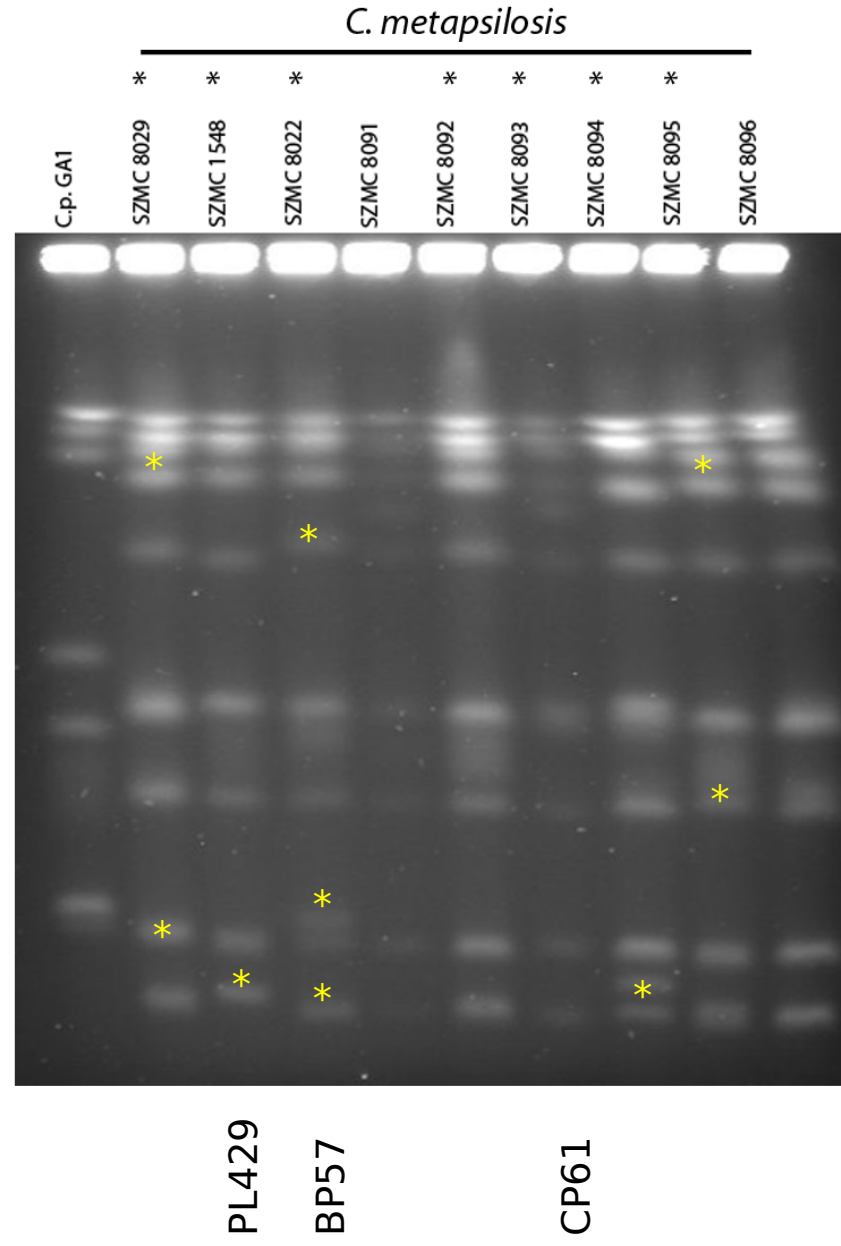

Supplement: S1 Fig — The chromosomes of several C. metapsilosis strains were subjected to Pulse Field Gel Electrophoresis (PFGE). Two C. parapsilosis strains were included as reference. Chromosomes with potential rearrangements are denoted with yellow asterisks. Sequenced strains are marked with black asterisks. (PDF) [file pgen.1005626.s001.pdf]

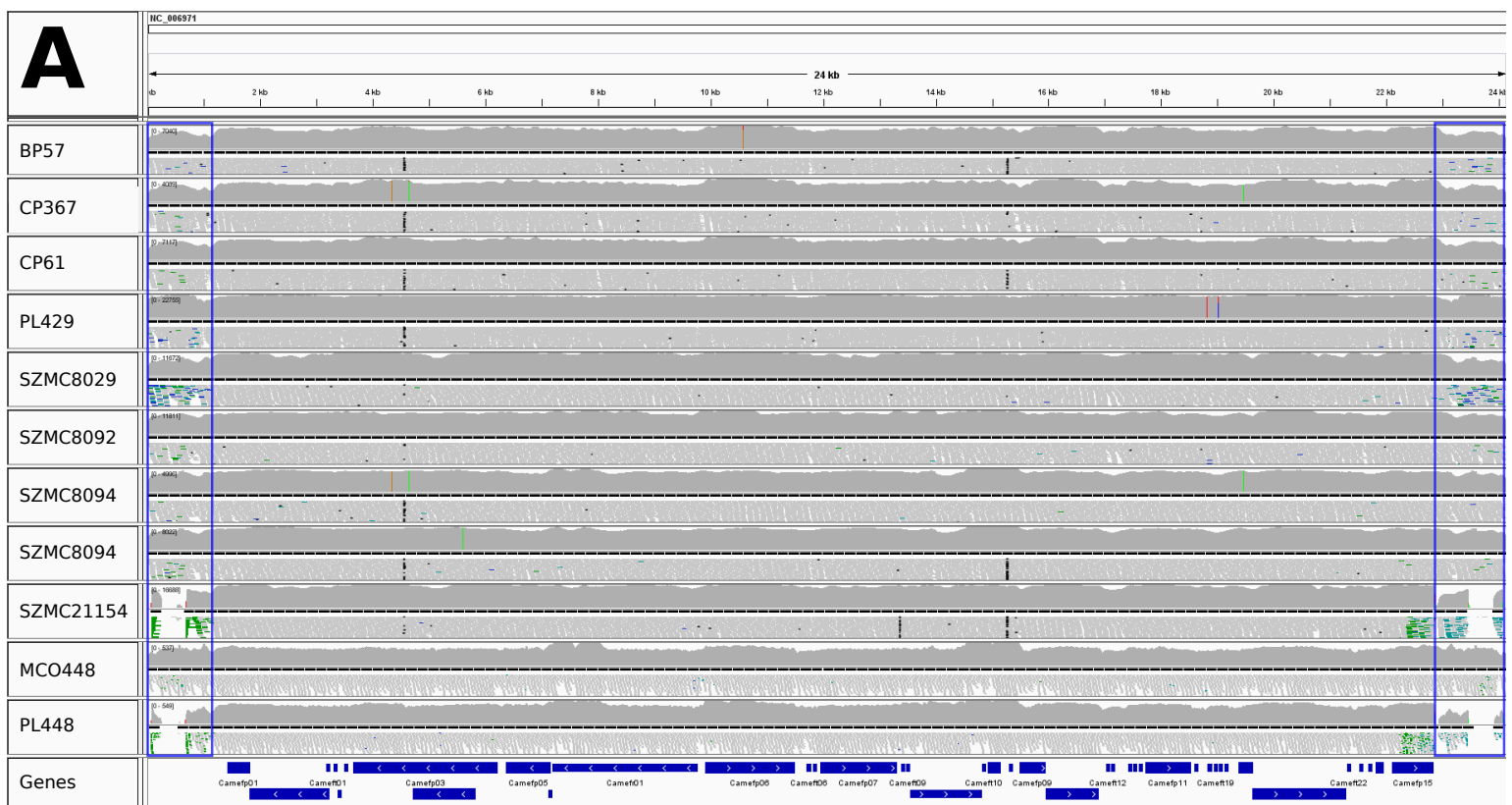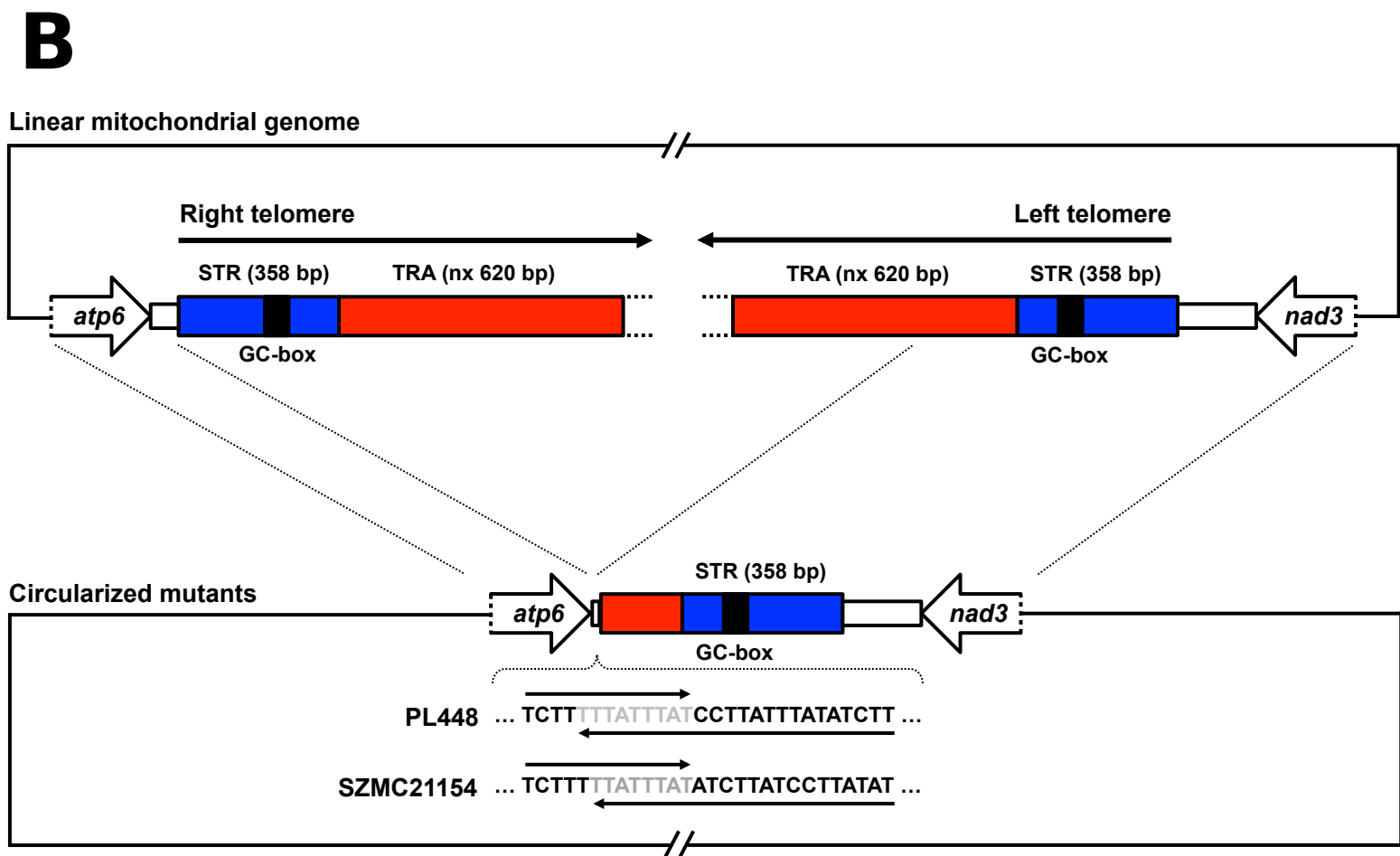

STR – subterminal repeat (358 bp)  
TRA – tandem repeat array (nx 620 bp)

Supplement: S2 Fig — A. Genomic reads from all strains were aligned on the linear mitochondrial chromosome reference of Candida metasilosis MCO448 (NC_006971). Telomeres (marked with blue squares) are expected only in linear chromosomes, thus strains with circular mitochondrial chromosome (PL448 and SZMC21154) miss parts of telomeres. In addition, read pairs aligned on the ends of the reference present discordant pairing (marked in green) in strains with circular mitochondrial chromosome. B. The most of C. metapsilosis strains examined in this study contain a linear mitochondrial genome with the length of about 23 kbp (Kosa et al., 2006). The genes atp6 and nad3 are located in the right and left subterminal region, respectively, and the telomeres on both ends of linear DNA molecules consist of a subterminal repeat (STR, 358 bp, shown as blue rectangle) and a tandem repeat array (TRA, nx 620 bp, red rectangle). The STR contains a sequence cluster rich in guanine and cytosine residues (GC-box, black rectangle), which is presumably involved in the mitochondrial telomere maintenance via homologous recombination (Nosek et al., 2005; Gerhold et al. 2014). In contrast, the strains PL448 and SZMC21154 contain circular mitochondrial genomes that lack the right telomere and most of the tandem repeat array of the left telomere. The sequence analysis indicates that these genomes represent circularized mutants resulting form end-to-end fusions of originally linear DNA molecules. Since the sites where the fusion events occurred are different, we assume that these mutants emerged independently. (PDF) [file pgen.1005626.s002.pdf]

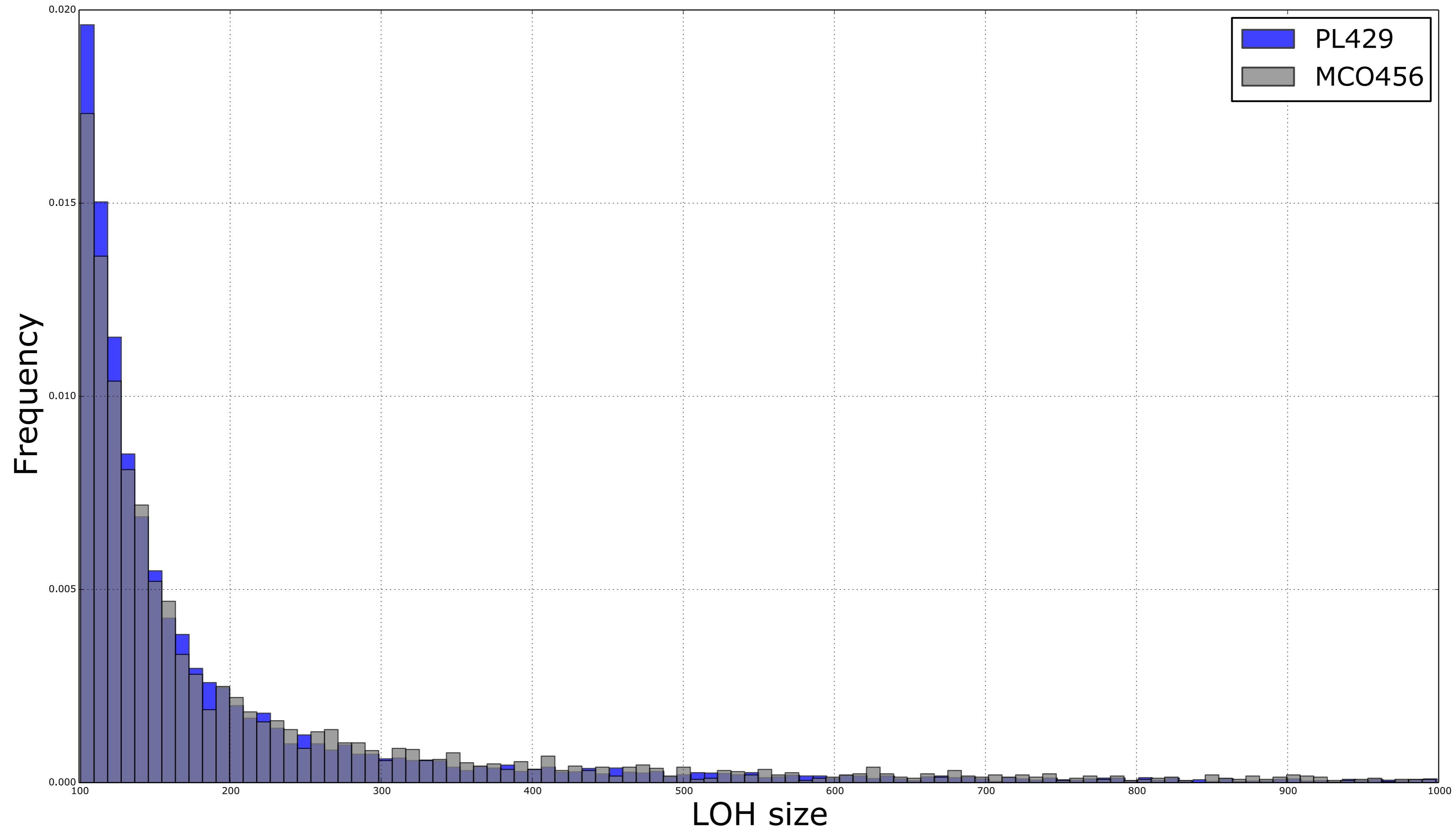

Supplement: S3 Fig — Histogram of LOH block sizes for C. metapsilosis PL429 (blue) and C. orthopsilosis MCO 456 (grey). Only LOH shorter than 10 kb are shown. Note, Y axis is log-scaled. (PDF) [file pgen.1005626.s003.pdf]

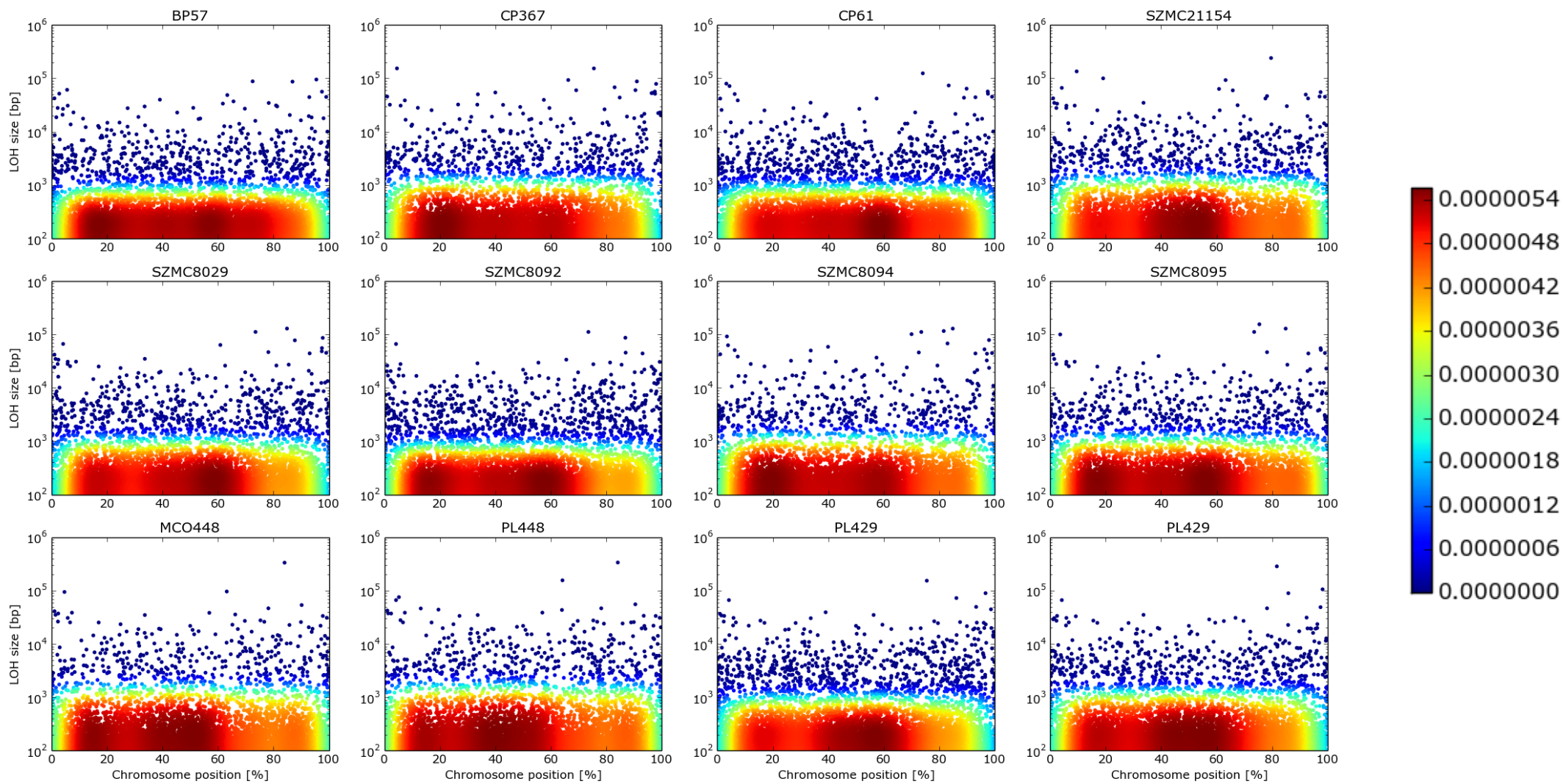

Supplement: S4 Fig — Scatter plot representing the size of loss of heterozygosity (LOH) regions and their position on the chromosome given as percentage of chromosome length. Colors represent dots density. Note, two replicas for PL429 (pe300 and pe600) are given in two separate panels. (PDF) [file pgen.1005626.s004.pdf]

## A. PCR

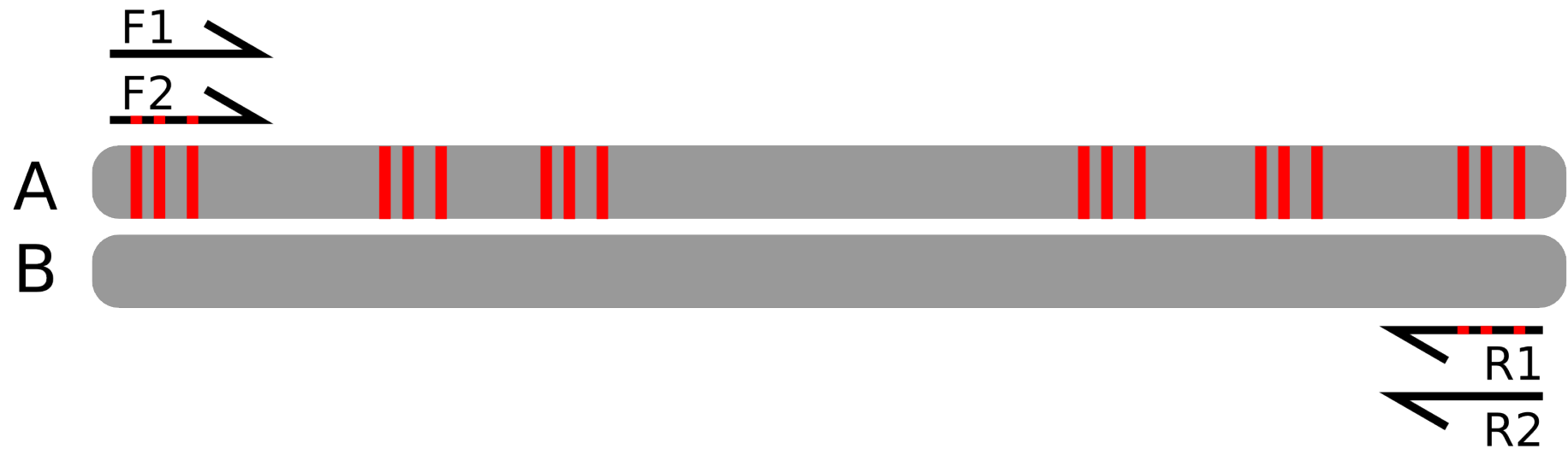

## B. Sanger sequencing

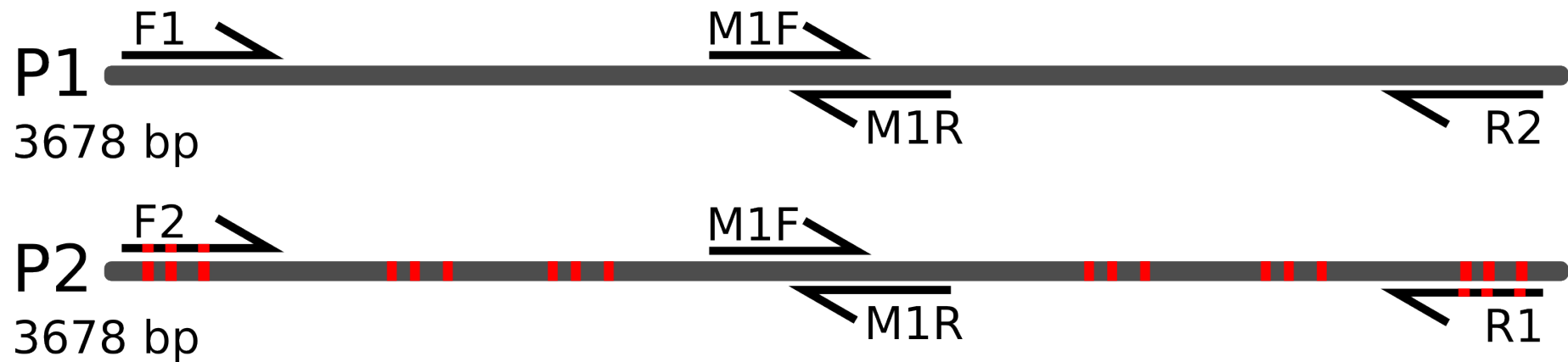

Supplement: S5 Fig — Details for the independent amplification of an heterozygous region (3678 bp) from two homologous chromosomes. A. Sets of primers (F1, R1 and F2, R2) targeting known heterozygous sequences that flank the region of interest were designated. Forward (F1, F2) and reverse (R1, R2) primers differ by three SNP, so all primers align uniquely to a specific homologous chromosome. Four PCR reactions were performed for each strain. For these only two PCR reactions gave a product (positive controls): P1 obtained from combination of F1 and R2; and P2 from F2 and R1. The other two combinations of primers (F1R1 and F2R2) did not work as the primers anneal on homologous chromosome and not on the same chromosome (negative controls). B. Obtained PCR products are too larger to be sequence by single Sanger reaction. We thus have designed two additional primers (M1F, M1R) that target homozygous region in the middle of PCR products, so they can be used with both PCR products. For each PCR product (P1, P2) we performed four Sanger sequencing reactions: For F1R2 amplification product (P1): ∘pos4_F1 primer amplification (F1),∘pos4_R2 primer amplification (R2),∘pos4_M1F (M1F_12),∘pos4_M1R (M1R_12) For F2R1 amplification product: ∘pos4_F2 primer amplification (F2),∘pos4_R1 primer amplification (R1),∘pos4_M1F (M1F_21),∘pos4_M1R (M1R_21) (PDF) [file pgen.1005626.s005.pdf]

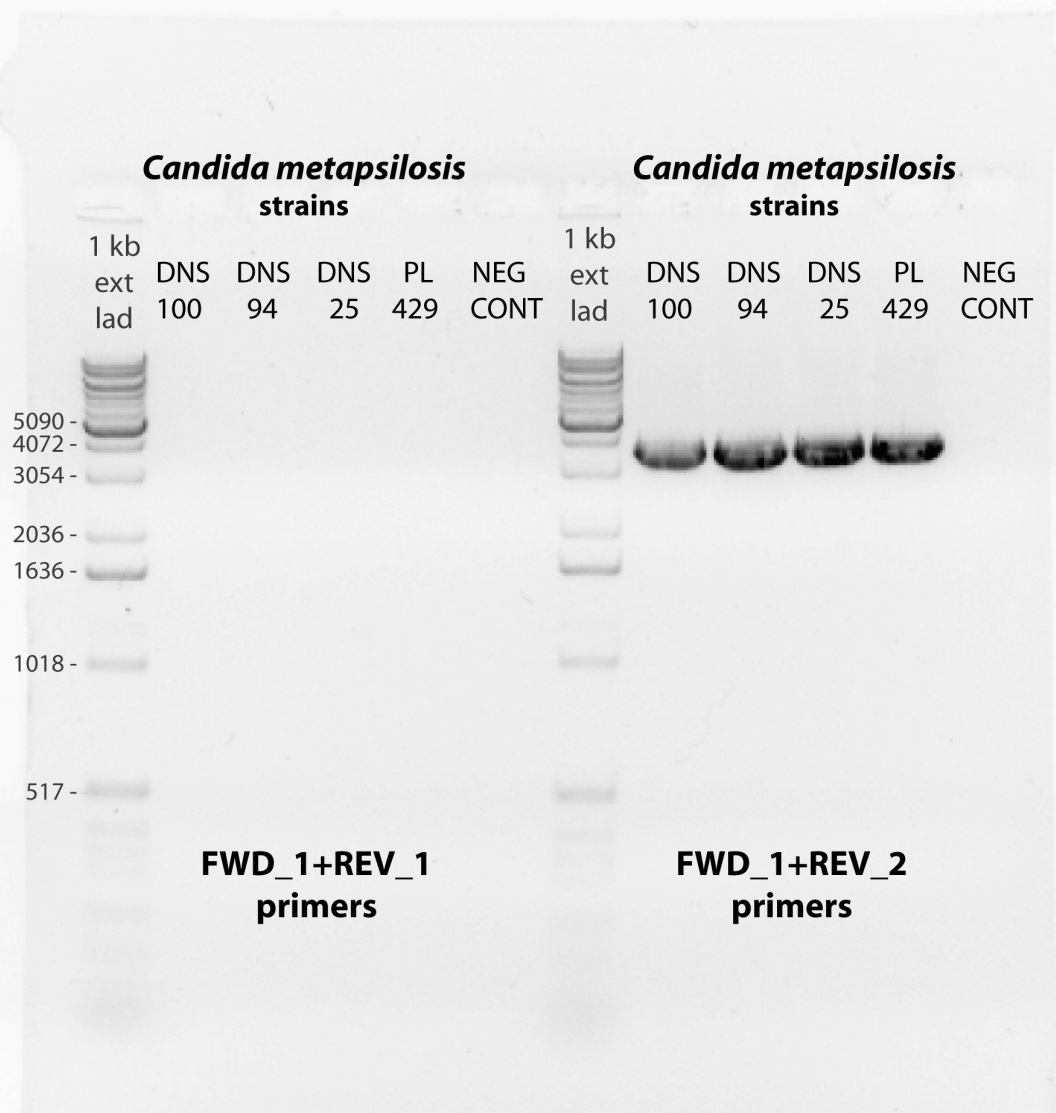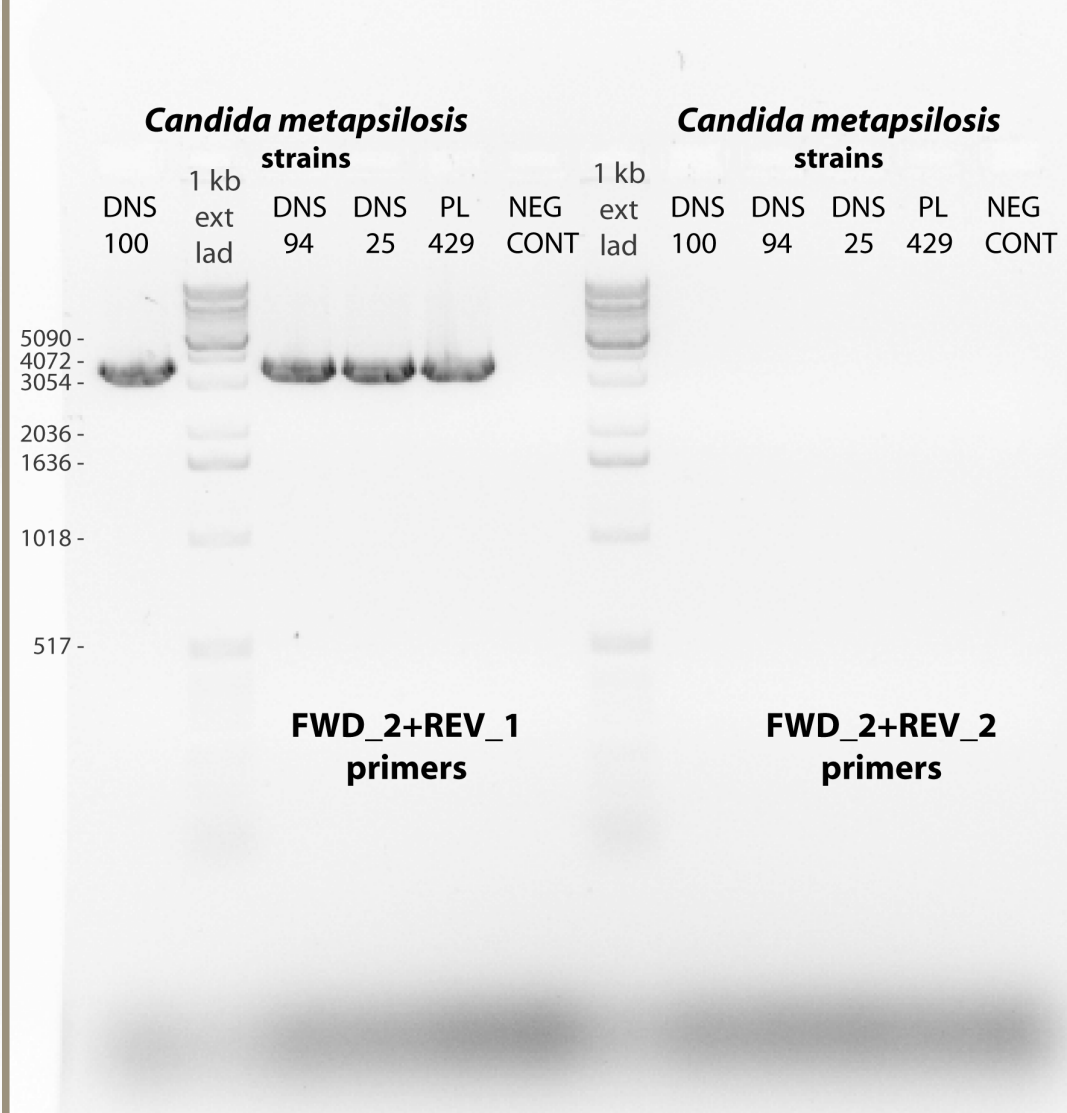

Supplement: S6 Fig — Two forward primers and two reverse primers, with four and three different bases among them, respectively, corresponding to allelic differences in each parental sequence/genotype were designed. Thus, four touchdown PCR reactions (FWD_1+REV_1, FWD_1+REV_2, FWD_2+REV_1 and FWD_2+REV_2) were carried out. Specific PCR products were only obtained when combining the primer sets FWD_1+REV_2, and FWD_2+REV_1 (amplicon size of 3678 bp), while no band was seen when combining FWD_1+REV_1 or FWD_2+REV_2 primers. (PDF) [file pgen.1005626.s006.pdf]

**A**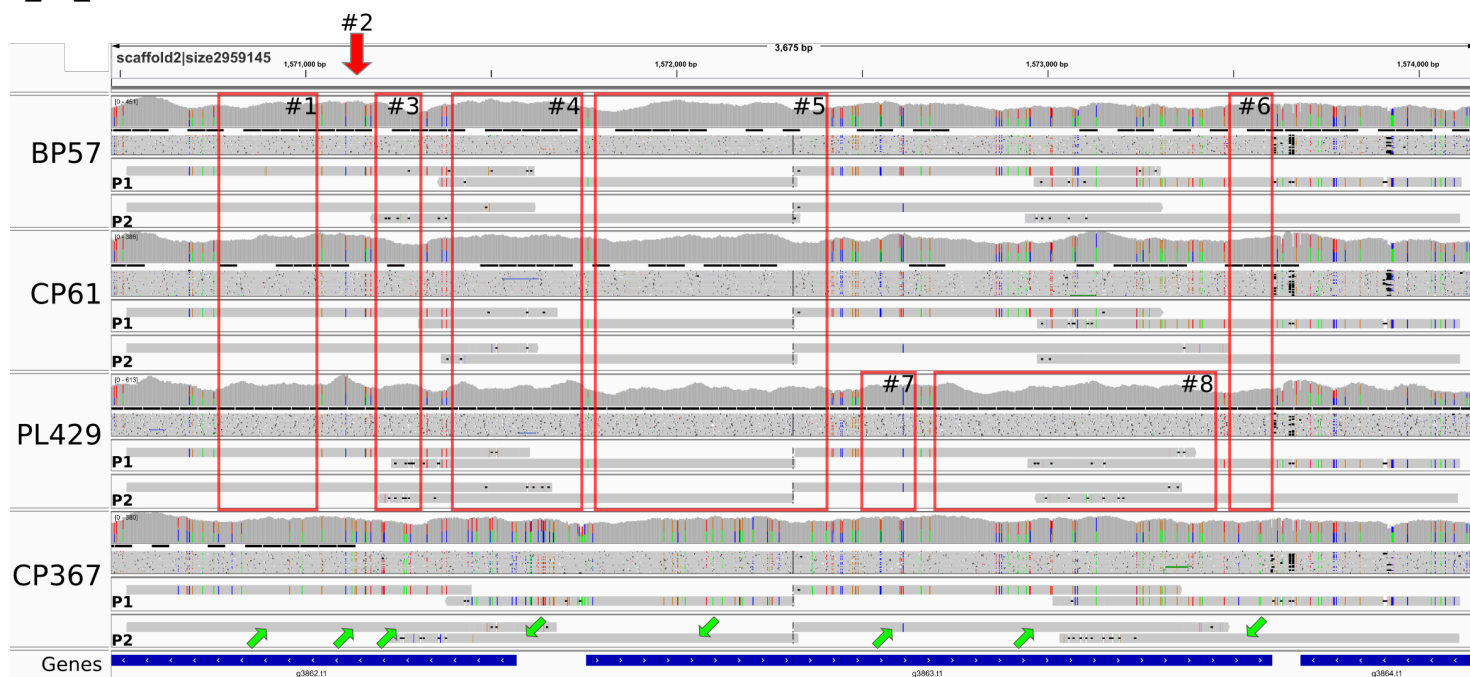**B**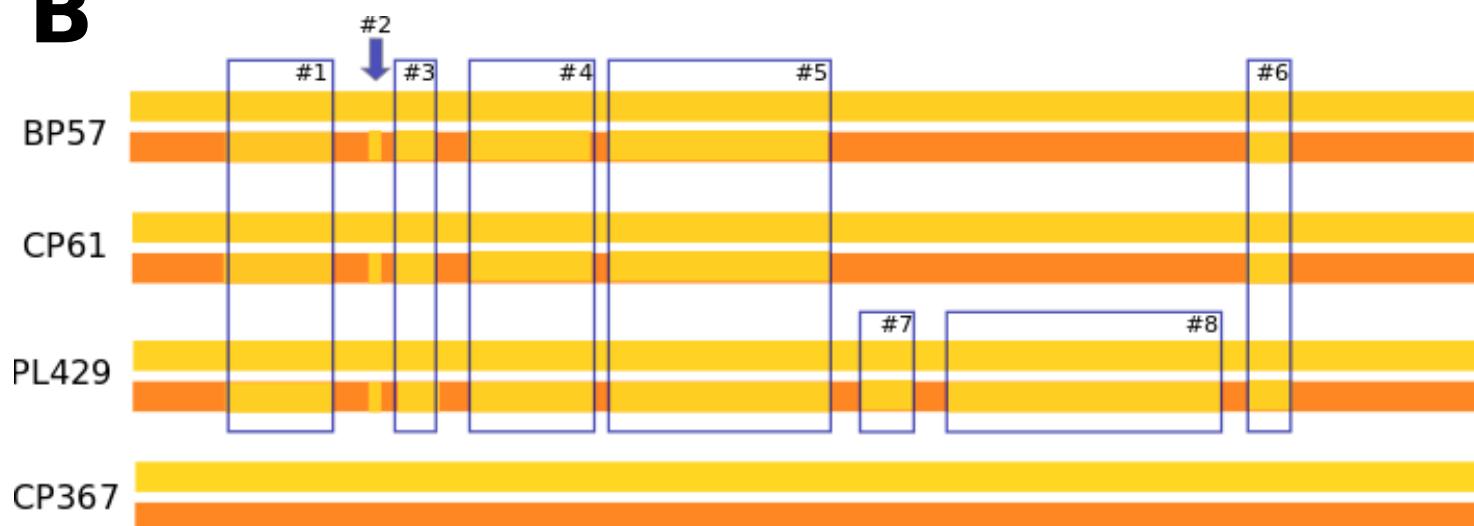

Supplement: S7 Fig — In order to test whether LOH donor is randomly or preferentially selected, we have analysed in detail 3.6 kb genomic region (scaffold2|size2959145:1,570,477–1,574,155) harboring eight LOH in four C. metapsilosis strains: BP57 (DNS25), CP61 (DNS94), CP367 (DNS100) and PL429. A) Following features are given for each strain: genomic read density and alignments, followed by aligned Sanger sequences from PCR product 1 (P1) and product 2 (P2). LOH events are marked by red rectangles or red arrow. The recombination donor is marked by green arrow. All recombinations in the analysed region originated from PCR product 2 (P2). B) Schematic representation of LOH events. Six LOH events (#1-#6) are common to three strains (BP57, CP61, PL429), while the remaining two (#7 and #8) are present only in PL429. (PDF) [file pgen.1005626.s007.pdf]

## A. PL429

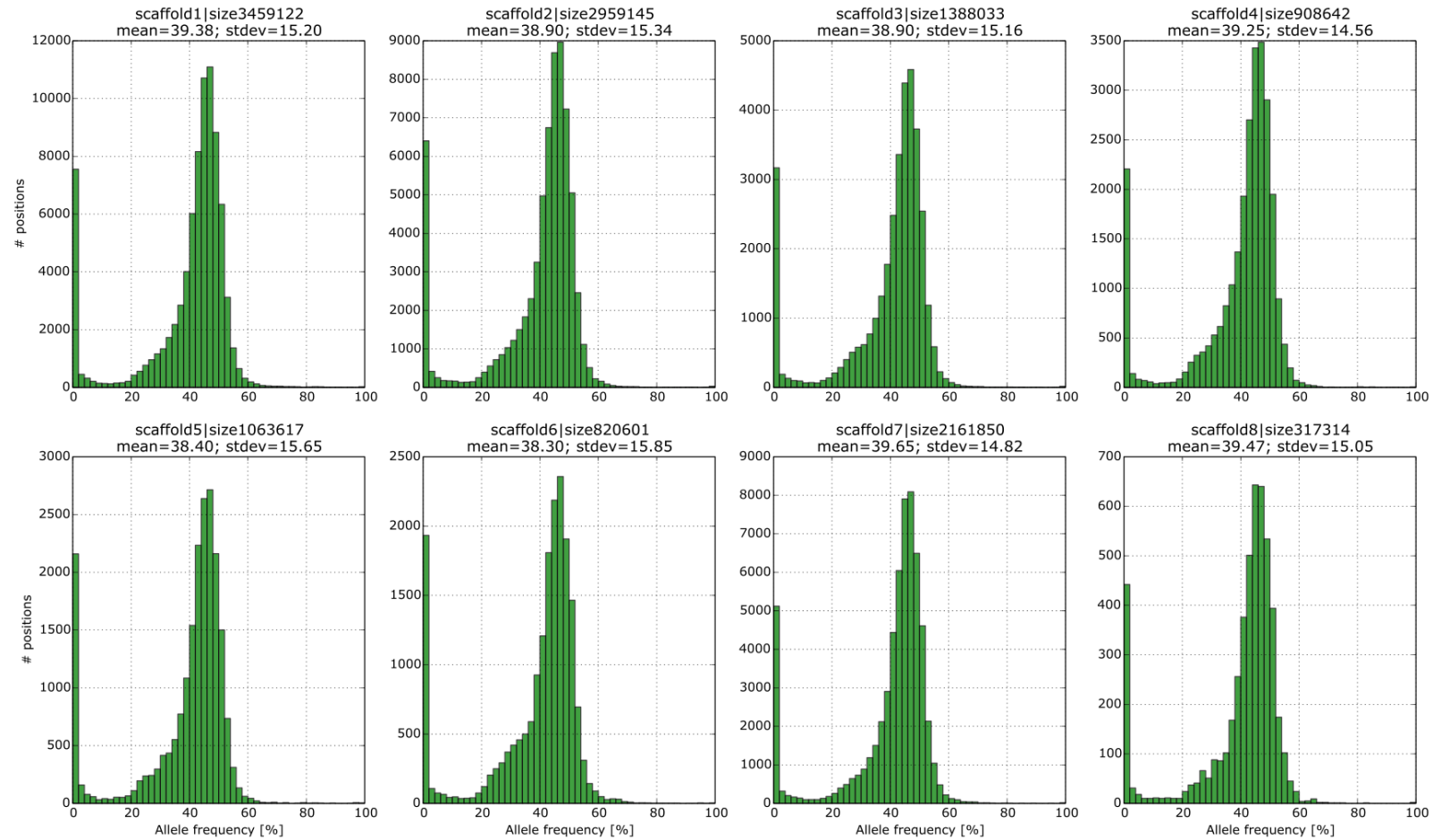

## B. PL448

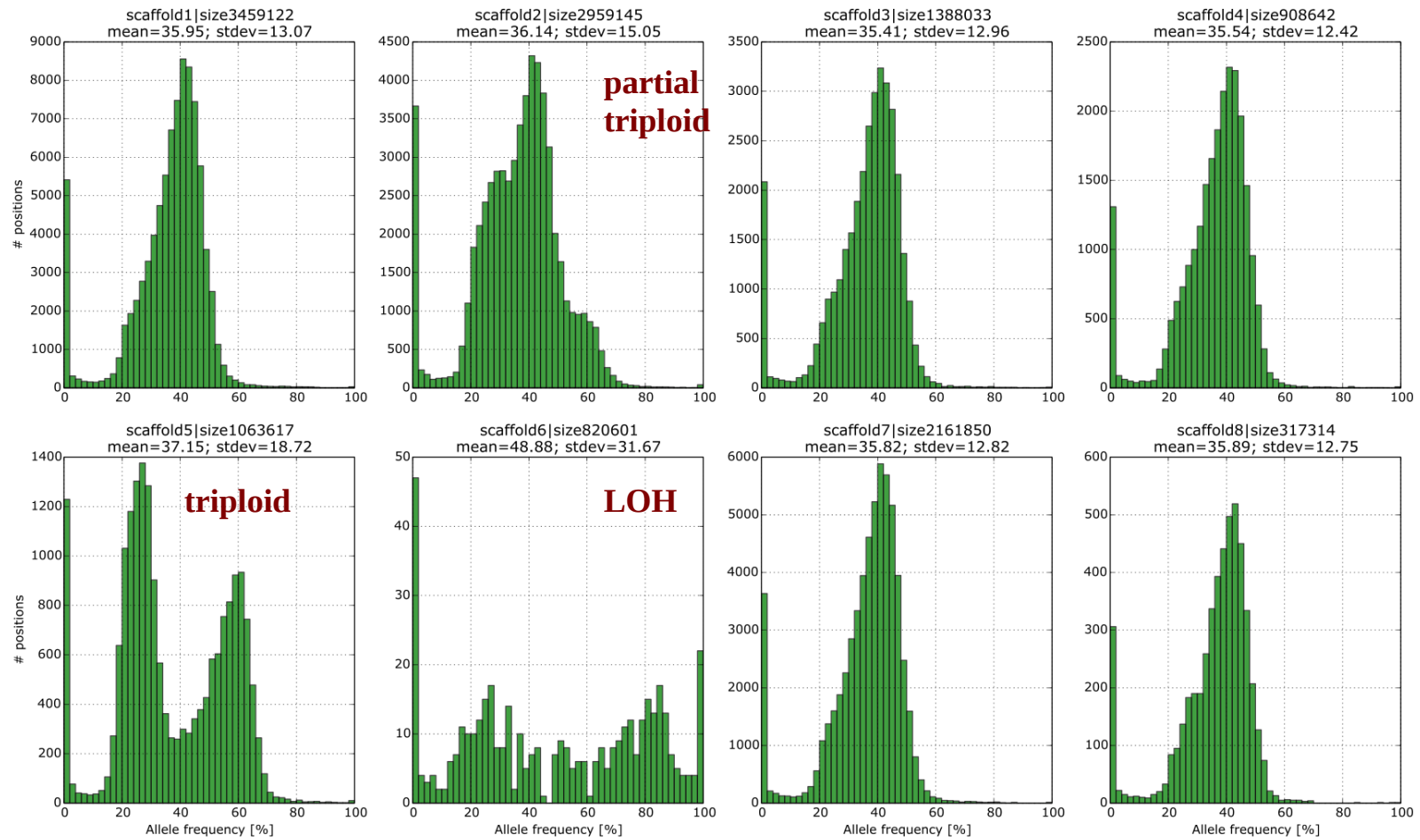

## C. SZMC21154

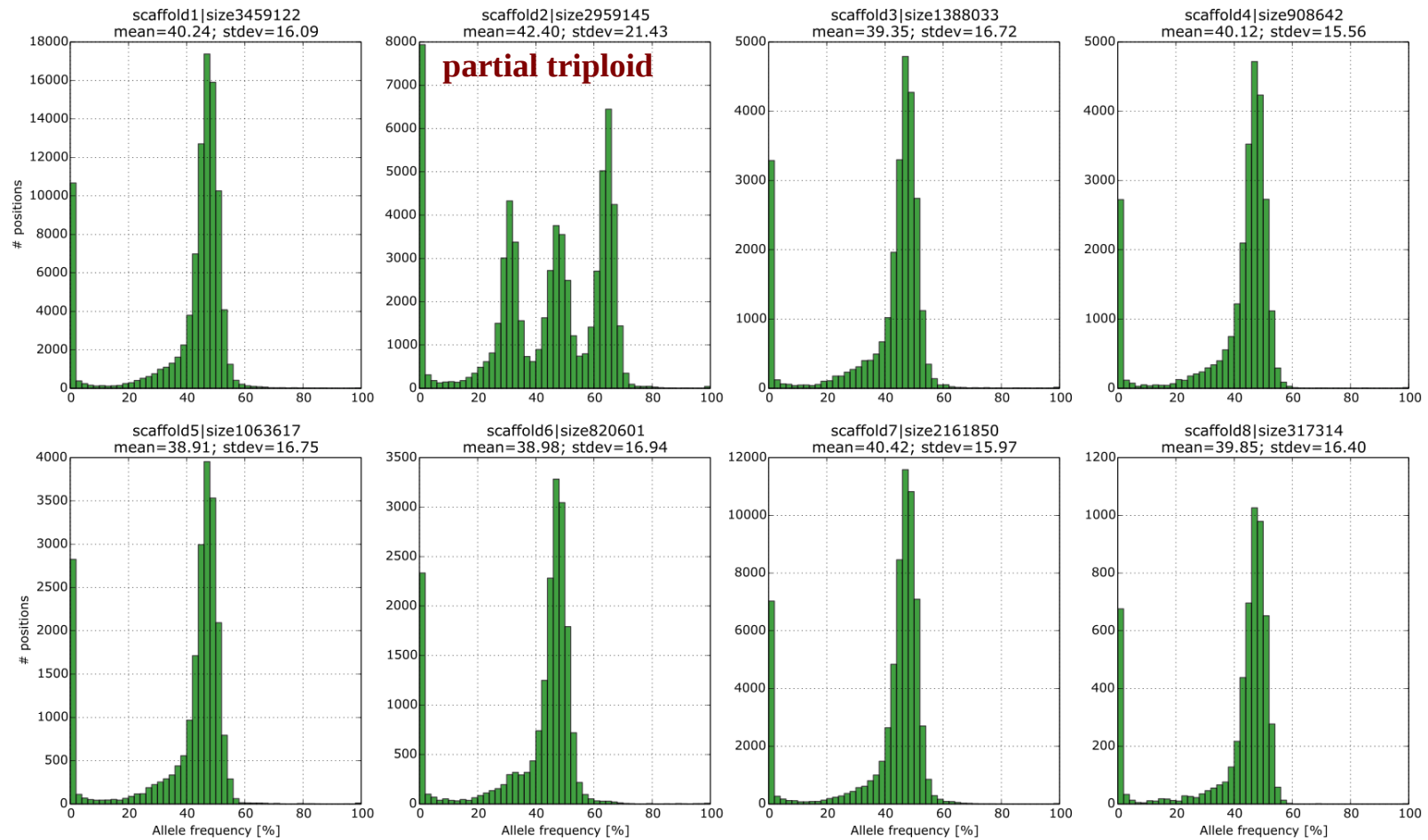

Supplement: S8 Fig — Distributions of frequencies of read counts at biallelic SNPs in C. metapsilosis strains. (A) We observed single peak close to 50% for all chromosomes of C. metapsilosis PL429, indicating it is diploid. The remaining strains are also diploid, with exceptions in some chromosomes: (B) PL448 scaffold5 is present in three copies (two peaks at 33% and 66%), scaffold6 underwent LOH (very few biallelic SNPs and no peaks) and end of scaffold2 (2–2.9Mb) is present in three copies (two peaks at 33% and 66% visible beside main peak at 50%); and (C) SZMC21154 scaffold2 from 0 to 2Mb is present in three copies while the remaining 0.9 Mb in two copies (two peaks at 33% and 66% beside main peak at 50%). (PDF) [file pgen.1005626.s008.pdf]

# A

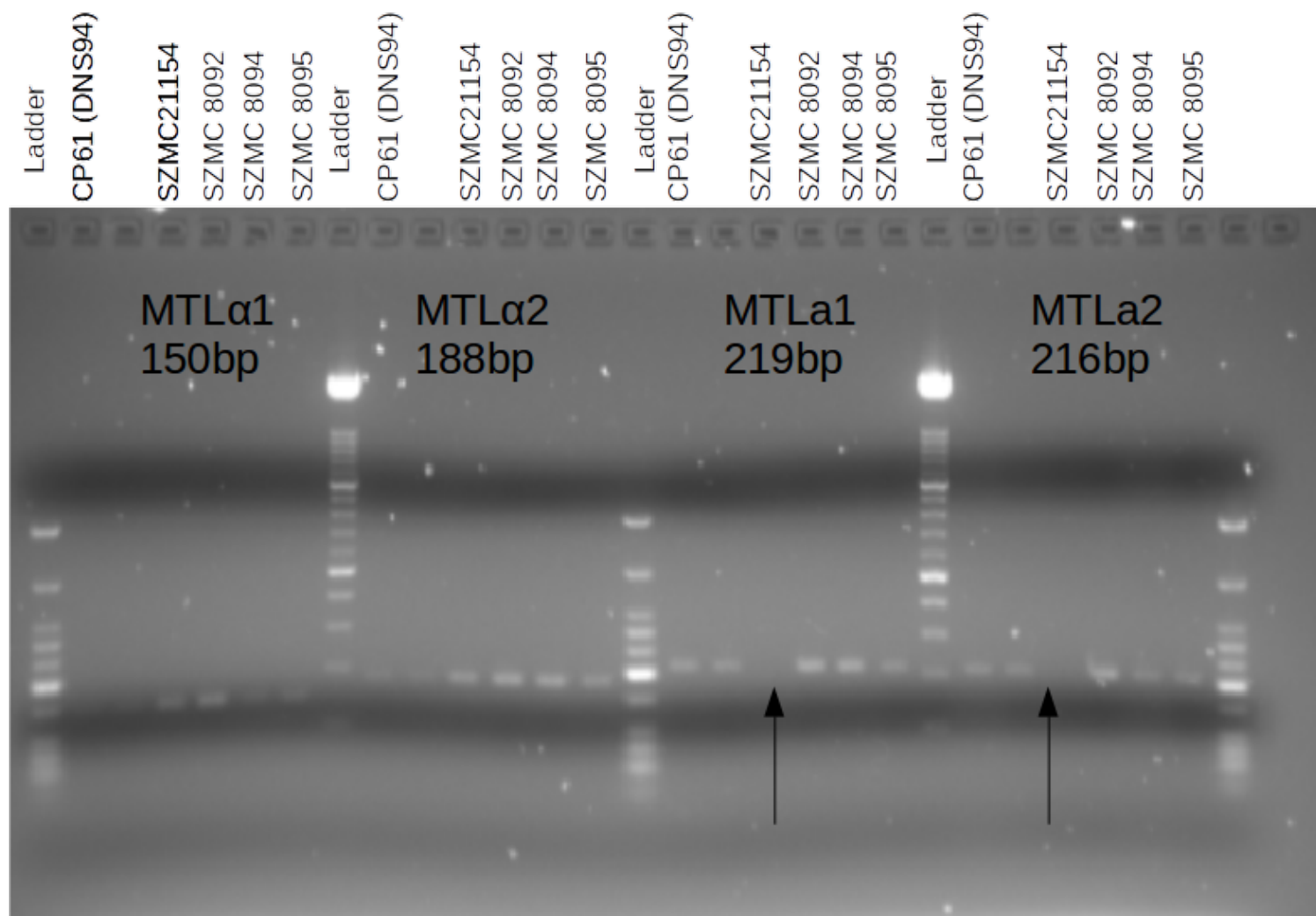

# B

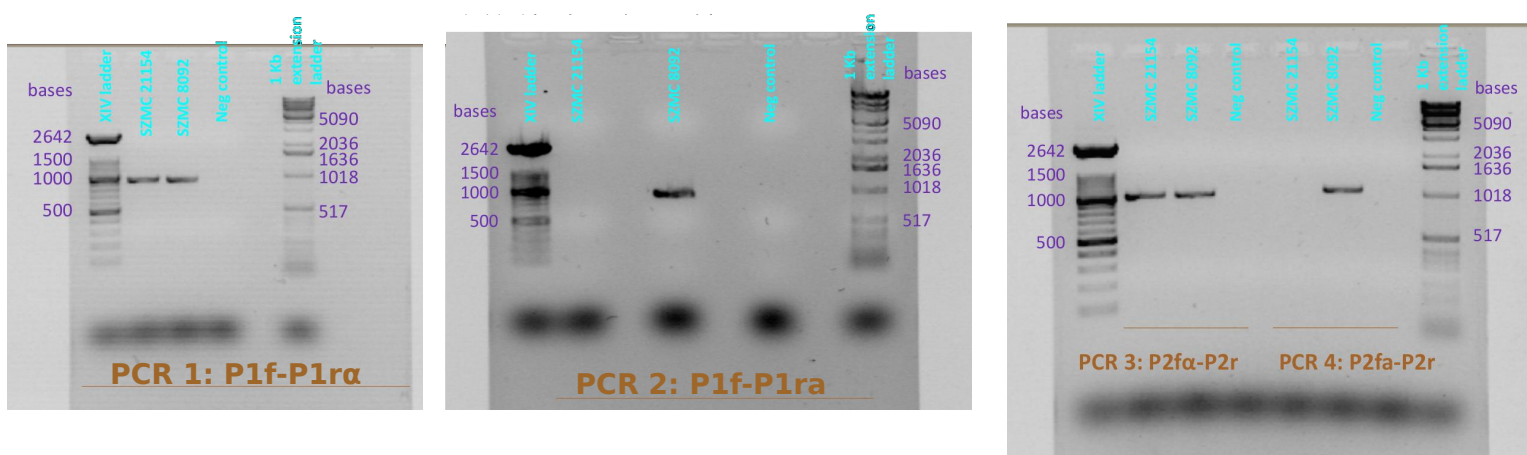

Supplement: S10 Fig — A. We have confirmed presence of MTL a / α idiomorphs in six C. metapsilosis strains by four PCR reactions targeting: MTLα1 (150 bp product), MTLα2 (188 bp product), MTL1a (219 bp product) and MTLa2 (216 bp product). MTLα1 and MTLα2 are present in all tested strains. SZMC21154 does not have the MTLa1 not MTLa2 fragments (marked with arrows). B. We have confirmed linearity of MTL a / α idiomorphs in two C. metapsilosis strains (SZMC8092 and SZMC21154) by four PCR reactions flanking introgression ends: P1f ‐ P1rα (932 bp product), P1f –P1ra (900 bp product), P2fα–P2r (1009 bp product) and P2fa–P2r (1076 bp product). MTLα is present in both strains, thus P1f ‐ P1rα and P2fα–P2r yield PCR product in both strains. P1f –P1ra and P2fa–P2r do not give product in SZMC21154, as MTLa is missing in this strain. (PDF) [file pgen.1005626.s010.pdf]

**A**

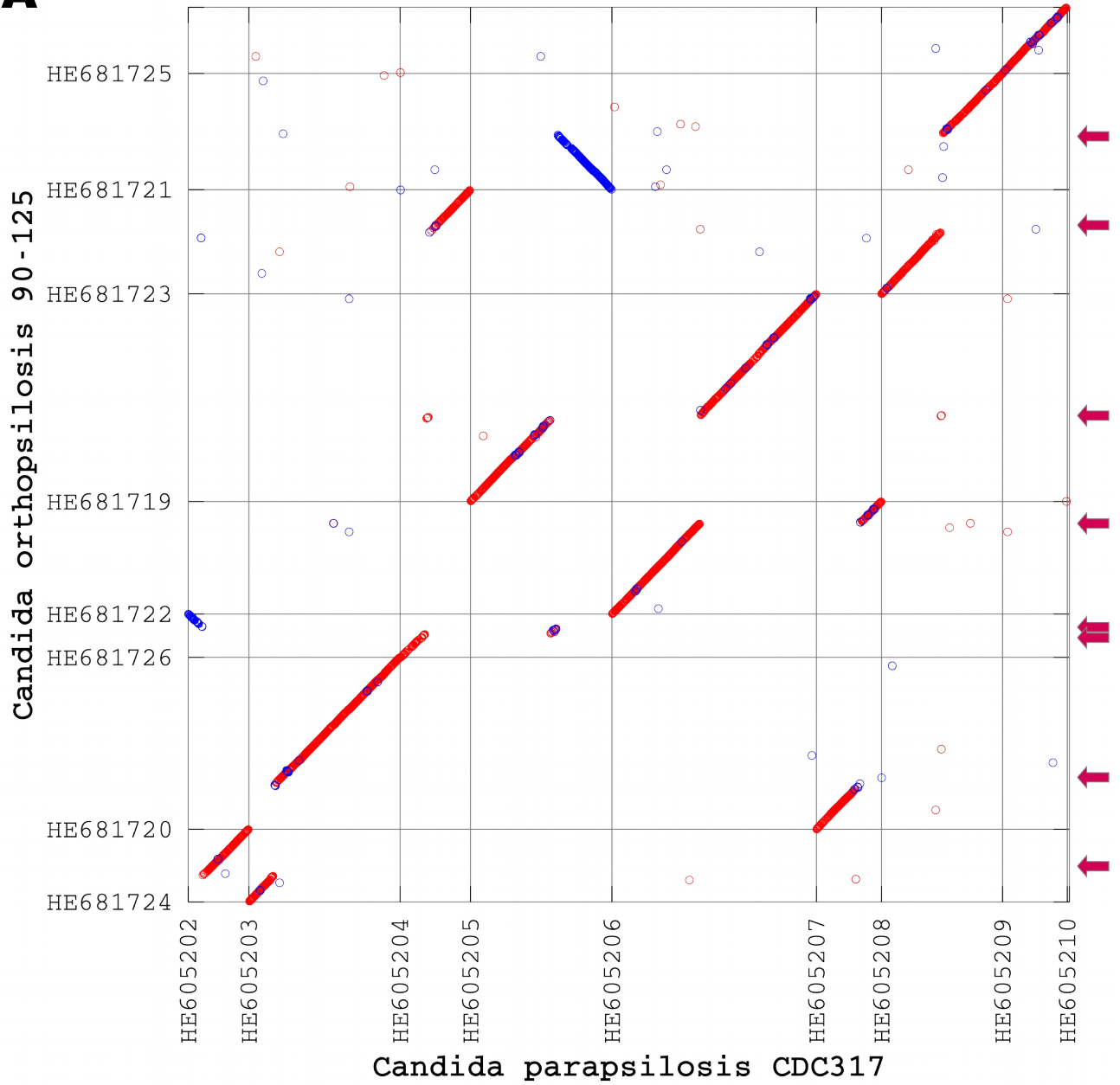

Candida metapsilosis SZMC8094

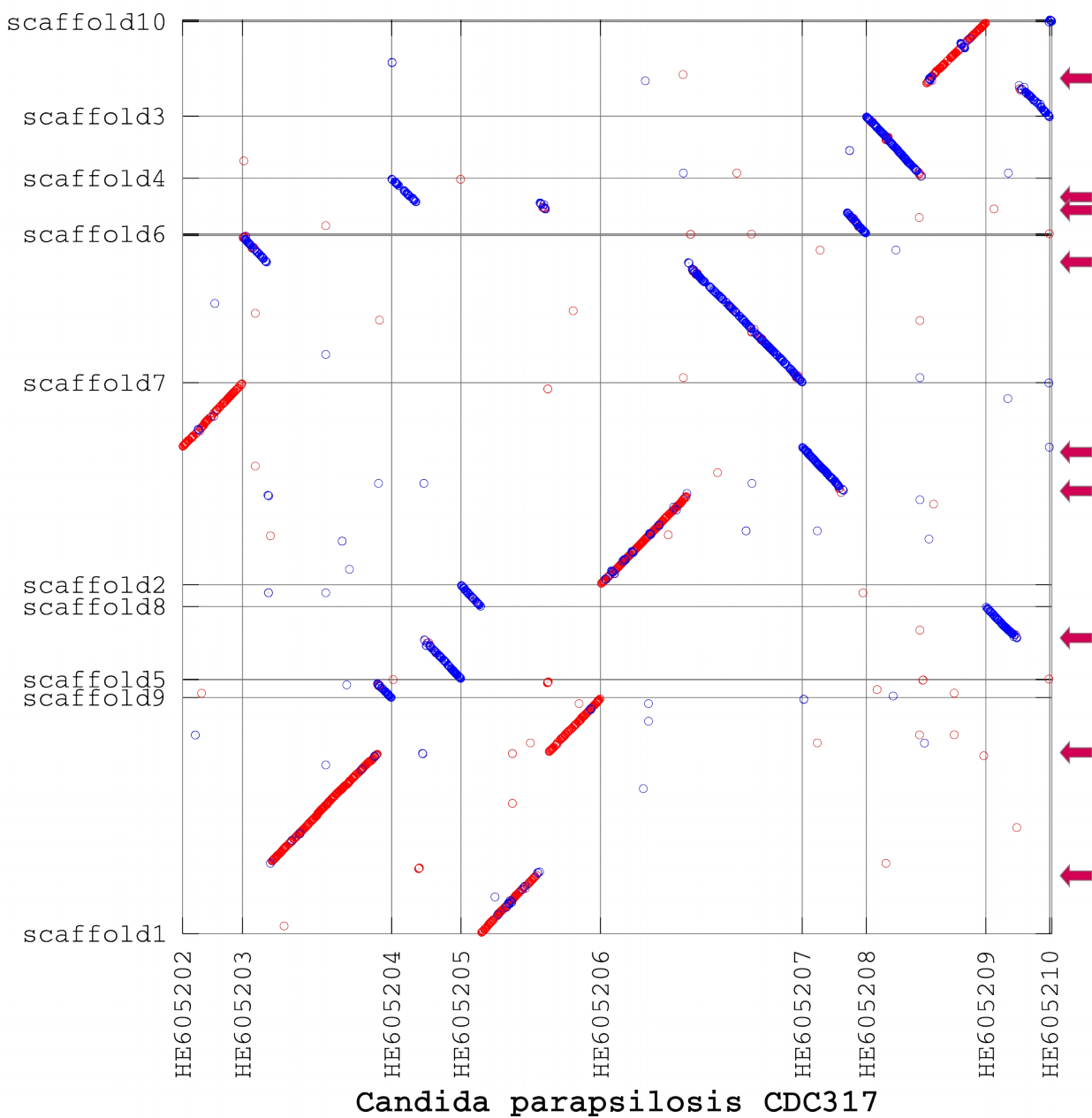

Candida metapsilosis SZMC8094

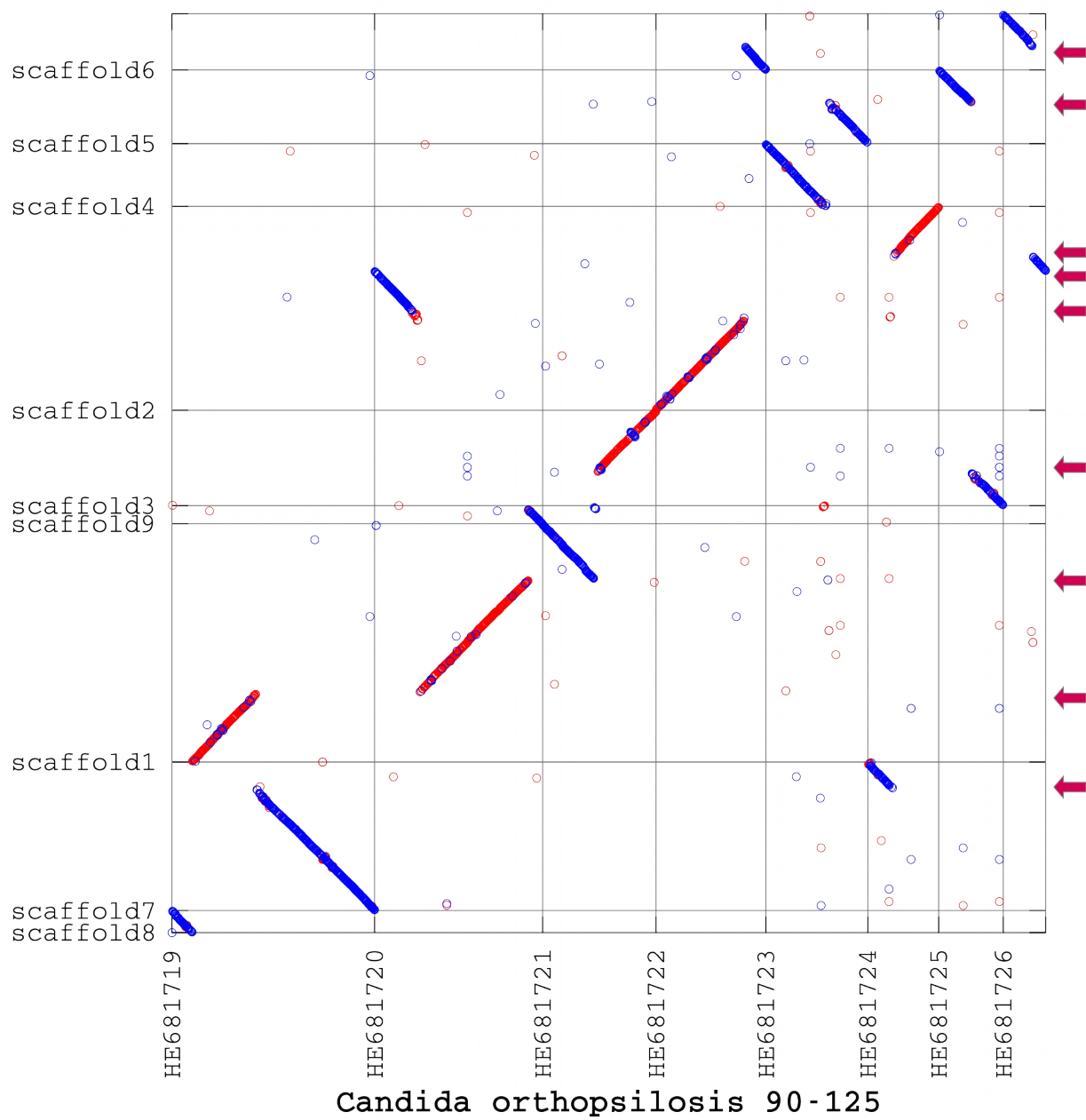

Supplement: S12 Fig — Chromosomes of Candida parapsilosis complex species were aligned using nucmer 3.0.7 (Kurtz et al., 2004): A) C. orthopsilosis 90–125 against C. parapsilosis CDC317, B) C. metapsilosis SZMC8094 against C. parapsilosis CDC317 and C) C. metapsilosis SZMC8094 against C. orthopsilosis 90–125. Forward alignments are colored in red, while reverse alignments in blue. Chromosomes of all three species are syntenic with 8–9 translocations (marked with arrows on the right Y axis) and numerous small inversions. (PDF) [file pgen.1005626.s012.pdf]

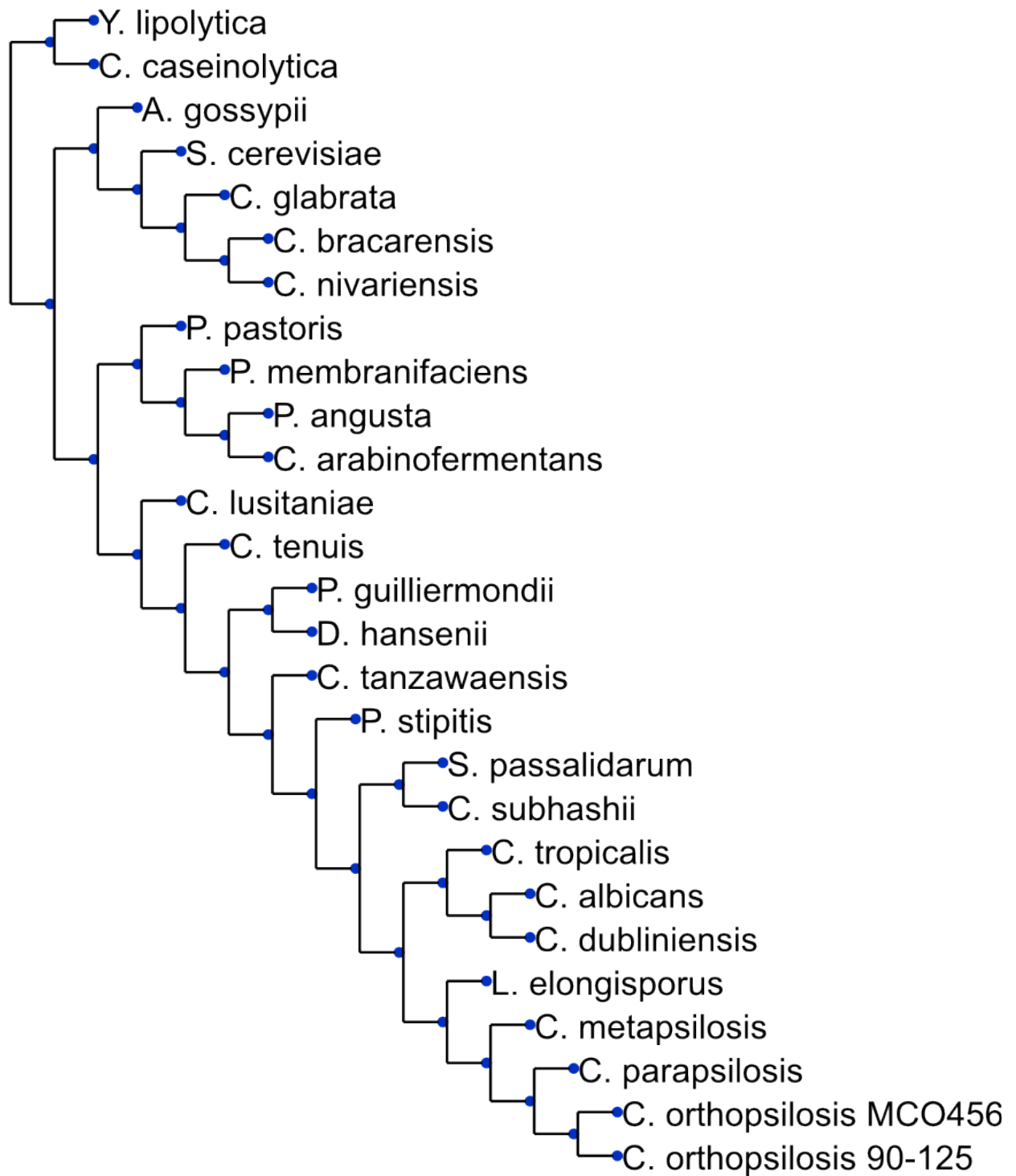

Supplement: S13 Fig — Parsimony-based species tree were reconstructed based on the topologies of 5,780 gene trees from C. metapsilosis phylome (http://phylomedb.org/phylome_243) using duptree v1.48 (Wehe et al., 2008). (PDF) [file pgen.1005626.s013.pdf]

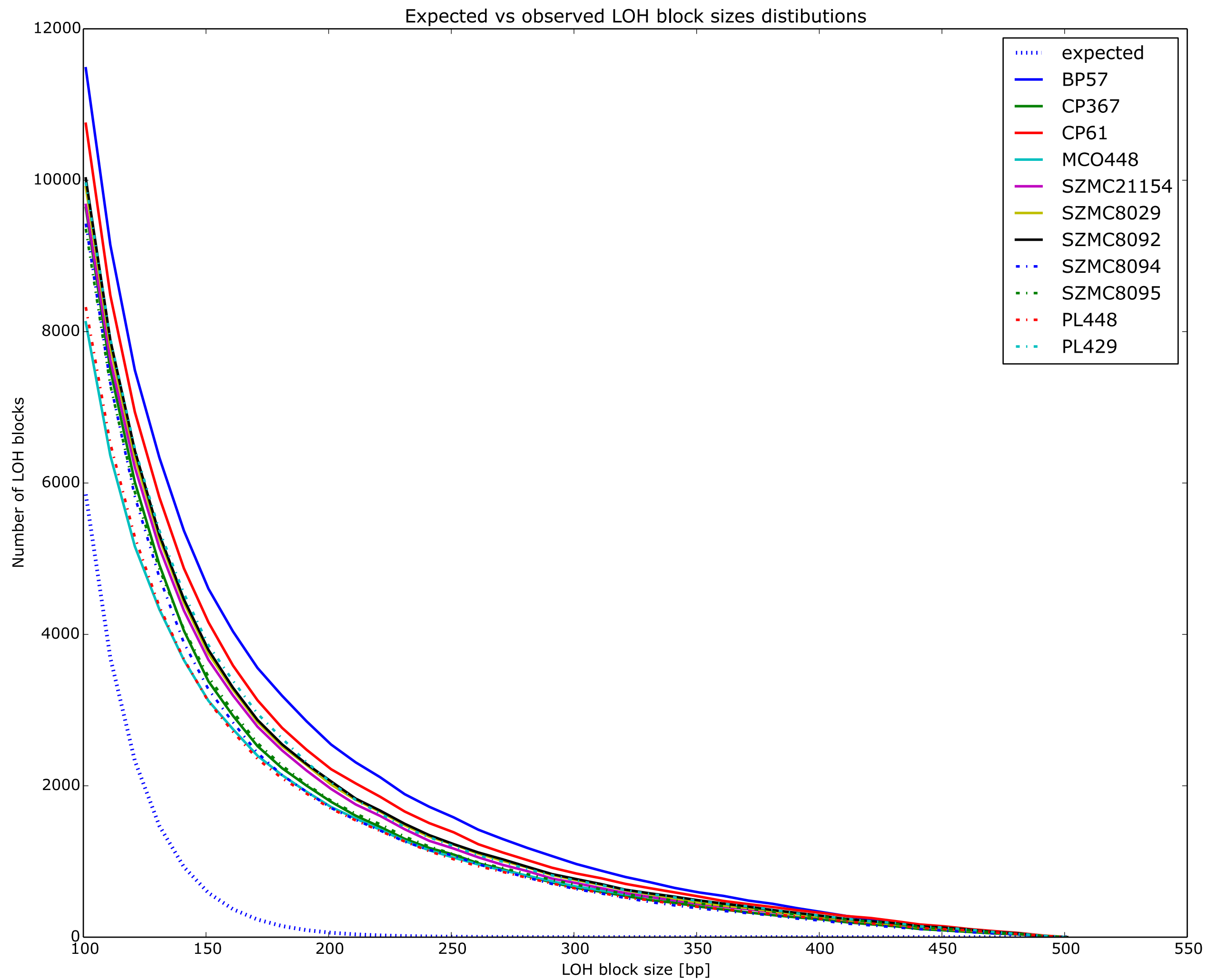

Supplement: S14 Fig — Distributions of expected (blue dashed line) and observed LOH blocks from 11 strains for given block size cut-off were plotted. Expected distribution is approximated as E = N * p * q * k, where N is genome size in basepairs, p and q are the probabilities of having a SNP or not, respectively and k is the required size of the block. (PDF) [file pgen.1005626.s014.pdf]
