# Supplementary material for: The Genomic Aftermath of Hybridization in the Opportunistic Pathogen Candida metapsilosis
Source: PLoS Genet. 2015 Oct 30;11(10):e1005626. doi: 10.1371/journal.pgen.1005626 (PMC4627764; doi:10.1371/journal.pgen.1005626)

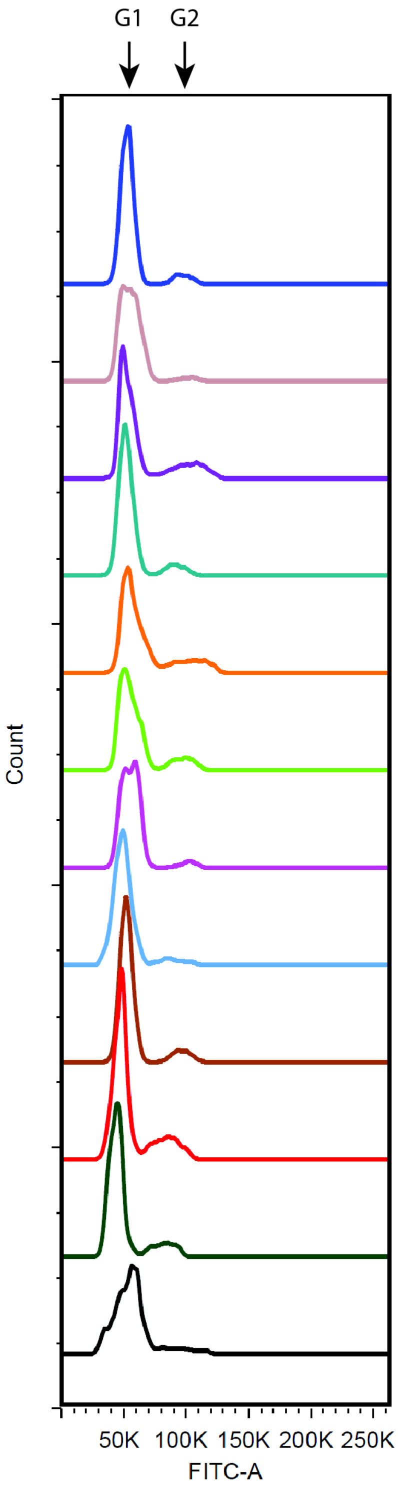

Species	Strain	G1 FITC-A median	G2 FITC-A median	Ratio G2/G1
Candida metapsilosis	SZMC 8092	52.86	97.09	1.84
Candida metapsilosis	SZMC 8093	54.30	102.48	1.89
Candida metapsilosis	SZMC 8094	52.06	103.11	1.98
Candida metapsilosis	SZMC 8095	51.51	91.20	1.77
Candida metapsilosis	SZMC 8098	55.48	105.39	1.90
Candida metapsilosis	SZMC 21154	52.95	97.97	1.85
Candida metapsilosis	SZMC 1548	54.54	101.41	1.86
Candida metapsilosis	SZMC 8022	48.97	88.78	1.81
Candida metapsilosis	SZMC 8029	52.06	96.32	1.85
Candida parapsilosis	GA 1	47.23	83.86	1.78
Candida orthopsilosis	SZMC 1545	43.60	83.00	1.90
Candida albicans	SC 5314	53.34	101.43	1.90

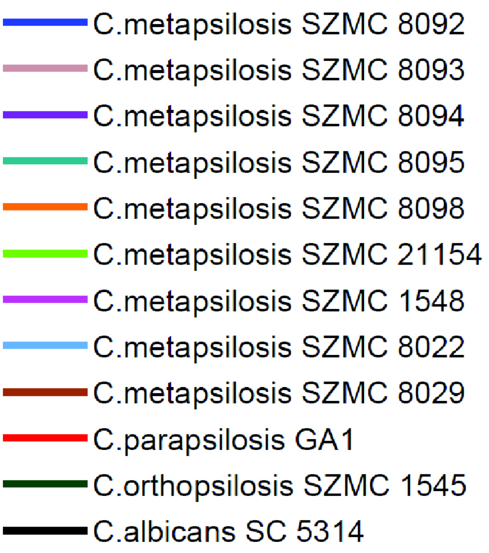

Supplement: S9 Fig — Twelve Candida samples were analyzed using fluorescence-activated cell sorting (FACS): 9 strains of C. metapsilosis, 1 C. parapsilosis, 1 C. orthopsilosis and 1 C. albicans. In the histogram, FITC-A values—fluorescence signal of the fluorochrome fluorescein isothiocyanate—corresponding to DNA content versus cell counts are plotted. Peaks around 50 K and 100 K values of FITC-A account for cells in G1 and G2 phases, respectively. In the table, FITC-A G1 and G2 medians are shown per each of the samples, as well as the ratio between them. (PDF) [file pgen.1005626.s009.pdf]
