# Supplementary material for: The Genomic Aftermath of Hybridization in the Opportunistic Pathogen Candida metapsilosis
Source: PLoS Genet. 2015 Oct 30;11(10):e1005626. doi: 10.1371/journal.pgen.1005626 (PMC4627764; doi:10.1371/journal.pgen.1005626)
Supplement: S11 Fig — Phylogenetic trees were reconstructed from matrices consisting of: A) SNPs in the longest (300kb) homozygous regions (51 patterns), B) concatenated chromosomes with incorporated SNP (3,406 patterns), C1) 13,374 three-state (hapA, hapB, heterozygous) haplotypes in 1 kb windows (1,798 patterns), D) 170 multi-state CNVs: 0 for null deletion, 1 for heterozygous deletion, 2 for wild-type (no deletion and duplication), 3 for duplication (3 copies of given locus), 4 for duplication (4 copies of given locus) etc (127 patterns), and E) 8,889 LOH presence / absence profiles (587 patterns). Bootstrap support values are given if lower than 100. Strains are color-coded, accordingly to the place of isolation. Trees were visualised using iTOL (Letunic & Bork, 2011). Note, here we consider patterns as phylogenetically informative loci, this is alleles that are shared by more than one strain, but not present in all of them. Maximum Likelihood phylogenetic trees were reconstructed from these alignments using RAxML 7.2.8 using GTRCAT model for all except multi-state matrices and GTRGAMMA model for multi-state matrices. (PDF) [file pgen.1005626.s011.pdf]

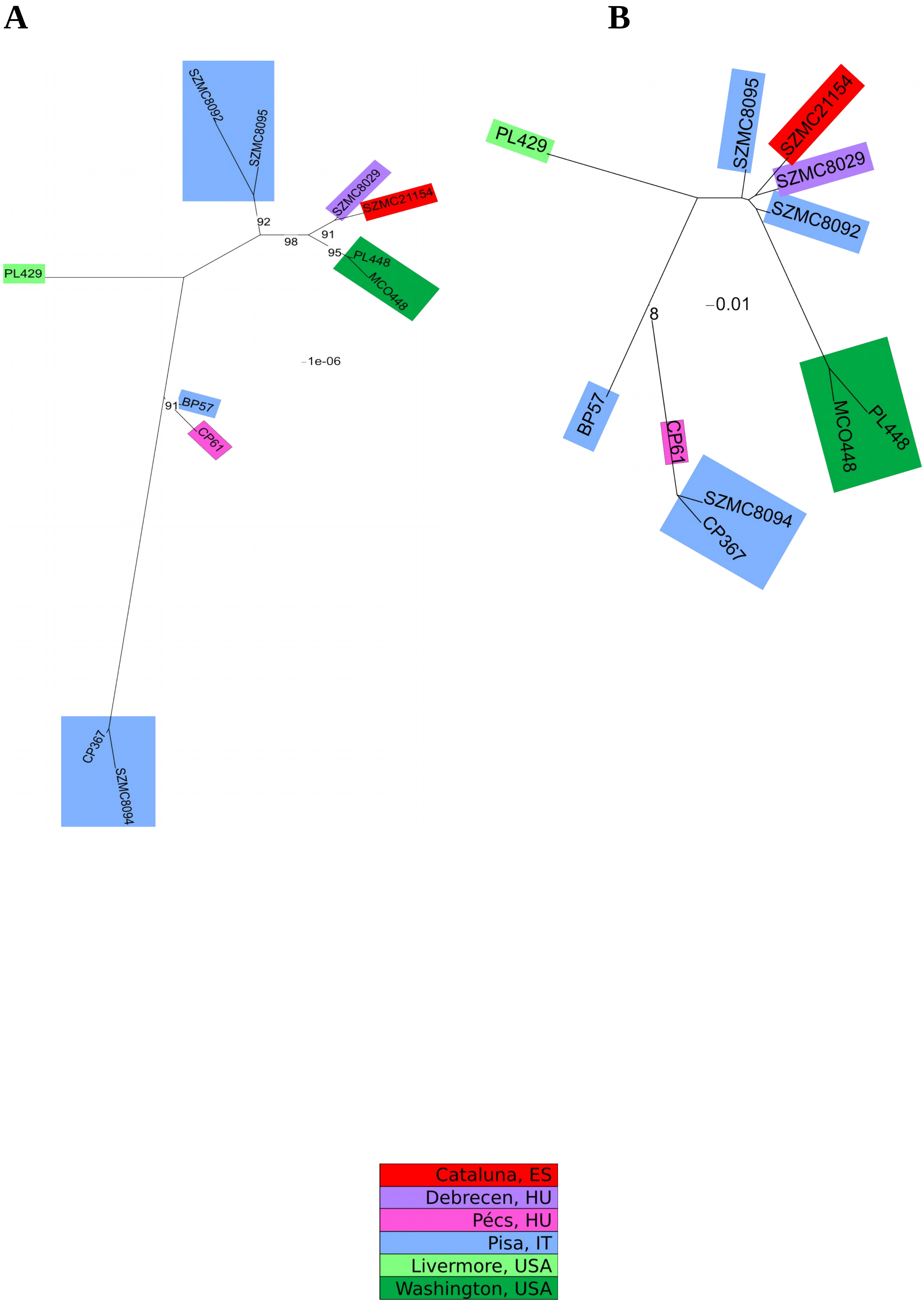

A
B
SZMC8092
SZMC8095
92
98
91
95
SZMC8029
SZMC21154
PL448
MCO448
PL429
1e-06
91
BP57
CP61
CP367
SZMC8094
PL429
SZMC8095
SZMC21154
SZMC8029
SZMC8092
0.01
8
BP57
CP61
SZMC8094
CP367
MCO448
PL448
Cataluna, ES
Debrecen, HU
Pécs, HU
Pisa, IT
Livermore, USA
Washington, USA

C D

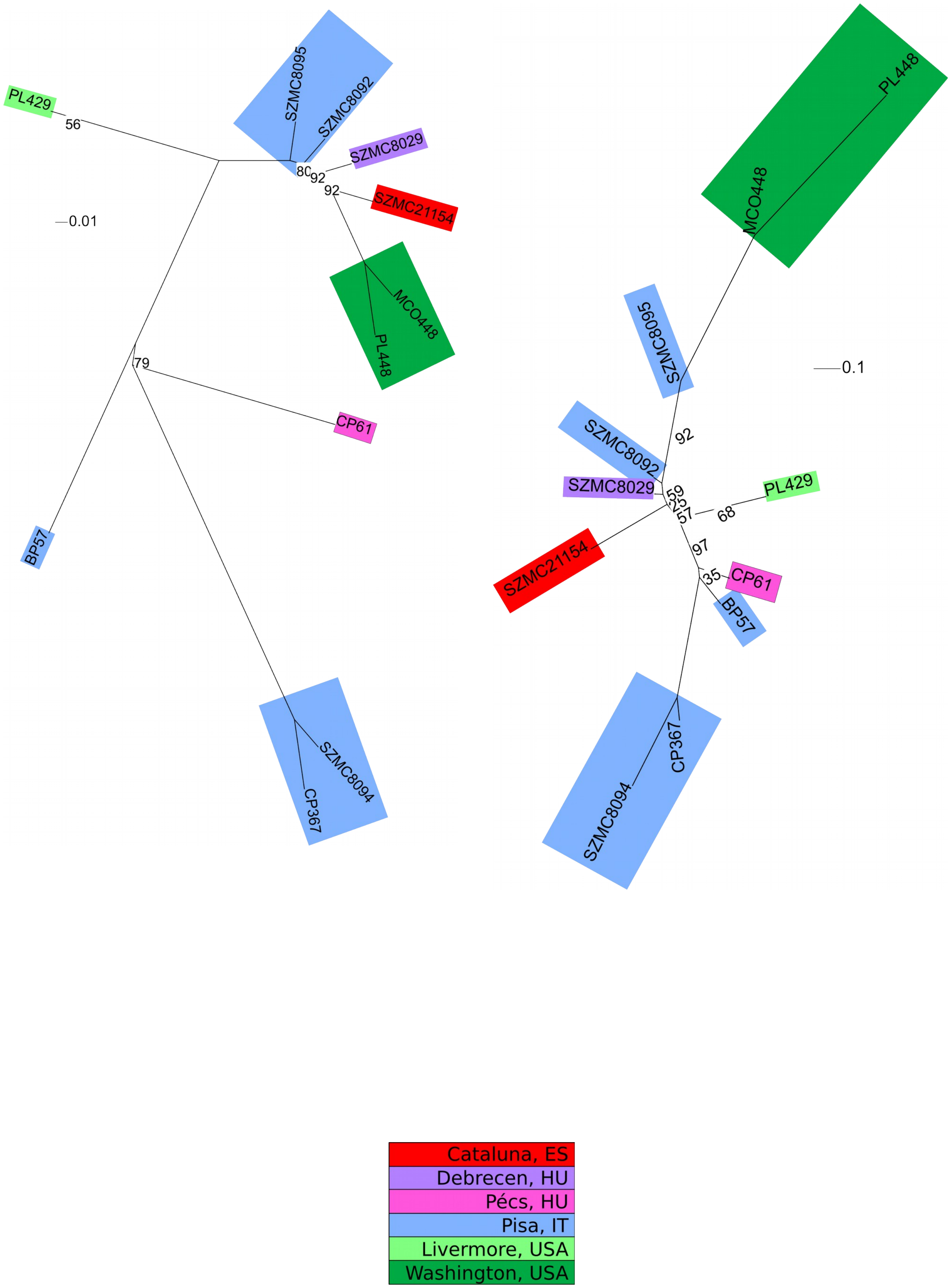
PL429
56
SZMC8095
SZMC8092
SZMC8029
80
92
92
SZMC21154
0.01
MCO448
PL448
79
CP61
BP57
SZMC8094
CP367
PL448
MCO448
SZMC8095
0.1
92
SZMC8092
SZMC8029
59
PL429
57
68
SZMC21154
97
35
CP61
BP57
CP367
SZMC8094
Cataluna, ES
Debrecen, HU
Pécs, HU
Pisa, IT
Livermore, USA
Washington, USA

E

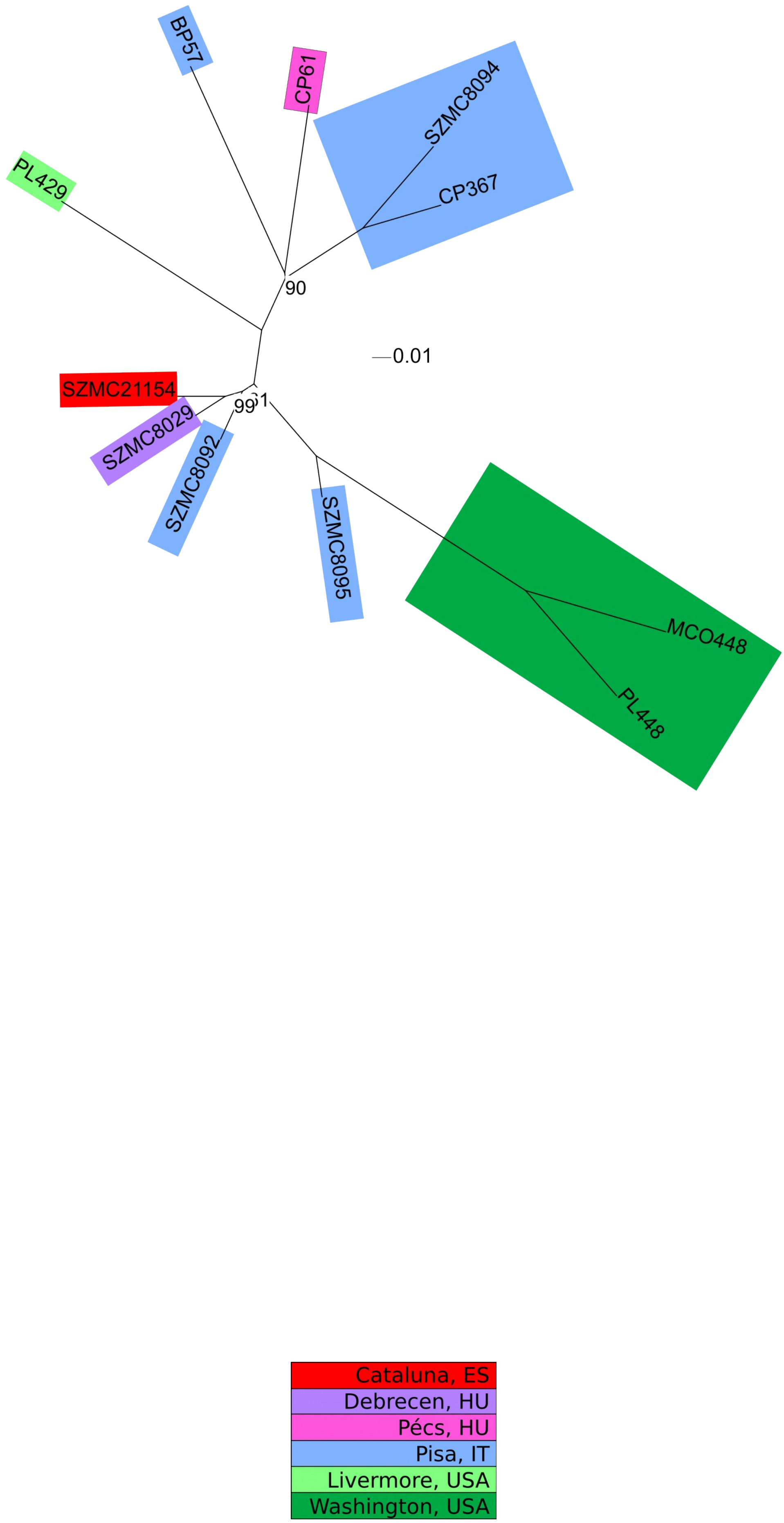
BP57
CP61
SZMC8094
CP367
PL429
90
0.01
SZMC21154
99
SZMC8029
SZMC8092
SZMC8095
MCO448
PL448
Cataluna, ES
Debrecen, HU
Pécs, HU
Pisa, IT
Livermore, USA
Washington, USA
