# Supplementary material for: The Genomic Aftermath of Hybridization in the Opportunistic Pathogen Candida metapsilosis
Source: PLoS Genet. 2015 Oct 30;11(10):e1005626. doi: 10.1371/journal.pgen.1005626 (PMC4627764; doi:10.1371/journal.pgen.1005626)
Supplement: S1 Table — For each C. metapsilosis strain the table provides: its name, available genomic libraries, read length(s), cumulative depth of coverage, k-mer(s) used for assembly, obtained number of contigs, cumulative assembly size, percentage of GC content, number of contigs longer than 1 kb and the cumulative size of these contigs, N50, N90, the cumulative size of gaps and the length of the longest contigs. (PDF) [file pgen.1005626.s015.pdf]

strain	libraries	read length	depth of coverage	k	contigs	bases	GC [%]	contigs >1kb	bases in contigs >1kb	N50	N90	Ns	longest
PL429	pe300	76	412	63	14,975	21,989,545	37.94	4,199	19,092,291	10,535	3,928	513,678	186,352
PL429	pe400ov	250	381	41 - 91	8,342	22,892,786	38.04	3,432	21,184,119	8,890	1,435	30,053	162,895
PL429	pe300; pe400ov	76; ~400	793	31 - 61	7,792	22,742,173	38.04	3,515	21,186,109	8,677	1,535	28,645	177,697
PL429	pe300; pe600; mp5000; pe400ov	76; 46; 46; 250	1,308	31 - 127	24,925	25,093,078	38.04	6,831	19,130,027	2,539	292	0	21,506
BP57	pe600	96	293	41 - 91	29,646	28,329,180	37.91	3,999	22,735,456	7,042	211	903,163	324,054
CP367	pe600	96	265	41 - 91	12,801	24,371,510	37.99	3,291	22,043,223	9,870	1,086	940,164	247,133
CP61	pe600	96	273	41 - 91	20,198	26,547,316	37.93	3,919	22,536,014	7,799	460	875,009	232,624
SZMC21154	pe600	96	569	41 - 91	11,374	23,253,387	37.87	2,169	21,488,133	17,832	1,867	212,077	310,367
SZMC8029	pe600	96	500	41 - 91	10,162	23,352,175	37.92	2,187	21,774,098	18,288	2,242	213,676	294,346
SZMC8092	pe600	96	568	41 - 91	15,400	24,194,700	37.92	2,298	21,598,491	15,444	791	491,088	294,138
SZMC8094	pe600	96	467	41 - 91	10,526	23,742,002	37.98	2,181	22,057,154	18,631	1,991	265,018	294,315
SZMC8095	pe600	96	575	41 - 91	16,237	24,345,721	38.02	2,314	21,655,210	14,867	721	436,559	294,103
MCO448	pe600	46	200	31 - 41	38,268	25,662,059	38.23	4,558	18,912,731	3,704	168	5,621,182	98,928
PL448	pe600	46	212	31 - 41	44,777	24,993,393	38.01	4,255	17,145,966	3,001	153	4,433,373	246,982
