# Supplementary material for: The Genomic Aftermath of Hybridization in the Opportunistic Pathogen Candida metapsilosis
Source: PLoS Genet. 2015 Oct 30;11(10):e1005626. doi: 10.1371/journal.pgen.1005626 (PMC4627764; doi:10.1371/journal.pgen.1005626)
Supplement: S2 Table — For each step of heterozygous assembly the table provides: name, result file name(s), number of contigs, cumulative assembly size, percentage of GC content, number of contigs longer than 1 kb and the cumulative size of these contigs, N50, N90, the cumulative size of gaps and the length of the longest contigs. (PDF) [file pgen.1005626.s016.pdf]

step	file_name	contigs	bases	GC [%]	contigs >1kb	bases in contigs >1kb	N50	N90	Ns	longest contig
SOAP2	SZMC8094.k41-91.scafSeq	10,526	23,742,002	37.98	2,181	22,057,154	18,631	1,991	265,018	294,315
GapCloser	SZMC8094.k41-91.gf1.scafSeq	10,526	23,690,189	37.96	2,181	22,005,328	18,526	1,984	9,127	295,590
HaploMerger	SZMC8094/genome.fa	2,181	22,057,154	38.03	2,181	22,057,154	20,538	4,382	261,056	294,315
HaploMerger	SZMC8094/assembly_hapA.fa	714	9,758,829	38.05	712	9,757,071	23,399	6,516	135,647	123,420
HaploMerger	SZMC8094/assembly_hapB.fa	879	9,078,461	38.04	870	9,070,778	18,136	4,963	101,935	119,989
HaploMerger	SZMC8094/optiNewScaffolds.fa	692	9,803,606	38.05	692	9,803,606	25,041	6,671	135,820	123,420
HaploMerger	SZMC8094/unpaired.fa	628	3,204,737	37.96	612	3,193,430	11,648	1,753	23,082	294,315
HaploMerger	SZMC8094/genome.new.fa	1,320	13,008,343	38.03	1,304	12,997,036	23,367	3,875	158,902	294,315
SSPACE	SZMC8094/ite0.final.scaffolds.fasta	1,320	13,008,343	38.03	1,304	12,997,036	23,367	3,875	158,902	294,315
SSPACE	SZMC8094/ite1.final.scaffolds.fasta	501	13,026,833	38.03	489	13,017,925	49,732	13,855	177,829	323,764
SSPACE	SZMC8094/ite2.final.scaffolds.fasta	501	13,026,833	38.03	489	13,017,925	49,732	13,855	177,829	323,764
SSPACE	SZMC8094/ite2.0.final.scaffolds.fasta	501	13,026,833	38.03	489	13,017,925	49,732	13,855	177,829	323,764
SSPACE	SZMC8094/ite2.1.final.scaffolds.fasta	84	13,401,073	38.03	73	13,392,897	784,089	252,448	552,069	1,447,475
SSPACE	SZMC8094/ite2.2.final.scaffolds.fasta	70	13,416,493	38.03	59	13,408,317	837,157	317,314	567,489	3,446,298
SSPACE	SZMC8094/fosmid.final.scaffolds.fasta	57	13,511,817	38.03	46	13,503,641	2,161,850	820,601	662,813	3,459,122
GapCloser	SZMC8094/fosmid.final.scaffolds.gf0.fasta	57	13,511,817	38.03	46	13,503,641	2,161,850	820,601	662,813	3,459,122
GapCloser	SZMC8094/fosmid.final.scaffolds.gf1.fasta	57	13,386,121	38.00	46	13,378,335	2,137,174	810,252	88,149	3,427,824
GapCloser	SZMC8094/fosmid.final.scaffolds.gf2.fasta	57	13,387,922	38.00	46	13,380,134	2,137,334	810,769	87,055	3,428,185
GapCloser	SZMC8094/fosmid.final.scaffolds.gf2.1.fasta	57	13,392,383	38.00	46	13,384,593	2,138,427	810,870	59,223	3,429,230
GapCloser	SZMC8094/fosmid.final.scaffolds.gf2.2.fasta	57	13,392,600	38.00	46	13,384,808	2,138,593	810,922	57,663	3,429,510
GapCloser	SZMC8094/fosmid.final.scaffolds.gf2.3.fasta	57	13,393,615	38.00	46	13,385,821	2,138,759	810,974	57,662	3,429,874
