# Supplementary material for: The Genomic Aftermath of Hybridization in the Opportunistic Pathogen Candida metapsilosis
Source: PLoS Genet. 2015 Oct 30;11(10):e1005626. doi: 10.1371/journal.pgen.1005626 (PMC4627764; doi:10.1371/journal.pgen.1005626)
Supplement: S3 Table — Estimated ploidy of all chromosomes/scaffolds is provided for all analysed strains. C. metapsilosis is diploid, thus wild-type ploidy of 2 is expected. Chromosomes with deviated ploidy are denoted in red (putative duplication) or blue (putative deletion). Note that rDNA cluster is placed in scaffold5, which therefore shows a larger ploidy in all strains. This, however, does not preclude us to detect triploidy of scaffold5 in PL448. Variability in mitochondrial chromosome (scaffold10) copy number is likely reflecting differences in sample preparation and not true biological variability. (PDF) [file pgen.1005626.s017.pdf]

chromosome [1]	MCO448	PL448	BP57	CP367	CP61	SZMC21154	SZMC8029	SZMC8092	SZMC8094	SZMC8095	PL429 pe300	PL429 pe400ov	PL429 pe600	PL429 mp5000	PL429 fosmid	comments
**scaffold1|size3459122**	1.94	1.80	1.97	1.95	1.97	1.80	1.95	1.97	1.90	2.01	1.92	1.95	1.98	1.98	2.04	
**scaffold2|size2959145**	1.99	**2.16**	1.96	1.96	1.95	**2.39**	1.94	1.95	1.99	1.94	1.90	1.92	1.94	1.96	1.90	
**scaffold7|size2161850**	1.89	1.78	1.94	1.92	1.93	1.75	1.90	1.91	1.89	1.90	1.87	1.90	1.90	1.92	2.09	
**scaffold3|size1388033**	1.91	1.80	1.91	1.92	1.92	1.77	1.90	1.93	1.94	1.92	1.86	1.93	1.87	1.90	1.85	
**scaffold5|size1063617 [2]**	2.39	3.08	2.32	2.34	2.34	2.25	2.32	2.35	2.38	2.36	2.21	2.44	2.24	2.44	2.05	rDNA
**scaffold4|size908642**	1.91	1.79	1.94	1.96	1.94	1.77	1.92	1.93	1.95	1.91	1.87	1.94	1.91	1.94	1.74	
**scaffold6|size820601**	**2.23**	2.03	2.02	2.12	1.99	1.89	2.05	2.04	**2.20**	2.07	2.00	2.07	2.04	2.09	2.38	
**scaffold8|size317314**	**2.26**	2.01	2.00	1.99	1.98	1.88	2.01	2.02	2.16	2.13	1.97	2.03	2.01	2.11	2.15	
**scaffold9|size263853**	2.15	2.06	1.96	1.99	1.96	1.79	1.93	1.96	2.11	1.99	1.93	1.95	1.96	2.04	1.86	
scaffold10|size21864 [3]	1.80	1.99	14.78	8.33	16.18	27.67	24.11	15.26	6.58	9.83	48.78	13.56	23.07	6.87	0.00	mitochondrial chromosome
scaffold11|size20967	2.09	1.81	1.65	1.76	1.74	1.63	1.76	1.80	1.92	2.02	1.71	1.72	1.76	1.84	1.74	
scaffold12|size15030	1.25	1.09	1.22	1.24	1.19	1.11	1.20	1.21	1.30	1.27	1.17	1.23	1.16	0.97	3.89	
scaffold13|size9895	1.75	1.80	1.34	1.98	1.22	2.12	0.38	1.31	1.46	1.27	2.02	1.69	1.76	1.74	0.00	
scaffold14|size8524	1.65	2.30	1.76	1.71	1.73	1.74	1.86	1.83	1.77	1.76	1.84	1.67	1.69	1.79	0.69	
scaffold16|size6993	0.87	1.53	1.95	1.69	1.50	2.89	3.63	2.22	1.24	1.49	3.56	2.61	3.47	2.66	8.39	
scaffold15|size7016	2.02	2.16	1.70	1.91	1.72	1.76	1.62	1.65	1.83	1.86	1.42	1.80	1.34	1.34	0.97	
scaffold17|size5362	2.97	2.88	2.46	3.04	1.07	3.37	1.79	1.66	1.94	1.46	2.79	2.50	2.54	1.92	0.00	
scaffold18|size4846	1.83	2.80	2.77	0.93	1.75	4.72	5.02	5.02	4.73	5.42	0.00	8.87	8.67	1.73	0.00	
scaffold45|size1071	22.49	23.70	9.07	52.99	8.56	7.76	8.98	8.80	48.46	6.70	14.62	9.47	18.46	4.66	12.93	
scaffold48|size920	5.10	5.34	2.00	4.32	0.39	2.76	3.09	2.93	2.99	3.33	1.73	2.42	2.05	0.74	0.00	
scaffold54|size588	2.80	3.35	1.34	5.47	1.08	2.39	1.98	2.85	4.29	1.43	5.50	7.95	2.05	2.98	29.87	
scaffold19|size3739	0.48	1.04	1.55	2.05	1.68	0.26	0.29	0.28	1.97	0.33	1.86	2.67	1.50	2.14	3.13	
scaffold20|size3160	0.76	1.68	1.51	0.85	2.36	1.55	1.59	1.00	0.72	0.88	1.30	0.82	1.25	0.48	0.00	
scaffold21|size3072	1.37	1.31	0.97	1.25	1.00	0.94	0.96	0.96	1.07	1.14	1.06	1.55	0.89	0.91	3.81	
scaffold22|size2988	0.94	0.98	0.87	0.82	0.95	0.92	0.81	1.04	0.98	0.98	1.05	1.03	0.86	0.41	0.00	
scaffold23|size2932	0.33	0.26	1.27	1.40	0.68	1.06	0.71	0.60	1.76	0.70	0.55	3.34	0.15	0.56	29.95	
scaffold24|size2890	0.04	0.03	0.99	1.15	1.00	0.05	0.02	0.02	1.37	0.03	0.03	0.10	0.02	0.03	0.00	
scaffold25|size2889	0.74	0.77	0.95	0.88	0.95	0.69	0.85	0.83	0.90	0.74	0.72	0.68	0.79	0.56	0.00	
scaffold26|size2760	1.22	1.08	0.73	0.82	0.76	0.75	0.85	0.82	0.89	1.03	0.77	1.14	0.64	0.61	0.00	
scaffold27|size2613	0.01	0.01	0.97	1.02	1.00	0.00	0.00	0.00	0.99	0.00	0.01	0.01	0.01	0.01	0.00	
scaffold28|size2552	0.96	0.86	1.08	0.92	1.02	0.00	1.01	1.07	1.06	0.96	0.98	0.96	1.05	0.99	0.00	
scaffold29|size2468	1.53	1.48	1.62	1.49	1.62	2.20	1.59	1.58	1.41	1.51	0.87	0.82	0.79	0.42	2.37	
scaffold30|size2308	0.73	0.93	1.02	0.86	0.98	0.88	0.90	0.94	0.86	0.78	1.06	0.89	1.07	1.00	0.00	
scaffold31|size2209	0.52	0.46	1.64	1.31	1.64	0.73	0.82	0.84	1.58	0.82	0.66	0.78	0.52	0.34	2.65	
scaffold32|size2198	0.22	0.34	0.62	1.00	1.08	0.40	1.10	1.10	1.03	0.91	1.44	0.76	1.27	0.72	0.00	
scaffold33|size2195	1.03	0.98	1.57	0.86	0.39	1.67	1.83	1.00	0.89	0.97	1.10	1.09	1.03	0.82	2.67	
scaffold34|size2195	0.86	0.74	0.84	0.75	0.81	0.72	0.73	0.78	0.77	0.85	0.41	0.43	0.36	0.07	0.00	
scaffold35|size2060	0.99	0.82	0.81	0.88	0.82	0.70	0.77	0.77	0.86	0.82	0.94	0.87	0.82	0.30	0.00	
scaffold36|size1996	0.90	1.60	0.84	0.78	0.85	0.79	1.64	0.87	0.94	0.05	0.91	0.97	0.88	0.73	0.00	
scaffold37|size1869	1.39	1.53	1.08	1.79	1.80	1.88	2.00	2.13	3.00	1.97	1.86	1.80	1.90	1.38	3.13	

scaffold38|size1589	2.24	1.85	0.55	1.54	1.38	1.67	1.72	1.73	1.59	2.09	2.44	2.14	1.91	0.71	0.00
scaffold39|size1516	1.72	1.35	1.17	1.36	1.29	1.68	1.30	1.25	1.39	1.51	1.12	1.12	0.82	0.51	0.00
scaffold40|size1514	0.60	0.55	1.36	1.31	1.27	0.67	0.73	0.68	1.69	0.78	0.93	0.61	0.55	0.43	0.00
scaffold41|size1479	1.84	1.64	1.38	1.70	1.54	1.47	1.54	1.59	1.66	1.69	1.98	2.44	1.37	1.08	0.00
scaffold42|size1291	0.00	0.00	0.69	0.79	0.11	0.00	0.00	0.00	0.93	0.00	0.00	0.01	0.00	0.01	0.00
scaffold43|size1245	1.97	1.72	1.53	1.50	1.38	1.20	1.26	1.34	1.41	1.53	1.67	2.18	1.33	0.79	0.00
scaffold44|size1158	0.00	0.00	0.87	0.98	0.93	0.00	0.00	0.00	1.03	0.00	0.00	0.00	0.00	0.00	0.00
scaffold46|size1011	1.45	1.26	0.83	1.21	1.07	1.02	1.09	1.01	1.11	1.15	1.39	1.70	0.77	1.00	5.79
scaffold47|size994	0.53	0.82	1.27	1.05	1.31	1.27	1.21	1.19	0.66	0.60	1.74	1.46	1.30	1.22	11.78
scaffold49|size918	1.09	1.01	0.95	0.85	0.97	0.86	0.92	0.93	0.89	0.15	1.51	1.41	1.07	1.23	6.38
scaffold50|size882	0.53	0.87	1.41	1.43	0.73	0.71	0.79	0.85	0.83	0.47	0.81	0.74	0.92	1.05	0.00
scaffold51|size796	0.01	0.01	0.69	0.82	0.72	0.00	0.00	0.00	0.86	0.00	0.01	0.00	0.00	0.04	0.00
scaffold52|size792	0.00	0.00	1.08	0.91	1.10	0.00	0.00	0.00	1.02	0.00	0.00	0.00	0.00	0.00	0.00
scaffold53|size677	0.76	1.27	1.36	1.43	1.17	1.31	1.85	1.35	0.81	0.69	1.73	0.84	2.02	2.21	8.59
scaffold55|size569	0.71	1.03	0.96	1.14	1.10	1.09	1.17	1.14	0.83	0.76	1.23	1.06	1.07	1.13	20.58
scaffold56|size522	0.83	0.77	0.78	0.73	0.76	1.28	0.75	0.65	0.65	0.67	1.93	1.91	1.74	1.97	0.00
scaffold57|size518	0.01	0.18	0.05	0.02	0.08	0.03	0.01	0.04	0.00	0.02	0.05	0.02	0.10	1.69	0.00

[1] core chromosomes are in bold
[2] rDNA cluster
[3] mtDNA is highly dependant on DNA isolation technique, if nuclear DNA was enriched, little or no mtDNA is expected to be sequenced
