# Supplementary material for: The Genomic Aftermath of Hybridization in the Opportunistic Pathogen Candida metapsilosis
Source: PLoS Genet. 2015 Oct 30;11(10):e1005626. doi: 10.1371/journal.pgen.1005626 (PMC4627764; doi:10.1371/journal.pgen.1005626)
Supplement: S4 Table — For each event duplication and deletion the table lists: its genomic coordinate and size, number of affected strains and their names; estimations of ploidy for these strains in this region; information whether deletion appears as null in at least one strain; affected genes and their functions. For simplicity, several functional classes are provided with a specific color background. Some selected CNVs among those longer than >5kb are annotated in S1 File. (PDF) [file pgen.1005626.s018.pdf]

#name	chrom	start	end	size	strains no	affected strains	ploidies	null DEL in some	affected genes	functions	comments	LEGEND	Supplementary file 3
**DUP1**	scaffold1|size3459122	56,900	58,200	1,300	11	BP57 CP367 CP61 MCO448 PL429.300 PL429.5000 PL429.600 PL429.miseq PL448 SZMC21154 SZMC8029 SZMC8092 SZMC8094 SZMC8095	3.36 3.71 3.22 5.24 2.89 4.52 3.23 4.28 3.88 3.37 3.73 3.79 4.06 4.51		g4454			Agglutinin-like	
**DUP2**	scaffold1|size3459122	249,200	250,500	1,300	11	BP57 CP367 CP61 MCO448 PL429.300 PL429.5000 PL429.600 PL429.miseq PL448 SZMC21154 SZMC8029 SZMC8092 SZMC8094 SZMC8095	3.86 4.35 3.78 4.92 3.38 3.83 3.83 4.09 3.88 3.48 3.82 3.93 4.36 4.45		g4542			hyphally	
**DUP3**	scaffold1|size3459122	1,111,000	1,116,857	5,857	7	BP57 CP61 PL429.300 PL429.5000 PL429.600 PL429.miseq SZMC21154 SZMC8029 SZMC8092 SZMC8095	2.99 3.29 6.16 6.41 7.45 3.81 4.62 4.63 3.06 2.77		g4914 g4915 g4916	Retrotransposon gag protein; Cyclin PHO80-like		Major facilitator	
**DUP4**	scaffold1|size3459122	1,169,219	1,175,742	6,523	6	BP57 CP61 PL429.300 PL429.5000 PL429.600 PL429.miseq SZMC8029 SZMC8092 SZMC8095	2.72 2.71 3.07 3.26 3.25 3.00 2.55 2.65 2.69		g4937 g4938 g4939 g4940 g4941	Endosulphine; Autophagy-related protein 3; NADH dehydrogenase [ubiquinone] (complex I), 21kDa subunit, fungi		ABC transporter	x
**DUP5**	scaffold1|size3459122	2,355,300	2,356,700	1,400	9	BP57 CP367 CP61 MCO448 PL429.5000 PL429.miseq SZMC8029 SZMC8092 SZMC8094 SZMC8095	3.83 3.75 3.95 2.90 2.67 3.28 2.67 2.76 3.97 3.14		g5513	Major facilitator superfamily domain, general substrate transporter; Sugar/inositol transporter; General substrate transporter; Major facilitator superfamily domain; Sugar transporter, conserved site		Lipase	
**DUP6**	scaffold1|size3459122	2,388,400	2,389,600	1,200	11	BP57 CP367 CP61 MCO448 PL429.300 PL429.5000 PL429.600 PL429.miseq PL448 SZMC21154 SZMC8029 SZMC8092 SZMC8094 SZMC8095	3.50 3.34 3.35 3.76 3.10 3.55 3.17 3.83 3.18 2.97 3.22 3.36 3.22 3.66		g5526	Arsenical pump ATPase, ArsA/Get3; Anion-transporting ATPase-like domain	it's not ARR3	Peptidase	
**DUP7**	scaffold1|size3459122	2,451,223	2,453,302	2,079	9	BP57 CP61 MCO448 PL429.300 PL429.5000 PL429.600 PL429.miseq PL448 SZMC21154 SZMC8029 SZMC8092 SZMC8095	2.73 2.74 2.89 2.76 3.17 2.69 3.08 2.68 2.57 2.68 2.84 2.61		g5556 g5557 g5558	Arrestin-like, N-terminal; Arrestin C-terminal-like domain; Transcription factor TFIIB; Cyclin-like; Transcription factor TFIIB, cyclin-like domain; Zinc finger, GATA-type; Transcription factor TFIIB, conserved site; Zinc finger, TFIIB-type; Monooxygenase, FAD-binding; Ubiquinone biosynthesis hydroxylase, UbiH/UbiF/VisC/COQ6, conserved site; Ubiquinone biosynthesis hydroxylase, UbiH/UbiF/VisC/COQ6; Aromatic-ring hydroxylase-like			
**DUP8**	scaffold1|size3459122	2,762,400	2,764,000	1,600	11	BP57 CP367 CP61 MCO448 PL429.300 PL429.5000 PL429.600 PL429.miseq PL448 SZMC21154 SZMC8029 SZMC8092 SZMC8094 SZMC8095	5.28 4.62 4.83 3.10 4.29 4.33 4.73 4.80 3.29 4.43 4.18 3.21 5.05 4.30		g5701				
**DUP9**	scaffold1|size3459122	2,764,400	2,768,800	4,400	11	BP57 CP367 CP61 MCO448 PL429.300 PL429.5000 PL429.600 PL429.miseq PL448 SZMC21154 SZMC8029 SZMC8092 SZMC8094 SZMC8095	4.13 3.13 3.91 4.70 3.79 3.94 3.76 5.18 4.40 4.05 4.09 3.96 3.31 4.63		g5702 g5703	Protein of unknown function DUF3445			
**DUP10**	scaffold1|size3459122	2,788,300	2,791,400	3,100	7	BP57 CP367 MCO448 PL429.5000 PL429.600 PL429.miseq PL448 SZMC21154 SZMC8029	3.51 5.13 2.91 2.73 3.06 2.89 2.78 1.19 1.37		g5708	Hyphally-regulated cell wall protein, N-terminal	large differences!!		
**DUP11**	scaffold1|size3459122	2,802,900	2,804,900	2,000	9	BP57 CP367 MCO448 PL429.300 PL429.5000 PL429.600 PL448 SZMC8029 SZMC8092 SZMC8094 SZMC8095	2.75 3.78 6.37 1.14 1.44 1.23 6.60 2.75 4.11 2.75 3.13		g5710	Hyphally-regulated cell wall protein, N-terminal	large differences!!		
**DUP12**	scaffold1|size3459122	2,810,500	2,812,800	2,300	9	BP57 CP367 MCO448 PL429.300 PL429.5000 PL429.600 PL429.miseq SZMC21154 SZMC8029 SZMC8092 SZMC8094 SZMC8095	3.31 6.48 2.54 3.59 4.57 3.76 6.01 3.23 3.78 3.51 4.47 4.44		g5711	Hyphally-regulated cell wall protein, N-terminal			
**DUP13**	scaffold1|size3459122	3,125,899	3,127,684	1,785	4	BP57 MCO448 PL429.5000 PL429.miseq PL448	1.42 3.98 2.54 2.55 3.25		g5835 g5836	Vanillyl-alcohol oxidase, C-terminal subdomain 2; FAD-binding, type 2; FAD-linked oxidase-like, C-terminal; FAD linked oxidase, N-terminal; FAD-linked oxidase, FAD-binding, subdomain 2; FAD-linked oxidase, C-terminal; FAD-binding, type 2, subdomain 1; FAD-linked oxidase, C-terminal; FAD-binding, type 2; FAD-linked oxidase-like, C-terminal; FAD linked oxidase, N-terminal; FAD-linked oxidase, FAD-binding, subdomain 2; FAD-binding, type 2, subdomain 1			
**DUP14**	scaffold1|size3459122	3,206,500	3,208,400	1,900	11	BP57 CP367 CP61 MCO448 PL429.300 PL429.5000 PL429.600 PL429.miseq PL448 SZMC21154 SZMC8029 SZMC8092 SZMC8094 SZMC8095	4.28 3.32 4.64 5.81 5.52 6.16 5.76 6.61 5.18 6.18 5.73 6.04 4.08 7.48		g5874	DDE superfamily endonuclease, CENP-B-like			
**DUP15**	scaffold1|size3459122	3,257,264	3,261,830	4,566	5	CP367 PL429.300 PL429.5000 PL429.600 PL429.miseq PL448 SZMC8092 SZMC8094	2.76 3.46 3.87 3.59 3.19 1.42 1.41 2.58		g5899 g5901 g5900	Major facilitator superfamily domain, general substrate transporter; General substrate transporter; Major facilitator superfamily domain; Major facilitator superfamily domain, general substrate transporter; Major facilitator superfamily; Major facilitator superfamily domain; Major facilitator superfamily domain, general substrate transporter; General substrate transporter; Major facilitator superfamily domain			
**DUP16**	scaffold1|size3459122	3,371,965	3,374,355	2,390	11	BP57 CP367 CP61 MCO448 PL429.5000 PL448 SZMC21154 SZMC8029 SZMC8092 SZMC8094 SZMC8095	5.09 5.87 6.62 10.49 2.67 9.80 7.91 8.41 7.83 6.56 10.71		g5951				
**DUP17**	scaffold1|size3459122	3,410,900	3,412,000	1,100	5	MCO448 PL429.5000 PL429.miseq PL448 SZMC8094 SZMC8095	3.44 2.62 2.59 3.11 2.70 2.67		g5968	Eisosome protein 1			
**DUP18**	scaffold1|size3459122	3,419,000	3,428,400	9,400	11	BP57 CP367 CP61 MCO448 PL429.300 PL429.5000 PL429.600 PL448 SZMC21154 SZMC8029 SZMC8092 SZMC8094 SZMC8095	2.98 3.20 4.03 4.62 2.52 3.01 2.96 5.35 3.34 3.66 3.60 3.30 3.65		g5972 g5973	Leucine-rich repeat; Leucine rich repeat 4			

**DUP19**	scaffold2|size2959145	23,500	25,400	1,900	6	CP367 MCO448 PL429.5000 PL429.miseq PL448 SZMC21154 SZMC8094	2.56 3.48 2.83 2.67 3.89 2.75 2.51		g3144 g3145	RNase P, subunit Pop3; RNA polymerase, alpha subunit; RNA polymerase II, heptapeptide repeat, eukaryotic; RNA polymerase, N-terminal; RNA polymerase Rpb1, domain 4; RNA polymerase Rpb1, domain 5; RNA polymerase Rpb1, domain 6; RNA polymerase Rpb1, domain 7; RNA polymerase Rpb1, domain 1; RNA polymerase Rpb1, domain 3			
**DUP20**	scaffold2|size2959145	2,001,000	2,003,800	2,800	11	BP57 CP367 CP61 MCO448 PL429.300 PL429.5000 PL429.600 PL429.miseq PL448 SZMC21154 SZMC8029 SZMC8092 SZMC8094 SZMC8095	4.15 4.00 4.13 4.76 3.12 1.32 3.56 2.51 7.65 3.93 5.02 4.59 2.73 4.57		.				
**DUP21**	scaffold2|size2959145	2,004,000	2,015,400	11,400	4	MCO448 PL448 SZMC8094 SZMC8095	3.41 4.61 2.73 2.64		g4040 g4041 g4042 g4043 g4044	Leucine rich repeat 4; Major facilitator superfamily domain, general substrate transporter; Major facilitator superfamily; Major facilitator superfamily domain; Leucine-rich repeat; Leucine rich repeat 4; ATPase, P-type, K/Mg/Cd/Cu/Zn/Na/Ca/Na/H-transporter; ATPase, P-type, cytoplasmic domain N; ATPase, P-type cation-transporter, N-terminal; ATPase, P-type, cytoplasmic transduction domain A; ATPase, P-type phosphorylation site; ATPase, P-type, ATPase-associated domain; Haloacid dehalogenase-like hydrolase; ATPase, P-type, H+ transporting proton pump; ATPase, P-type cation-transporter, C-terminal; HAD-like domain; ATPase, cation-transporting, domain N; ATPase, P-type, potassium/sodium efflux, fungal; ATPase, P-type, transmembrane domain			
**DUP22**	scaffold2|size2959145	2,015,900	2,024,700	8,800	3	MCO448 PL429.5000 PL448	2.71 2.50 3.99		g4045 g4046 g4047 g4048 g4049	Domain of unknown function DUF3818, PX-associated; PX-associated domain; Phox homologous domain; GTP binding domain; Nucleolar GTP-binding protein 2; Nucleolar GTP-binding protein 2, N-terminal domain; GTP-binding protein, orthogonal bundle domain; Ribonuclease H-like domain; Ribonuclease CAF1; Protein of unknown function DUF284, transmembrane eukaryotic; EF-Hand 1, calcium-binding site; EF-hand-like domain; Calponin homology domain; EF-HAND 2; Actinin-type, actin-binding, conserved site			
**DUP23**	scaffold2|size2959145	2,038,500	2,046,200	7,700	3	MCO448 PL429.5000 PL448	2.81 2.56 3.72		g4055 g4056 g4057 g4058 g4059	Leucine-rich repeat; Leucine-rich repeat, typical subtype; Leucine rich repeat 4; Aldolase-type TIM barrel; Cystathionine beta-synthase, core; WD40-repeat-containing domain; WD40/YVTN repeat-like-containing domain; WD40-repeat-containing domain; WD40 repeat, conserved site; NAD(P)-binding domain; Short-chain dehydrogenase/reductase, conserved site; Glucose/ribitol dehydrogenase; Short-chain dehydrogenase/reductase SDR			
**DUP24**	scaffold2|size2959145	2,052,400	2,057,100	4,700	3	MCO448 PL448 SZMC8094	2.63 3.70 2.56		g4062 g4063 g4064	Craniofacial development protein 1/Bucentaur; Leucine-rich repeat; Dipeptidyl-peptidase 3			
**DUP25**	scaffold2|size2959145	2,057,400	2,059,100	1,700	4	MCO448 PL429.5000 PL448 SZMC8094	2.65 2.60 3.52 2.63		g4064 g4065	Dipeptidyl-peptidase 3; ABC transporter, transmembrane domain; ABC transporter-like; ABC transporter, conserved site; ABC transporter, integral membrane type 1; ABC transporter, transmembrane domain, type 1; AAA+ ATPase domain			
**DUP26**	scaffold2|size2959145	2,059,400	2,060,800	1,400	5	MCO448 PL429.5000 PL429.miseq PL448 SZMC8094 SZMC8095	2.98 2.69 2.64 3.92 2.81 2.54		g4065	ABC transporter, transmembrane domain; ABC transporter-like; ABC transporter, conserved site; ABC transporter, integral membrane type 1; ABC transporter, transmembrane domain, type 1; AAA+ ATPase domain			
**DUP27**	scaffold2|size2959145	2,061,800	2,063,700	1,900	2	PL448 SZMC8094	3.71 2.78		g4066	DNA polymerase V; Armadillo-type fold; ATPase, F1 complex, epsilon subunit, mitochondrial			
**DUP28**	scaffold2|size2959145	2,064,000	2,065,500	1,500	5	CP367 MCO448 PL429.miseq PL448 SZMC8094	2.52 2.78 2.61 3.84 2.77		g4066 g4067	DNA polymerase V; Armadillo-type fold; ATPase, F1 complex, epsilon subunit, mitochondrial; WD40/YVTN repeat-like-containing domain; WD40 repeat; WD40-repeat-containing domain			
**DUP29**	scaffold2|size2959145	2,075,100	2,078,600	3,500	3	MCO448 PL429.5000 PL448	2.81 2.68 3.82		g4072 g4073 g4074	NAD(P)-binding domain; Short-chain dehydrogenase/reductase, conserved site; Short-chain dehydrogenase/reductase SDR; Glucose/ribitol dehydrogenase; Ribonuclease H-like domain; Ribosomal protein L9/RNase H1, N-terminal; Ribonuclease H domain; Ribonuclease H1, eukaryote; Ribonuclease H1, N-terminal			
**DUP30**	scaffold2|size2959145	2,078,900	2,080,900	2,000	11	BP57 CP367 CP61 MCO448 PL429.300 PL429.5000 PL429.600 PL429.miseq PL448 SZMC21154 SZMC8029 SZMC8092 SZMC8094 SZMC8095	4.90 3.54 5.08 5.78 5.89 7.75 6.45 5.82 5.42 6.90 5.91 6.56 4.31 7.24		g4075	DDE superfamily endonuclease, CENP-B-like			
**DUP31**	scaffold2|size2959145	2,272,300	2,273,400	1,100	3	MCO448 PL429.5000 PL448	2.71 2.60 3.58		g4139	Major sperm protein; PapD-like; Vesicle-associated membrane protein			
**DUP32**	scaffold2|size2959145	2,278,200	2,279,300	1,100	6	CP367 MCO448 PL429.5000 PL429.miseq PL448 SZMC8094 SZMC8095	2.56 3.10 3.10 2.92 4.05 2.54 2.68		g4141	Amino acid transporter, transmembrane			
**DUP33**	scaffold2|size2959145	2,309,300	2,310,600	1,300	4	MCO448 PL429.miseq PL448 SZMC8095	2.72 2.90 3.61 2.56		g4153	Arrestin-like, N-terminal; Immunoglobulin E-set; Arrestin C-terminal-like domain			
**DUP34**	scaffold2|size2959145	2,332,400	2,333,800	1,400	11	BP57 CP367 CP61 MCO448 PL429.300 PL429.5000 PL429.600 PL429.miseq PL448 SZMC21154 SZMC8029 SZMC8092 SZMC8094 SZMC8095	8.77 10.92 6.15 7.15 3.85 4.44 4.20 7.74 11.56 4.58 5.34 5.25 7.47 6.07		g4164	Adhesion domain, bacterial; Agglutinin-like protein, N-terminal			
**DUP35**	scaffold2|size2959145	2,457,900	2,460,200	2,300	6	MCO448 PL429.miseq PL448 SZMC8092 SZMC8094 SZMC8095	3.35 2.62 4.52 2.51 2.66 2.95		g4228 g4229 g4230	Glycine cleavage T-protein, N-terminal; Glycine cleavage T-protein, C-terminal barrel; Glycine cleavage system T protein; PIK-related kinase, FATC; Telomere-length maintenance and DNA damage repair; PIK-related kinase; ATM/Tel1; Phosphatidylinositol 3-/4-kinase, catalytic domain; Protein kinase-like domain; Phosphatidylinositol 3/4-kinase, conserved site			
**DUP36**	scaffold2|size2959145	2,493,100	2,494,700	1,600	3	MCO448 PL429.5000 PL429.miseq PL448	3.01 2.82 2.63 3.81		g4234 g4235	Major facilitator superfamily domain, general substrate transporter; Major facilitator superfamily			

**DUP37**	scaffold2|size2959145	2,500,200	2,502,100	1,900	2	CP367 PL448	2.55 3.72		g4238 g4239 g4240	Ubiquinol-cytochrome c chaperone, CBP3; Ubiquinol-cytochrome c chaperone/UPF0174; Acyl-CoA N-acyltransferase; SKI-interacting protein SKIP, SNW domain; SKI-interacting protein, SKIP			
**DUP38**	scaffold2|size2959145	2,542,800	2,543,900	1,100	3	CP367 PL429.miseq PL448	2.94 2.58 3.65		g4258 g4259	WD40/YVTN repeat-like-containing domain; WD40 repeat, conserved site; G-protein beta WD-40 repeat; WD40-repeat-containing domain; WD40 repeat; Protein kinase-like domain; Protein kinase, ATP binding site; Protein kinase, catalytic domain; Serine/threonine- / dual-specificity protein kinase, catalytic domain			
**DUP39**	scaffold2|size2959145	2,556,600	2,558,800	2,200	3	MCO448 PL429.5000 PL429.miseq PL448	2.76 2.64 2.62 4.01		g4263	Zinc finger, LIM-type			
**DUP40**	scaffold2|size2959145	2,566,900	2,569,400	2,500	4	MCO448 PL429.5000 PL448 SZMC8095	2.96 2.51 3.57 2.56		g4267 g4268 g4269	YL1 nuclear; YL1 nuclear, C-terminal; Ubiquinone biosynthesis O-methyltransferase; Snf7			
**DUP41**	scaffold2|size2959145	2,641,300	2,642,500	1,200	4	CP367 MCO448 PL429.300 PL429.5000 PL429.600 PL429.miseq PL448	2.71 2.76 2.53 2.80 2.50 2.72 3.84		g4299	Protein kinase, ATP binding site; Protein kinase-like domain; Serine/threonine-protein kinase, active site; Protein kinase, catalytic domain; Serine/threonine- / dual-specificity protein kinase, catalytic domain			
**DUP42**	scaffold2|size2959145	2,650,300	2,651,400	1,100	4	MCO448 PL429.5000 PL429.miseq PL448 SZMC8095	3.19 2.60 2.88 3.72 2.57		g4301	Lipase, class 3			
**DUP43**	scaffold2|size2959145	2,687,700	2,689,000	1,300	4	MCO448 PL429.5000 PL429.miseq PL448 SZMC8095	2.87 2.56 2.89 3.51 2.60		g4316	Major facilitator superfamily domain, general substrate transporter; Major facilitator superfamily; Major facilitator superfamily domain			
**DUP44**	scaffold2|size2959145	2,751,600	2,752,800	1,200	1	PL448	3.28		g4350	DNA topoisomerase I, domain 1; Topoisomerase I C-terminal domain; DNA breaking-rejoining enzyme, catalytic core; DNA topoisomerase I, catalytic core, alpha/beta subdomain, eukaryotic-type; DNA topoisomerase I, catalytic core, alpha-helical subdomain, eukaryotic-type; DNA topoisomerase I, catalytic core, eukaryotic-type; DNA topoisomerase I, active site; DNA topoisomerase I, DNA binding, eukaryotic-type; DNA topoisomerase I, DNA binding, mixed alpha/beta motif, eukaryotic-type; DNA topoisomerase I; DNA topoisomerase I, eukaryotic-type			
**DUP45**	scaffold2|size2959145	2,766,000	2,767,700	1,700	2	MCO448 PL448	2.54 3.80		g4354	Zinc finger C2H2-type/integrase DNA-binding domain			
**DUP46**	scaffold2|size2959145	2,770,000	2,775,200	5,200	2	MCO448 PL448	2.92 3.52		g4355 g4356 g4357	Zinc finger C2H2-type/integrase DNA-binding domain; GRAM			
**DUP47**	scaffold2|size2959145	2,790,600	2,791,900	1,300	3	MCO448 PL448 SZMC8094	2.75 3.37 2.61		g4367	Ribosomal protein L1; Ribosomal protein L1, 3-layer alpha/beta-sandwich; Ribosomal protein L1, superfamily			
**DUP48**	scaffold2|size2959145	2,821,600	2,825,700	4,100	3	MCO448 PL429.5000 PL448	2.90 2.53 3.58		g4383 g4384 g4385 g4386	Glycolipid transfer protein domain; Complex 1 LYR protein; ACN9			
**DUP49**	scaffold2|size2959145	2,826,600	2,827,800	1,200	6	BP57 CP61 MCO448 PL429.5000 PL448 SZMC21154	1.34 1.36 3.03 2.71 3.99 1.12		g4387				
**DUP50**	scaffold2|size2959145	2,833,300	2,835,500	2,200	4	MCO448 PL429.5000 PL448 SZMC8094	2.87 2.64 3.68 2.58		g4389	Zinc finger, RING-type; Zinc finger, RING/FYVE/PHD-type			
**DUP51**	scaffold2|size2959145	2,836,500	2,838,100	1,600	2	MCO448 PL448	2.58 3.69		g4390	Helix-loop-helix domain			
**DUP52**	scaffold2|size2959145	2,844,600	2,845,800	1,200	4	MCO448 PL429.5000 PL448 SZMC8094	2.65 2.71 4.13 2.57		g4391				
**DUP53**	scaffold2|size2959145	2,849,400	2,851,000	1,600	4	MCO448 PL429.5000 PL448 SZMC8094	2.50 2.65 3.58 2.62		g4392	V-type proton ATPase subunit a; ATPase, V0/A0 complex subunit a			
**DUP54**	scaffold2|size2959145	2,851,500	2,853,300	1,800	4	MCO448 PL448 SZMC8094 SZMC8095	2.85 3.69 2.66 2.53		g4393 g4392	Tetratricopeptide-like helical; Sel1-like; V-type proton ATPase subunit a; ATPase, V0/A0 complex subunit a			
**DUP55**	scaffold2|size2959145	2,853,900	2,855,700	1,800	5	MCO448 PL429.5000 PL429.miseq PL448 SZMC8094 SZMC8095	3.05 2.51 2.52 3.67 2.65 2.58		g4393 g4394	Tetratricopeptide-like helical; Sel1-like; Dna2/Cas4, domain of unknown function DUF83; Dna2; DNA replication factor Dna2, N-terminal			
**DUP56**	scaffold2|size2959145	2,856,000	2,857,800	1,800	6	CP367 MCO448 PL429.5000 PL429.miseq PL448 SZMC8094 SZMC8095	2.55 2.96 2.54 2.60 3.81 2.73 2.56		g4394	Dna2/Cas4, domain of unknown function DUF83; Dna2; DNA replication factor Dna2, N-terminal			
**DUP57**	scaffold2|size2959145	2,870,600	2,871,800	1,200	4	MCO448 PL429.5000 PL448 SZMC8094	2.95 2.65 4.09 2.62		g4399	Amino acid permease, conserved site; Amino acid permease domain; Amino acid/polyamine transporter I; Amino acid permease, fungi			
**DUP58**	scaffold2|size2959145	2,874,900	2,878,100	3,200	5	MCO448 PL429.5000 PL429.miseq PL448 SZMC8094 SZMC8095	3.06 2.56 2.51 3.82 2.56 2.51		g4402	WD40/YVTN repeat-like-containing domain; WD40-repeat-containing domain; Soluble quinoprotein glucose/sorbosone dehydrogenase; WD40 repeat, conserved site; WD40 repeat			
**DUP59**	scaffold2|size2959145	2,880,400	2,882,700	2,300	4	MCO448 PL429.5000 PL429.miseq PL448 SZMC8094	2.62 2.51 2.51 3.94 2.53		g4405	Multicopper oxidase, copper-binding site; Multicopper oxidase, type 2; Multicopper oxidase, type 3; Multicopper oxidase, type 1; Cupredoxin			
**DUP60**	scaffold2|size2959145	2,895,300	2,897,900	2,600	3	MCO448 PL448 SZMC8094	2.71 3.47 2.51		g4409 g4410	Ubiquitin activating enzyme, alpha domain; Ubiquitin-activating enzyme repeat; E2 binding; Molybdenum cofactor biosynthesis, MoeB; UBA/THIF-type NAD/FAD binding fold; NAD(P)-binding domain; Ubiquitin-activating enzyme, E1, active site; Ubiquitin-activating enzyme			
**DUP61**	scaffold2|size2959145	2,898,200	2,902,200	4,000	5	MCO448 PL429.5000 PL448 SZMC8094 SZMC8095	3.01 2.50 3.93 2.56 2.51		g4412 g4411	Restriction endonuclease type II--like; ERCC4 domain; Winged helix-turn-helix transcription repressor DNA-binding; DNA repair nuclease, XPF-type/Helicase; Like-Sm (LSM) domain; Rad52/22 double-strand break repair protein; Ribonucleoprotein LSM domain, eukaryotic/archaea-type; Ribonucleoprotein LSM domain			
**DUP62**	scaffold2|size2959145	2,903,800	2,907,800	4,000	2	MCO448 PL448	2.56 3.79		g4414 g4415 g4416	Nucleoporin protein Ndc1-Nup; Cation/H+ exchanger, CPA1 family; Na+/H+ exchanger; Cation/H+ exchanger, CPA1 family; Cation/H+ exchanger			
**DUP63**	scaffold2|size2959145	2,908,200	2,909,500	1,300	2	PL429.5000 PL448	2.53 3.72		g4417	Domain of unknown function DUF250			
**DUP64**	scaffold2|size2959145	2,911,700	2,913,300	1,600	4	MCO448 PL429.5000 PL448 SZMC8094	2.92 2.55 4.05 2.52		g4419	Target SNARE coiled-coil domain			

**DUP65**	scaffold2|size2959145	2,915,900	2,936,400	20,500	8	BP57 CP367 CP61 MCO448 PL429.300 PL429.5000 PL429.600 PL448 SZMC8094 SZMC8095	2.75 2.53 2.76 3.24 2.57 2.79 2.67 4.26 2.91 2.73		g4421 g4422 g4423 g4424 g4425 g4426 g4427 g4428 g4429 g4430 g4431 g4420 g4432	3-oxo-5-alpha-steroid 4-dehydrogenase, C-terminal; Isoprenylcysteine carboxyl methyltransferase; Protein-S-isoprenylcysteine O-methyltransferase; Isoprenylcysteine carboxyl methyltransferase; Protein-S-isoprenylcysteine O-methyltransferase; ARP2/3 complex, p21-Arc subunit; WD40/YVTN repeat-like-containing domain; WD40 repeat, conserved site; WD40-repeat-containing domain; WD40 repeat; Calcipressin; Leucine-rich repeat; Leucine rich repeat 4; Glutathione-dependent formaldehyde-activating enzyme/centromere protein V; Fumarylacetoacetase, C-terminal-related; Fumarylacetoacetase, C-terminal; RmlC-like cupin domain; RmlC-like jelly roll fold; Cupin 2, conserved barrel; Major facilitator superfamily domain, general substrate transporter; Major facilitator superfamily; Major facilitator superfamily domain; Fatty acid desaturase, type 1; Monooxygenase, FAD-binding; Aromatic-ring hydroxylase-like			
**DUP66**	scaffold3|size1388033	269,300	271,800	2,500	8	BP57 CP61 MCO448 PL429.300 PL429.5000 PL429.600 PL429.miseq PL448 SZMC8029 SZMC8092 SZMC8095	3.03 3.07 3.66 2.85 3.52 2.87 3.73 2.89 3.20 3.27 3.71		g1661 g1662	Lipase, secreted; Lipase, secreted			
**DUP67**	scaffold3|size1388033	317,800	319,500	1,700	9	BP57 CP61 MCO448 PL429.300 PL429.5000 PL429.600 PL429.miseq PL448 SZMC21154 SZMC8029 SZMC8092 SZMC8095	3.51 2.82 3.42 3.30 3.22 3.18 3.99 2.91 3.46 3.99 3.81 3.96		g1683	Major facilitator superfamily domain, general substrate transporter			
**DUP68**	scaffold3|size1388033	373,000	375,000	2,000	6	MCO448 PL429.5000 PL448 SZMC8029 SZMC8092 SZMC8095	3.45 2.74 2.97 2.65 2.81 3.01		g1708 g1709				
**DUP69**	scaffold3|size1388033	486,100	488,500	2,400	10	BP57 CP367 MCO448 PL429.300 PL429.5000 PL429.600 PL429.miseq PL448 SZMC21154 SZMC8029 SZMC8092 SZMC8094 SZMC8095	3.08 3.07 3.74 3.60 3.54 3.77 4.19 3.17 3.61 3.17 3.18 3.23 3.50		g1753	PT repeat			
**DUP70**	scaffold3|size1388033	543,100	544,300	1,200	10	CP367 CP61 MCO448 PL429.300 PL429.5000 PL429.600 PL429.miseq PL448 SZMC21154 SZMC8029 SZMC8092 SZMC8094 SZMC8095	3.04 3.71 3.90 2.88 3.28 3.01 3.59 3.10 2.97 3.24 3.23 3.34 3.79		g1774				
**DUP71**	scaffold3|size1388033	1,119,400	1,120,700	1,300	7	BP57 MCO448 PL429.300 PL429.5000 PL429.600 PL429.miseq PL448 SZMC8029 SZMC8092 SZMC8095	2.58 3.79 2.61 2.88 2.52 3.38 2.93 2.65 2.74 3.30		g2036	RNA polymerase II-associated, Paf1			
**DUP72**	scaffold3|size1388033	1,270,300	1,271,900	1,600	11	BP57 CP367 CP61 MCO448 PL429.300 PL429.5000 PL429.600 PL429.miseq PL448 SZMC21154 SZMC8029 SZMC8092 SZMC8094 SZMC8095	2.97 4.18 3.81 4.29 3.45 4.01 3.26 4.13 3.82 3.44 3.63 3.81 5.43 4.37		g2107	Major facilitator superfamily domain, general substrate transporter; Major facilitator superfamily			
**DUP73**	scaffold3|size1388033	1,363,800	1,370,900	7,100	8	BP57 CP61 MCO448 PL448 SZMC21154 SZMC8029 SZMC8092 SZMC8094	2.96 3.41 3.96 3.77 3.12 2.52 2.75 2.90		g2149 g2150 g2151 g2152 g2153	Leucine-rich repeat; Mss4-like; Glutathione-dependent formaldehyde-activating enzyme/centromere protein V; Fumarylacetoacetase, C-terminal-related; Fumarylacetoacetase, C-terminal; RmlC-like cupin domain; RmlC-like jelly roll fold; Cupin 2, conserved barrel; Major facilitator superfamily domain, general substrate transporter; Major facilitator superfamily; Major facilitator superfamily domain			
**DUP74**	scaffold4|size908642	877,600	879,000	1,400	6	CP367 MCO448 PL429.5000 PL429.miseq PL448 SZMC8094 SZMC8095	2.72 3.40 2.76 2.69 3.27 2.74 2.61		g716	ATPase, dynein-related, AAA domain; Midasin; AAA+ ATPase domain; von Willebrand factor, type A; Sigma-54 interaction domain, ATP-binding site 1			
**DUP75**	scaffold4|size908642	891,900	897,900	6,000	8	BP57 MCO448 PL429.300 PL429.5000 PL429.600 PL429.miseq PL448 SZMC21154 SZMC8029 SZMC8092 SZMC8095	3.04 4.49 3.20 3.16 3.28 3.45 5.56 2.74 2.99 3.07 3.48		g722 g723 g724 g725	Fumarylacetoacetase, C-terminal-related; Fumarylacetoacetase, C-terminal; RmlC-like cupin domain; RmlC-like jelly roll fold; Cupin 2, conserved barrel; Major facilitator superfamily domain, general substrate transporter; Major facilitator superfamily; Major facilitator superfamily domain; Monooxygenase, FAD-binding; Aromatic-ring hydroxylase-like			
**DUP76**	scaffold4|size908642	898,500	900,100	1,600	7	BP57 CP61 MCO448 PL429.5000 PL448 SZMC8094 SZMC8095	2.70 4.04 3.77 1.45 4.30 3.20 2.84		g726				
**DUP77**	scaffold5|size1063617	718,562	729,498	10,936	11	BP57 CP367 CP61 MCO448 PL429.300 PL429.5000 PL429.600 PL429.miseq PL448 SZMC21154 SZMC8029 SZMC8092 SZMC8094 SZMC8095	36.18 36.32 38.33 43.87 31.00 50.73 29.39 49.07 34.75 45.36 39.00 38.93 36.79 41.53		g1386 g1387	CheY-like superfamily; Signal transduction response regulator, receiver domain			
**DUP78**	scaffold6|size820601	269,300	270,600	1,300	9	BP57 CP61 MCO448 PL429.300 PL429.5000 PL429.600 PL429.miseq PL448 SZMC21154 SZMC8029 SZMC8092 SZMC8095	3.07 3.07 4.00 2.85 2.99 2.84 4.20 3.04 2.79 2.98 3.17 3.58		g854	Short-chain dehydrogenase/reductase, conserved site; Glucose/ribitol dehydrogenase; Short-chain dehydrogenase/reductase SDR; NAD(P)-binding domain; MaoC-like dehydratase; Polyketide synthase/Fatty acid synthase, KR			
**DUP79**	scaffold6|size820601	323,373	336,324	12,951	2	MCO448 PL448	2.66 3.30		g872 g873 g875 g876 g877 g874	Immunoglobulin E-set; Vacuolar protein sorting-associated protein 26; HAD-superfamily phosphatase, subfamily IIIC; HAD-like domain; Magnesium-dependent phosphatase-1, eukaryotic type; Magnesium-dependent phosphatase-1, eukaryotic/arcaheal type; Zinc finger, ZZ-type; Ras guanine-nucleotide exchange factor, conserved site; Ras guanine nucleotide exchange factor, domain; Src homology-3 domain; Ras guanine nucleotide exchange factor; Ras-like guanine nucleotide exchange factor, N-terminal; Guanine-nucleotide dissociation stimulator CDC25			x
**DUP80**	scaffold6|size820601	538,800	545,100	6,300	6	MCO448 PL429.300 PL429.5000 PL429.600 PL429.miseq SZMC21154 SZMC8029 SZMC8092 SZMC8095	3.42 3.00 3.68 3.27 3.37 2.85 3.04 3.13 3.31		g953 g954 g955 g956	Exo70 exocyst complex subunit; Cullin repeat-like-containing domain; tRNA ligase, phosphodiesterase, fungi; tRNA ligase, fungi; RNA ligase, T4 RnlA-like; tRNA ligase, kinase, fungi; Glutaredoxin; Thioredoxin-like fold; Glutaredoxin active site; Glutaredoxin subgroup; Glutaredoxin, eukaryotic/virial; Folliculin			

**DUP81**	scaffold7|size2161850	72,600	73,892	1,292	6	BP57 CP367 CP61 PL429.300 PL429.5000 PL429.600 PL429.miseq SZMC21154 SZMC8092	4.16 2.78 4.08 4.69 4.61 4.71 3.44 3.62 3.04		.				
**DUP82**	scaffold7|size2161850	156,738	158,628	1,890	8	BP57 CP367 MCO448 PL429.300 SZMC8029 SZMC8092 SZMC8094 SZMC8095	2.72 2.53 2.84 1.45 2.54 2.66 3.04 2.50		g2225 g2226	Zinc finger, RING-type; Zinc finger, RING/FYVE/PHD-type; Zinc finger, CCCH-type			
**DUP83**	scaffold7|size2161850	1,529,000	1,530,300	1,300	10	CP367 CP61 MCO448 PL429.5000 PL429.miseq PL448 SZMC21154 SZMC8029 SZMC8092 SZMC8094 SZMC8095	3.28 3.11 3.89 2.54 2.56 3.02 2.88 3.17 3.25 3.34 3.66		g2874	Enolase, C-terminal; Enolase, N-terminal; Enolase; Enolase, conserved site			
**DUP84**	scaffold7|size2161850	1,747,200	1,756,800	9,600	6	BP57 CP367 CP61 MCO448 PL429.5000 PL429.600 PL429.miseq PL448	5.42 2.52 6.31 2.58 2.83 2.52 2.67 2.71		g2959	Globin; Riboflavin synthase-like beta-barrel; Globin, structural domain; Globin-like			x
**DUP85**	scaffold7|size2161850	1,761,400	1,763,100	1,700	8	BP57 CP367 CP61 PL429.300 PL429.5000 PL429.600 PL448 SZMC21154 SZMC8029 SZMC8092	3.92 3.98 3.55 2.79 2.99 3.59 4.18 2.81 2.92 3.29		g2963				
**DUP86**	scaffold8|size317314	193,500	195,400	1,900	11	BP57 CP367 CP61 MCO448 PL429.300 PL429.5000 PL429.600 PL429.miseq PL448 SZMC21154 SZMC8029 SZMC8092 SZMC8094 SZMC8095	4.63 3.19 4.91 5.81 5.69 6.86 5.90 6.47 5.20 6.51 5.79 6.14 3.67 6.92		g278	DDE superfamily endonuclease, CENP-B-like			
**DUP87**	scaffold8|size317314	277,300	279,200	1,900	11	BP57 CP367 CP61 MCO448 PL429.300 PL429.5000 PL429.600 PL429.miseq PL448 SZMC21154 SZMC8029 SZMC8092 SZMC8094 SZMC8095	4.37 3.18 4.70 5.82 5.17 7.76 6.12 4.65 5.40 6.06 5.26 5.91 3.94 6.60		g308	DDE superfamily endonuclease, CENP-B-like			
**DEL1**	scaffold1|size3459122	113,330	119,716	6,386	7	BP57 CP367 CP61 PL429.5000 PL448 SZMC21154 SZMC8094	0.78 1.33 1.48 1.36 1.42 1.47 1.11	no	g4475 g4476 g4477	Epoxide hydrolase-like			
**DEL2**	scaffold1|size3459122	434,200	436,100	1,900	4	BP57 CP367 CP61 PL429.300 PL429.5000 PL429.600 PL429.miseq	0.24 1.05 1.02 1.21 1.37 1.24 1.19	no	g4632				
**DEL3**	scaffold1|size3459122	824,955	827,915	2,960	11	BP57 CP367 CP61 MCO448 PL429.300 PL429.5000 PL429.600 PL429.miseq PL448 SZMC21154 SZMC8029 SZMC8092 SZMC8094 SZMC8095	1.43 1.29 1.38 1.22 1.23 1.44 1.33 1.39 1.17 1.22 1.34 1.33 1.32 1.31	no	g4805	ABC transporter-like; AAA+ ATPase domain; ABC transporter, N-terminal; ABC transporter, transmembrane domain, type 1			
**DEL4**	scaffold1|size3459122	985,207	988,069	2,862	9	BP57 CP367 CP61 PL429.300 PL429.5000 PL429.600 SZMC21154 SZMC8029 SZMC8092 SZMC8094 SZMC8095	1.26 1.37 1.32 1.27 1.43 1.34 1.17 1.28 1.33 1.37 1.49	no	g4867	Major facilitator superfamily domain, general substrate transporter; Major facilitator superfamily			
**DEL5**	scaffold1|size3459122	990,300	991,600	1,300	11	BP57 CP367 CP61 MCO448 PL429.5000 PL429.600 PL429.miseq PL448 SZMC21154 SZMC8029 SZMC8092 SZMC8094 SZMC8095	0.91 0.44 0.64 0.14 0.83 0.94 0.43 0.21 0.98 0.93 0.53 0.50 0.59	no	.				
**DEL6**	scaffold1|size3459122	996,386	1,007,717	11,331	9	CP367 MCO448 PL429.miseq PL448 SZMC21154 SZMC8029 SZMC8092 SZMC8094 SZMC8095	1.32 1.23 1.41 1.43 1.41 1.47 1.43 1.08 1.27	no	g4870 g4872 g4873 g4871	GPR1/FUN34/yaaH; GPR1/FUN34/yaaH			
**DEL7**	scaffold1|size3459122	1,716,100	1,717,300	1,200	6	MCO448 PL429.300 PL429.600 PL429.miseq PL448 SZMC8092 SZMC8094 SZMC8095	0.14 2.89 2.56 1.25 0.37 1.41 0.94 0.76	no	g5203				
**DEL8**	scaffold1|size3459122	1,729,086	1,743,062	13,976	11	BP57 CP367 CP61 MCO448 PL429.300 PL429.5000 PL429.600 PL429.miseq PL448 SZMC21154 SZMC8029 SZMC8092 SZMC8094 SZMC8095	1.12 1.17 1.04 1.22 1.10 1.25 1.04 1.02 1.09 0.96 1.04 1.08 0.96 1.07	no	g5207 g5208 g5209 g5210 g5211 g5212 g5214 g5215 g5216 g5213	ATPase inhibitor, IATP, mitochondria; Spo11/DNA topoisomerase VI, subunit A			
**DEL9**	scaffold1|size3459122	1,875,382	1,878,239	2,857	9	BP57 CP367 MCO448 PL429.miseq PL448 SZMC21154 SZMC8029 SZMC8094 SZMC8095	1.46 1.41 0.99 1.45 1.23 1.33 1.48 1.19 1.15	no	g5273 g5274 g5275	Glutaredoxin; Thioredoxin-like fold; Glutaredoxin active site; Glutaredoxin subgroup; Glutaredoxin, eukaryotic/virial			
**DEL10**	scaffold1|size3459122	1,889,249	1,890,676	1,427	11	BP57 CP367 CP61 MCO448 PL429.300 PL429.5000 PL429.600 PL429.miseq PL448 SZMC21154 SZMC8029 SZMC8092 SZMC8094 SZMC8095	1.15 1.03 1.15 1.30 1.00 1.29 1.05 1.22 1.05 1.10 1.22 1.07 1.17 1.12	no	g5282				
**DEL11**	scaffold1|size3459122	2,110,585	2,111,810	1,225	11	BP57 CP367 CP61 MCO448 PL429.300 PL429.5000 PL429.600 PL429.miseq PL448 SZMC21154 SZMC8029 SZMC8092 SZMC8094 SZMC8095	1.24 0.97 1.14 0.51 1.32 1.23 1.17 0.84 0.59 0.94 1.01 0.70 0.61 0.70	no	g5396	CinA, C-terminal			
**DEL12**	scaffold1|size3459122	2,781,300	2,789,500	8,200	7	CP367 MCO448 PL448 SZMC21154 SZMC8029 SZMC8092 SZMC8095	2.70 0.22 0.20 1.03 1.17 0.11 1.32	yes	g5707 g5708	Hyphally-regulated cell wall protein, N-terminal; Hyphally-regulated cell wall protein, N-terminal			
**DEL13**	scaffold1|size3459122	2,883,492	2,890,688	7,196	11	BP57 CP367 CP61 MCO448 PL429.300 PL429.5000 PL429.600 PL429.miseq PL448 SZMC21154 SZMC8029 SZMC8092 SZMC8094 SZMC8095	1.15 1.08 1.11 0.87 1.08 1.26 1.11 0.99 0.92 1.02 1.11 1.08 0.92 0.86	no	g5741	ABC transporter-like; ABC transporter, conserved site; CDR ABC transporter; AAA+ ATPase domain; ABC-2 type transporter; Pleiotropic drug resistance protein PDR			

**DEL14**	scaffold1|size3459122	3,113,072	3,124,600	11,528	8	BP57 MCO448 PL429.300 PL429.5000 PL429.600 PL429.miseq PL448 SZMC21154 SZMC8029 SZMC8092 SZMC8095	1.34 0.53 0.54 0.76 0.59 0.54 0.48 0.49 0.52 0.52 0.47	no	g5831 g5832 g5833 g5834	Acyl-CoA oxidase/dehydrogenase, central domain; Acyl-CoA oxidase; Acyl-CoA dehydrogenase/oxidase; Acyl-CoA oxidase/dehydrogenase, type 1; Acyl-CoA oxidase, C-terminal; Acyl-CoA dehydrogenase/oxidase, N-terminal; Acyl-CoA dehydrogenase/oxidase C-terminal; Acyl-CoA oxidase/dehydrogenase, central domain; Acyl-CoA oxidase; Acyl-CoA oxidase/dehydrogenase, type 1; Acyl-CoA oxidase, C-terminal; Acyl-CoA dehydrogenase/oxidase; Acyl-CoA dehydrogenase/oxidase, N-terminal; Acyl-CoA dehydrogenase/oxidase C-terminal; Acyl-CoA oxidase/dehydrogenase, central domain; Acyl-CoA oxidase; Acyl-CoA oxidase/dehydrogenase, type 1; Acyl-CoA oxidase, C-terminal; Acyl-CoA dehydrogenase/oxidase; Acyl-CoA dehydrogenase/oxidase, N-terminal; Acyl-CoA dehydrogenase/oxidase C-terminal; Acyl-CoA oxidase/dehydrogenase, central domain; Acyl-CoA oxidase; Acyl-CoA oxidase, C-terminal; Acyl-CoA dehydrogenase/oxidase; Acyl-CoA dehydrogenase/oxidase, N-terminal; Acyl-CoA dehydrogenase/oxidase C-terminal			x
**DEL15**	scaffold1|size3459122	3,288,261	3,289,501	1,240	11	BP57 CP367 CP61 MCO448 PL429.600 PL448 SZMC21154 SZMC8029 SZMC8092 SZMC8094 SZMC8095	1.16 1.14 0.45 0.74 2.51 0.94 1.12 1.20 1.24 1.07 0.93	no	g5913 g5914				
**DEL16**	scaffold2|size2959145	318,193	321,320	3,127	8	CP367 MCO448 PL429.300 PL429.5000 PL429.600 PL429.miseq PL448 SZMC8029 SZMC8092 SZMC8094 SZMC8095	1.34 0.13 1.15 0.88 0.92 1.02 0.43 1.22 1.18 0.68 0.41	yes	.				
**DEL17**	scaffold2|size2959145	551,073	554,244	3,171	10	BP57 CP367 CP61 MCO448 PL429.300 PL429.5000 PL429.600 PL429.miseq PL448 SZMC8029 SZMC8092 SZMC8094 SZMC8095	1.28 1.22 1.27 1.36 1.25 1.46 1.32 1.30 1.15 1.33 1.31 1.32 1.39	no	g3413				
**DEL18**	scaffold2|size2959145	709,317	712,224	2,907	10	BP57 CP367 CP61 MCO448 PL429.300 PL429.5000 PL429.600 PL429.miseq PL448 SZMC8029 SZMC8092 SZMC8094 SZMC8095	0.91 1.49 1.38 1.06 0.82 0.71 0.79 0.86 1.34 1.47 1.44 1.15 1.14	no	g3480	Amino acid permease, conserved site; Amino acid permease domain; Amino acid/polyamine transporter I			
**DEL19**	scaffold2|size2959145	868,587	870,482	1,895	11	BP57 CP367 CP61 MCO448 PL429.300 PL429.5000 PL429.600 PL429.miseq PL448 SZMC21154 SZMC8029 SZMC8092 SZMC8094 SZMC8095	1.09 1.02 1.02 1.01 1.12 1.27 1.12 1.17 0.87 0.96 1.09 1.14 1.01 1.04	no	g3551				
**DEL20**	scaffold2|size2959145	898,817	901,840	3,023	9	BP57 CP367 CP61 PL429.300 PL429.600 PL429.miseq PL448 SZMC8029 SZMC8092 SZMC8094 SZMC8095	1.20 1.23 1.24 1.23 1.20 1.23 1.31 1.18 1.23 1.30 1.46	no	g3562 g3563	Alcohol dehydrogenase, long-chain fatty; Glucose-methanol-choline oxidoreductase, N-terminal; Glucose-methanol-choline oxidoreductase, C-terminal; Alcohol dehydrogenase, long-chain fatty; Glucose-methanol-choline oxidoreductase, N-terminal; Glucose-methanol-choline oxidoreductase, C-terminal			
**DEL21**	scaffold2|size2959145	1,236,500	1,244,200	7,700	11	BP57 CP367 CP61 MCO448 PL429.300 PL429.5000 PL429.600 PL429.miseq PL448 SZMC21154 SZMC8029 SZMC8092 SZMC8094 SZMC8095	1.01 0.88 0.37 0.42 0.20 0.14 0.17 0.23 0.78 1.09 1.19 1.10 0.73 0.64	yes	g3719 g3720				
**DEL22**	scaffold2|size2959145	1,445,328	1,450,311	4,983	9	BP57 CP61 MCO448 PL429.300 PL429.5000 PL429.600 PL429.miseq PL448 SZMC21154 SZMC8029 SZMC8092 SZMC8095	1.32 1.33 1.24 1.19 1.47 1.29 1.34 1.13 1.29 1.21 1.29 1.16	no	g3802 g3803 g3804	GPR1/FUN34/yaaH; Oligopeptide transporter OPT superfamily; Tetrapeptide transporter, OPT1/isp4; Coatomer, epsilon subunit			
**DEL23**	scaffold2|size2959145	1,498,800	1,509,000	10,200	4	CP367 PL429.300 PL429.600 SZMC21154 SZMC8094	0.16 1.27 1.31 2.55 1.05	yes	g3822 g3823 g3824 g3825	Flocculin type 3 repeat; Flocculin type 3 repeat; Flocculin type 3 repeat; Cytadhesin P30P32; Flocculin type 3 repeat			x
**DEL24**	scaffold2|size2959145	1,675,200	1,678,100	2,900	7	CP367 MCO448 PL429.miseq PL448 SZMC21154 SZMC8094 SZMC8095	1.47 0.27 1.32 0.98 2.60 0.87 0.72	no	g3901	Glutamate/phenylalanine/leucine/valine dehydrogenase, dimerisation domain; Glutamate/phenylalanine/leucine/valine dehydrogenase; NAD(P)-binding domain; Glutamate/phenylalanine/leucine/valine dehydrogenase, C-terminal; Glutamate dehydrogenase			
**DEL25**	scaffold2|size2959145	1,791,300	1,795,700	4,400	11	BP57 CP367 CP61 MCO448 PL429.300 PL429.5000 PL429.600 PL429.miseq PL448 SZMC21154 SZMC8029 SZMC8092 SZMC8094 SZMC8095	0.50 1.28 1.13 0.08 1.24 1.35 1.28 1.28 0.09 0.32 1.21 1.20 1.12 1.09	yes	g3952 g3953	Microsomal signal peptidase 12kDa subunit			
**DEL26**	scaffold2|size2959145	1,810,131	1,814,400	4,269	8	CP367 CP61 PL429.300 PL429.5000 PL429.600 PL429.miseq SZMC21154 SZMC8029 SZMC8092 SZMC8094 SZMC8095	1.23 1.40 1.24 1.49 1.23 1.41 2.93 1.24 1.26 1.26 0.17	yes	g3961 g3962	NAD(P)-binding domain; Bicarbonate transporter, C-terminal; Glucose/ribitol dehydrogenase; Bicarbonate transporter, eukaryotic; NAD(P)-binding domain; Glucose/ribitol dehydrogenase			
**DEL27**	scaffold2|size2959145	1,919,880	1,928,240	8,360	7	CP367 CP61 PL429.300 PL429.5000 PL429.600 PL429.miseq SZMC21154 SZMC8029 SZMC8092 SZMC8094	1.23 1.46 0.34 0.36 0.35 0.39 2.95 1.24 1.28 1.13	no	g4002 g4003 g4004 g4005	Proteinase inhibitor, propeptide; Proteinase inhibitor I9			
**DEL28**	scaffold2|size2959145	2,512,055	2,513,269	1,214	10	BP57 CP367 CP61 MCO448 PL429.300 PL429.5000 PL429.600 PL429.miseq SZMC21154 SZMC8029 SZMC8092 SZMC8094 SZMC8095	1.29 1.00 1.21 1.10 1.05 1.22 1.09 1.13 1.17 1.22 1.26 1.32 1.34	no	.				
**DEL29**	scaffold2|size2959145	2,743,374	2,748,383	5,009	9	BP57 CP61 MCO448 PL429.300 PL429.600 PL429.miseq PL448 SZMC21154 SZMC8029 SZMC8092 SZMC8095	0.59 1.21 1.41 1.22 1.30 1.15 1.47 1.10 1.20 1.24 1.28	no	g4347 g4348 g4349				
**DEL30**	scaffold2|size2959145	2,814,593	2,816,446	1,853	7	BP57 CP61 MCO448 SZMC21154 SZMC8029 SZMC8092 SZMC8095	1.35 1.25 0.75 1.21 1.29 1.13 0.90	no	.				

**DEL31**	scaffold2|size2959145	2,845,748	2,848,612	2,864	10	CP367 CP61 MCO448 PL429.5000 PL429.600 PL429.miseq PL448 SZMC21154 SZMC8029 SZMC8092 SZMC8094 SZMC8095	1.05 1.32 0.63 1.29 1.11 0.86 1.15 1.18 1.29 1.13 1.14 0.88	no	.				
**DEL32**	scaffold2|size2959145	2,892,059	2,895,227	3,168	11	BP57 CP367 CP61 MCO448 PL429.300 PL429.5000 PL429.600 PL429.miseq PL448 SZMC21154 SZMC8029 SZMC8092 SZMC8094 SZMC8095	1.44 1.32 1.48 1.39 1.46 1.46 1.48 1.41 1.41 1.36 1.43 1.47 1.36 1.36	no	g4408	AMP-dependent synthetase/ligase			
**DEL33**	scaffold3|size1388033	17,480	19,007	1,527	5	CP367 CP61 PL429.5000 PL429.600 PL448 SZMC8094	1.19 1.44 2.71 2.66 2.93 1.32	no	g1546				
**DEL34**	scaffold3|size1388033	102,152	105,897	3,745	11	BP57 CP367 CP61 MCO448 PL429.300 PL429.5000 PL429.600 PL429.miseq PL448 SZMC21154 SZMC8029 SZMC8092 SZMC8094 SZMC8095	1.36 1.36 1.37 0.42 0.46 0.36 0.35 0.57 0.35 0.55 0.55 0.58 1.49 0.65	no	g1583 g1584	Leucine-rich repeat; Leucine-rich repeat; Leucine rich repeat 4			
**DEL35**	scaffold3|size1388033	106,700	109,400	2,700	3	MCO448 PL448 SZMC8095	0.57 1.14 1.25	no	g1586	Alpha-mannosyltransferase			
**DEL36**	scaffold3|size1388033	113,686	115,244	1,558	8	BP57 CP367 CP61 PL429.300 PL429.5000 PL429.600 PL448 SZMC8029 SZMC8092 SZMC8094	1.26 1.26 1.23 1.19 1.41 1.10 1.23 1.21 1.29 1.49	no	g1588	Bromodomain			
**DEL37**	scaffold3|size1388033	165,330	168,490	3,160	8	CP367 MCO448 PL429.300 PL429.5000 PL429.600 PL429.miseq PL448 SZMC21154 SZMC8029 SZMC8092 SZMC8095	1.32 1.24 1.37 1.39 1.39 1.16 1.09 0.55 1.39 1.39 1.44	no	g1614 g1615				
**DEL38**	scaffold3|size1388033	203,417	207,945	4,528	9	BP57 CP367 CP61 PL429.300 PL429.5000 PL429.600 PL429.miseq PL448 SZMC21154 SZMC8029 SZMC8092 SZMC8094	1.33 1.47 1.26 0.62 0.93 0.60 0.79 1.48 0.63 0.65 1.34 1.47	no	g1631 g1632	Domain of unknown function DUF1212; Domain of unknown function DUF3815; Domain of unknown function DUF1212; Domain of unknown function DUF3815			
**DEL39**	scaffold3|size1388033	234,030	235,403	1,373	10	BP57 CP367 CP61 MCO448 PL429.300 PL429.5000 PL429.600 PL429.miseq PL448 SZMC8029 SZMC8092 SZMC8094 SZMC8095	1.19 1.05 1.21 1.39 1.09 1.30 1.19 1.46 1.01 1.17 1.21 1.34 1.39	no	g1645	Aldolase-type TIM barrel; 2-nitropropane dioxygenase, NPD			
**DEL40**	scaffold3|size1388033	242,232	245,200	2,968	8	BP57 CP61 MCO448 PL429.300 PL429.600 PL448 SZMC8029 SZMC8092 SZMC8095	1.34 1.32 1.37 1.24 1.16 1.11 1.32 1.31 1.36	no	g1650 g1651	GPR1/FUN34/yaaH			
**DEL41**	scaffold3|size1388033	248,590	250,960	2,370	10	BP57 CP367 CP61 MCO448 PL429.300 PL429.5000 PL429.600 PL429.miseq PL448 SZMC8029 SZMC8092 SZMC8094 SZMC8095	1.15 1.07 1.22 1.17 1.12 1.24 1.17 1.10 1.03 1.14 1.16 1.19 1.20	no	g1653	GPR1/FUN34/yaaH			
**DEL42**	scaffold3|size1388033	319,608	320,754	1,146	10	BP57 CP367 CP61 MCO448 PL429.300 PL429.600 PL429.miseq PL448 SZMC8029 SZMC8092 SZMC8094 SZMC8095	1.38 1.24 1.30 0.75 1.30 1.37 1.30 0.89 1.32 1.37 0.97 0.96	no	g1684	Major facilitator superfamily domain, general substrate transporter			
**DEL43**	scaffold3|size1388033	365,541	370,487	4,946	11	BP57 CP367 CP61 MCO448 PL429.300 PL429.5000 PL429.600 PL429.miseq PL448 SZMC21154 SZMC8029 SZMC8092 SZMC8094 SZMC8095	1.21 1.18 1.22 0.01 1.16 1.35 1.22 1.23 0.12 0.20 0.22 0.23 0.91 0.09	yes	g1706 g1707	Peptidase aspartic, catalytic; Peptidase aspartic; Peptidase A1; Peptidase aspartic, catalytic; Peptidase aspartic; Peptidase A1			
**DEL44**	scaffold3|size1388033	394,000	399,100	5,100	10	BP57 CP367 CP61 PL429.300 PL429.600 PL448 SZMC21154 SZMC8029 SZMC8092 SZMC8094 SZMC8095	1.45 1.43 1.41 1.41 1.41 1.31 0.46 1.35 1.37 1.43 1.48	no	g1717				x
**DEL45**	scaffold3|size1388033	434,300	436,600	2,300	8	BP57 CP367 CP61 PL429.300 PL429.600 PL429.miseq SZMC21154 SZMC8029 SZMC8092 SZMC8094	1.23 1.24 1.35 1.18 1.33 1.42 0.36 1.35 1.44 1.50	no	g1737				
**DEL46**	scaffold3|size1388033	551,762	554,533	2,771	11	BP57 CP367 CP61 MCO448 PL429.300 PL429.5000 PL429.600 PL429.miseq PL448 SZMC21154 SZMC8029 SZMC8092 SZMC8094 SZMC8095	1.30 1.10 1.20 1.13 1.27 1.35 0.92 1.33 0.95 1.18 1.21 1.25 1.27 1.39	no	.				
**DEL47**	scaffold3|size1388033	615,500	616,800	1,300	7	CP367 MCO448 PL429.miseq PL448 SZMC8092 SZMC8094 SZMC8095	1.37 0.22 1.03 0.47 0.98 0.92 0.85	no	.				
**DEL48**	scaffold3|size1388033	645,434	648,601	3,167	2	PL429.600 SZMC8095	1.49 2.76	no	g1821				
**DEL49**	scaffold3|size1388033	1,353,815	1,355,838	2,023	10	BP57 CP367 CP61 PL429.300 PL429.5000 PL429.600 PL429.miseq PL448 SZMC21154 SZMC8029 SZMC8092 SZMC8094 SZMC8095	1.13 1.17 1.12 1.06 1.37 1.09 1.39 1.49 1.06 1.09 1.20 1.44 1.34	no	g2146	Peptidase S10, serine carboxypeptidase, active site; Propeptide, carboxypeptidase Y; Peptidase S10, serine carboxypeptidase			
**DEL50**	scaffold4|size908642	152,735	154,008	1,273	11	BP57 CP367 CP61 MCO448 PL429.300 PL429.5000 PL429.600 PL429.miseq PL448 SZMC21154 SZMC8029 SZMC8092 SZMC8094 SZMC8095	1.06 1.05 1.09 0.89 1.07 1.39 1.08 1.20 0.94 0.89 1.03 1.02 0.89 0.84	no	.				
**DEL51**	scaffold4|size908642	327,123	328,611	1,488	11	BP57 CP367 CP61 MCO448 PL429.300 PL429.5000 PL429.600 PL429.miseq PL448 SZMC21154 SZMC8029 SZMC8092 SZMC8094 SZMC8095	1.22 1.12 1.18 1.29 1.15 1.30 1.12 1.29 1.04 1.08 1.22 1.21 1.29 1.30	no	g472				
**DEL52**	scaffold4|size908642	426,382	429,722	3,340	1	PL429.300 PL429.5000 PL429.600 PL429.miseq	1.32 1.49 1.23 1.45	no	g517 g518	Major facilitator superfamily domain, general substrate transporter; General substrate transporter; Major facilitator superfamily domain; Major facilitator superfamily domain, general substrate transporter; General substrate transporter; Major facilitator superfamily domain			

**DEL53**	scaffold4|size908642	489,759	493,368	3,609	11	BP57 CP367 CP61 MCO448 PL429.300 PL429.600 PL429.miseq PL448 SZMC21154 SZMC8029 SZMC8092 SZMC8094 SZMC8095	1.36 1.29 1.34 1.35 1.39 1.43 1.19 1.29 1.35 1.47 1.48 1.05 1.25	no	g550 g551	Ferredoxin reductase-type FAD-binding domain; Riboflavin synthase-like beta-barrel; Ferric reductase transmembrane component-like domain; FAD-binding 8; Ferric reductase, NAD binding; Ferredoxin reductase-type FAD-binding domain; Riboflavin synthase-like beta-barrel; Ferric reductase transmembrane component-like domain; FAD-binding 8; Ferric reductase, NAD binding			
**DEL54**	scaffold4|size908642	517,174	518,284	1,110	9	BP57 CP367 CP61 MCO448 PL429.300 PL429.5000 PL429.600 PL429.miseq PL448 SZMC8092 SZMC8094 SZMC8095	1.34 1.34 1.26 0.85 0.22 0.13 0.13 0.37 1.03 1.26 1.19 1.00	yes	.				
**DEL55**	scaffold4|size908642	545,000	548,100	3,100	10	BP57 CP61 MCO448 PL429.300 PL429.5000 PL429.600 PL429.miseq PL448 SZMC21154 SZMC8029 SZMC8092 SZMC8094 SZMC8095	1.29 1.29 0.50 1.22 1.21 1.32 0.94 0.66 1.07 1.13 1.14 1.20 0.70	no	.				
**DEL56**	scaffold4|size908642	770,651	772,391	1,740	10	CP367 CP61 MCO448 PL429.300 PL429.5000 PL429.600 PL429.miseq PL448 SZMC21154 SZMC8029 SZMC8092 SZMC8094 SZMC8095	1.06 0.81 1.39 0.97 1.33 0.92 1.30 1.18 1.04 0.97 1.12 1.24 1.14	no	g669 g670				
**DEL57**	scaffold4|size908642	843,400	844,900	1,500	9	BP57 CP61 MCO448 PL429.300 PL429.5000 PL429.600 PL429.miseq PL448 SZMC21154 SZMC8029 SZMC8092 SZMC8095	1.41 1.42 0.33 0.54 0.40 0.34 0.79 0.28 0.79 0.76 0.83 0.91	no	.				
**DEL58**	scaffold5|size1063617	213,869	216,916	3,047	9	BP57 CP367 CP61 MCO448 PL429.300 PL429.5000 PL429.600 PL429.miseq SZMC8029 SZMC8092 SZMC8094 SZMC8095	1.06 0.92 1.09 0.86 1.01 1.14 1.07 1.04 1.01 1.09 0.99 0.94	no	g1156 g1157	Poly(A) polymerase; Poly(A) polymerase, RNA-binding domain; Nucleotidyltransferase, class I, C-terminal-like; Poly(A) polymerase, central domain; Nucleotidyl transferase domain; Mating-type protein MAT alpha 1; Alpha box			
**DEL59**	scaffold5|size1063617	218,719	226,776	8,057	11	BP57 CP367 CP61 MCO448 PL429.300 PL429.5000 PL429.600 PL429.miseq PL448 SZMC21154 SZMC8029 SZMC8092 SZMC8094 SZMC8095	0.31 0.33 0.32 0.41 0.32 0.44 0.30 0.37 0.47 0.28 0.31 0.32 0.37 0.38	no	g1158 g1159	Phosphatidylinositol 4-kinase; Phosphatidylinositol Kinase; Phosphatidylinositol 3-/4-kinase, catalytic domain; Protein kinase-like domain; Phosphatidylinositol 3/4-kinase, conserved site; Phosphatidylinositol 4-kinase, Pik1, fungi; Phosphatidylinositol 4-kinase; Phosphatidylinositol Kinase; Phosphatidylinositol 3-/4-kinase, catalytic domain; Protein kinase-like domain; Phosphatidylinositol 3/4-kinase, conserved site; Phosphatidylinositol 4-kinase, Pik1, fungi			x
**DEL60**	scaffold5|size1063617	393,140	396,154	3,014	11	BP57 CP367 CP61 MCO448 PL429.300 PL429.600 PL448 SZMC21154 SZMC8029 SZMC8092 SZMC8094 SZMC8095	1.38 1.36 1.38 1.34 1.26 1.35 1.39 1.26 0.68 1.39 1.48 1.39	no	g1241 g1242 g1240	Steryl acetyl hydrolase; Steryl acetyl hydrolase; Steryl acetyl hydrolase; Lipase, GDXG, active site			
**DEL61**	scaffold5|size1063617	454,240	457,163	2,923	7	CP367 MCO448 PL429.300 PL429.miseq SZMC21154 SZMC8029 SZMC8092 SZMC8094	1.48 1.19 1.39 1.48 1.33 1.39 1.43 1.22	no	g1263 g1264	Mediator complex, subunit Med14			
**DEL62**	scaffold6|size820601	116,984	122,149	5,165	10	BP57 CP367 CP61 MCO448 PL429.300 PL429.5000 PL429.600 PL429.miseq SZMC21154 SZMC8029 SZMC8092 SZMC8094 SZMC8095	0.40 1.19 1.15 1.12 0.18 0.19 0.18 0.17 1.05 1.14 1.17 0.63 1.07	yes	g781 g782 g783	Zinc finger, CCCH-type; F-box domain, cyclin-like			x
**DEL63**	scaffold6|size820601	133,496	136,635	3,139	7	BP57 MCO448 PL429.300 PL429.5000 PL429.600 PL429.miseq SZMC21154 SZMC8029 SZMC8092 SZMC8095	1.24 1.25 1.16 1.23 0.94 1.11 1.09 1.18 1.20 1.29	no	g789 g790	Zn(2)-C6 fungal-type DNA-binding domain			
**DEL64**	scaffold6|size820601	150,530	152,258	1,728	9	BP57 CP367 CP61 MCO448 PL429.300 PL429.5000 PL429.600 PL429.miseq SZMC21154 SZMC8029 SZMC8092 SZMC8095	1.03 1.16 1.07 2.98 1.03 1.29 1.03 1.46 1.00 1.07 1.11 1.35	no	g797				
**DEL65**	scaffold6|size820601	169,513	173,531	4,018	9	BP57 CP367 CP61 MCO448 PL429.300 PL429.5000 PL429.600 PL429.miseq SZMC21154 SZMC8029 SZMC8092 SZMC8095	1.36 1.26 1.37 0.66 1.36 1.37 1.38 1.24 1.22 1.31 1.29 0.84	no	g808				
**DEL66**	scaffold6|size820601	323,769	324,914	1,145	11	BP57 CP367 CP61 MCO448 PL429.300 PL429.5000 PL429.600 PL429.miseq PL448 SZMC21154 SZMC8029 SZMC8092 SZMC8094 SZMC8095	1.31 1.17 1.17 1.21 1.21 1.26 1.18 1.48 0.13 1.08 1.21 1.21 1.21 1.28	yes	g872 g873	Immunoglobulin E-set; Vacuolar protein sorting-associated protein 26			
**DEL67**	scaffold6|size820601	676,095	680,540	4,445	9	BP57 CP61 MCO448 PL429.300 PL429.5000 PL429.600 PL429.miseq PL448 SZMC21154 SZMC8029 SZMC8092 SZMC8095	1.33 1.32 0.67 0.55 0.58 0.43 0.84 0.54 0.63 0.71 0.70 0.82	no	g1006 g1007 g1008	Major facilitator superfamily domain, general substrate transporter; Major facilitator superfamily; Major facilitator superfamily domain; Major facilitator superfamily domain, general substrate transporter; Major facilitator superfamily; Major facilitator superfamily domain; Conserved hypothetical protein CHP00092; Beta-grasp domain; GTP binding domain; TGS-like; TGS-like domain; Domain of unknown function DUF933			
**DEL68**	scaffold7|size2161850	73,828	76,442	2,614	10	BP57 CP367 CP61 MCO448 PL429.300 PL448 SZMC21154 SZMC8092 SZMC8094 SZMC8095	0.92 1.04 1.43 1.07 1.46 1.22 1.37 1.10 1.17 1.04	no	g2184 g2185	FMN-binding split barrel-related; FMN-binding split barrel; Oxoglutarate/iron-dependent dioxygenase			
**DEL69**	scaffold7|size2161850	108,069	114,600	6,531	11	BP57 CP367 CP61 MCO448 PL429.300 PL429.5000 PL429.600 PL429.miseq PL448 SZMC21154 SZMC8029 SZMC8092 SZMC8094 SZMC8095	1.25 1.29 0.79 0.23 0.84 0.62 0.77 0.64 0.51 0.67 0.74 0.64 1.08 0.39	no	g2199 g2200 g2201 g2202	F-box domain, cyclin-like; Steryl acetyl hydrolase			x
**DEL70**	scaffold7|size2161850	132,000	133,400	1,400	5	MCO448 PL429.miseq PL448 SZMC8094 SZMC8095	0.59 1.33 0.92 1.29 1.30	no	.				
**DEL71**	scaffold7|size2161850	383,929	385,960	2,031	11	BP57 CP367 CP61 MCO448 PL429.300 PL429.5000 PL429.600 PL429.miseq PL448 SZMC21154 SZMC8029 SZMC8092 SZMC8094 SZMC8095	1.20 1.17 1.17 1.28 1.19 1.40 1.12 1.43 1.04 0.99 1.08 1.09 1.17 1.24	no	g2341	Amino acid/polyamine transporter I			

**DEL72**	scaffold7|size2161850	619,286	620,929	1,643	11	BP57 CP367 CP61 MCO448 PL429.300 PL429.5000 PL429.600 PL429.miseq PL448 SZMC21154 SZMC8029 SZMC8092 SZMC8094 SZMC8095	1.36 1.20 1.42 1.18 1.20 1.44 1.33 1.26 1.05 1.16 1.29 1.31 1.33 1.29	no	g2443	NAD(P)-binding domain; Alcohol dehydrogenase superfamily, zinc-type; Polyketide synthase, enoylreductase; GroES-like			
**DEL73**	scaffold7|size2161850	675,071	676,401	1,330	11	BP57 CP367 CP61 MCO448 PL429.300 PL429.5000 PL429.600 PL429.miseq PL448 SZMC21154 SZMC8029 SZMC8092 SZMC8094 SZMC8095	1.28 1.21 1.25 1.19 1.22 1.21 1.13 1.41 1.13 1.09 1.17 1.24 1.03 1.05	no	.				
**DEL74**	scaffold7|size2161850	738,355	743,173	4,818	11	BP57 CP367 CP61 MCO448 PL429.300 PL429.5000 PL429.600 PL429.miseq PL448 SZMC21154 SZMC8029 SZMC8092 SZMC8094 SZMC8095	0.94 0.90 0.94 1.11 0.94 1.29 0.88 0.96 0.98 0.81 0.89 0.93 0.92 1.01	no	g2501 g2502 g2503 g2504 g2505	Transcriptional adaptor 2; Zinc finger, ZZ-type; Homeodomain-like; SANT/Myb domain; SWIRM domain; SANT domain; Ribosomal protein S17e; Ribosomal protein S17e, conserved site; Transcriptional adaptor 2; Zinc finger, ZZ-type; Homeodomain-like; SANT/Myb domain; SWIRM domain; SANT domain			
**DEL75**	scaffold7|size2161850	882,532	887,526	4,994	1	PL429.300 PL429.5000 PL429.600 PL429.miseq	0.94 1.20 0.99 0.90	no	g2576 g2577	Ferredoxin reductase-type FAD-binding domain; Riboflavin synthase-like beta-barrel; Ferric reductase transmembrane component-like domain; FAD-binding 8; Ferric reductase, NAD binding; Ferredoxin reductase-type FAD-binding domain; Riboflavin synthase-like beta-barrel; Ferric reductase transmembrane component-like domain; FAD-binding 8; Ferric reductase, NAD binding			
**DEL76**	scaffold7|size2161850	1,069,267	1,072,457	3,190	7	MCO448 PL429.300 PL429.600 PL429.miseq PL448 SZMC21154 SZMC8029 SZMC8092 SZMC8095	1.30 1.26 1.34 1.46 1.32 1.19 1.29 1.32 1.32	no	g2663 g2664 g2665	RmlC-like cupin domain; Acireductone dioxygenase ARD family; RmlC-like jelly roll fold; F-box domain, cyclin-like; Pre-mRNA-splicing factor 38			
**DEL77**	scaffold7|size2161850	1,151,054	1,152,251	1,197	11	BP57 CP367 CP61 MCO448 PL429.300 PL429.5000 PL429.600 PL448 SZMC21154 SZMC8029 SZMC8092 SZMC8094 SZMC8095	0.59 1.44 1.34 0.83 1.41 1.35 1.47 0.86 1.23 1.30 1.21 1.02 1.08	no	g2704	Protein of unknown function DUF1748, fungi			
**DEL78**	scaffold7|size2161850	1,280,575	1,284,518	3,943	11	BP57 CP367 CP61 MCO448 PL429.300 PL429.5000 PL429.600 PL429.miseq PL448 SZMC21154 SZMC8029 SZMC8092 SZMC8094 SZMC8095	0.38 1.11 1.08 0.85 1.22 1.34 1.32 1.19 0.95 1.11 1.18 1.14 1.03 0.97	no	g2762 g2763	NAD(P)-binding domain; NAD-dependent epimerase/dehydratase; LicD			
**DEL79**	scaffold7|size2161850	1,614,227	1,618,443	4,216	11	BP57 CP367 CP61 MCO448 PL429.300 PL429.5000 PL429.600 PL429.miseq PL448 SZMC21154 SZMC8029 SZMC8092 SZMC8094 SZMC8095	1.04 0.97 1.07 0.91 1.06 1.25 1.12 0.98 0.94 1.01 1.10 1.09 0.96 0.96	no	g2910 g2911	Heat shock protein 9/12; Lipase, secreted			
**DEL80**	scaffold7|size2161850	1,628,852	1,632,129	3,277	11	BP57 CP367 CP61 MCO448 PL429.300 PL429.5000 PL429.600 PL429.miseq PL448 SZMC21154 SZMC8029 SZMC8092 SZMC8094 SZMC8095	1.14 1.20 1.17 0.96 1.11 1.35 1.33 1.24 1.10 1.04 1.10 1.14 0.94 0.92	no	g2917 g2918 g2919	Thymidylate synthase; Thymidylate synthase/dCMP hydroxymethylase domain; Thymidylate synthase, active site			
**DEL81**	scaffold7|size2161850	1,667,693	1,671,565	3,872	11	BP57 CP367 CP61 MCO448 PL429.300 PL429.5000 PL429.600 PL429.miseq PL448 SZMC21154 SZMC8029 SZMC8092 SZMC8094 SZMC8095	1.01 1.11 0.97 1.25 1.05 1.48 1.11 0.97 1.08 0.93 1.02 1.00 1.12 1.16	no	g2934 g2935				
**DEL82**	scaffold7|size2161850	2,097,824	2,099,217	1,393	5	CP61 PL429.300 PL429.5000 PL429.600 SZMC21154 SZMC8094 SZMC8095	1.26 1.14 1.30 1.14 1.08 2.50 1.20	no	g3114	CDR ABC transporter			
**DEL83**	scaffold7|size2161850	2,127,991	2,130,943	2,952	8	MCO448 PL429.300 PL429.5000 PL429.600 PL429.miseq PL448 SZMC21154 SZMC8029 SZMC8092 SZMC8094 SZMC8095	0.73 0.64 0.68 0.61 0.84 0.69 0.73 0.75 0.82 2.69 0.83	no	g3130 g3131	DNA primase, small subunit, eukaryotic/archaeal; DNA primase, small subunit; Major facilitator superfamily domain, general substrate transporter; Major facilitator superfamily; Major facilitator superfamily domain			
**DEL84**	scaffold9|size263853	38,600	41,200	2,600	11	BP57 CP367 CP61 MCO448 PL429.300 PL429.5000 PL429.600 PL429.miseq PL448 SZMC21154 SZMC8029 SZMC8092 SZMC8094 SZMC8095	1.04 1.03 1.09 0.72 0.97 1.13 1.00 0.88 0.88 1.01 1.05 1.04 0.89 0.79	no	g86 g87	Tricalbin; C2 membrane targeting protein; C2 calcium-dependent membrane targeting; C2 calcium/lipid-binding domain, CaLB; Ribosomal protein S3Ae			
