# Supplementary material for: The Genomic Aftermath of Hybridization in the Opportunistic Pathogen Candida metapsilosis
Source: PLoS Genet. 2015 Oct 30;11(10):e1005626. doi: 10.1371/journal.pgen.1005626 (PMC4627764; doi:10.1371/journal.pgen.1005626)
Supplement: S5 Table — Clusters of orthologous genes that differ in the number of members between C. parapsilosis species complex are given. For each cluster, the table lists: cluster ID, total number of genes in the cluster, number of members and their IDs for C. metapsilosis, C. orthopsilosis and C. parapsilosis; functional annotations for cluster members; difference in number of members between C. metapsilosis, C. parapsilosis and C. orthopsilosis. For simplicity, several functional classes are provided with color background. (PDF) [file pgen.1005626.s019.pdf]

cluster_id	#me	#	CANME	#	CANOR	#	CANPA	functions	CANME -	CANME - CANOR	LEGEND
1	23	7	g1003.t1 g1	6	CORT_0G00120	10	CPAR2_300590	Major facilitator superfamily domain, general substrate transporter; Major facilitator superfamily; Major facilitator superfamily domain	-3	1	Hyphally-regulated
2	23	12	g1626.t1 g1	6	CORT_0B02520	5	CPAR2_502340	GPR1/FUN34/yaaH	7	6	membrane
3	21	7	g1071.t1 g1	6	CORT_0C04690	8	CPAR2_300010	ABC transporter-like; ABC transporter, conserved site; CDR ABC transporter; AAA+ ATPase domain; ABC-2 type transporter; Pleiotropic drug r	-1	1	Major facilitator superfamily
4	21	5	g1756.t1 g1	2	CORT_0C02750	14	CPAR2_106910	PT repeat; Hyphally-regulated cell wall protein, N-terminal	-9	3	*porter
5	20	7	g1061.t1 g2	6	CORT_0B00170	7	CPAR2_300110	Extracellular membrane protein, CFEM domain; Extracellular membrane protein, CFEM domain, fungi	0	1	Agglutinin-like
6	20	9	g1383.t1 g2	4	CORT_0A00350	7	CPAR2_701630	Tetrapeptide transporter, OPT1/isp4; Oligopeptide transporter OPT superfamily	2	5	Peptidase
7	18	5	g3873.t1 g5	4	CORT_0C00760	9	CPAR2_400100	Major facilitator superfamily domain, general substrate transporter; Major facilitator superfamily; General substrate transporter; Major facilitator s	-4	1	Flocculin
8	18	7	g2616.t1 g2	4	CORT_0A06610	7	CPAR2_207250	Major facilitator superfamily domain, general substrate transporter; Major facilitator superfamily	0	3	Lipase
9	17	5	g1142.t1 g1	5	CORT_0B00800	7	CPAR2_303790	Uncharacterised domain Flo11-reatedl, N-terminal; Fibrogen-binding, domain 1; Agglutinin-like protein, N-terminal; Flocculin type 3 repeat; Adh	-2	0	
10	17	7	g44.t1 g550	4	CORT_0B09350	6	CPAR2_108210	Major facilitator superfamily domain, general substrate transporter; Sugar/inositol transporter; General substrate transporter; Major facilitator su	1	3	
11	16	8	g1233.t1 g1	3	CORT_0E04910	5	CPAR2_302730	Steryl acetyl hydrolase	3	5	
12	16	4	g2859.t1 g2	5	CORT_0A06440	7	CPAR2_103130	Pyridine nucleotide-disulphide oxidoreductase, class-II; NAD(P)-binding domain; Amino acid/polyamine transporter I; FAD-dependent pyridine n	-3	-1	
13	15	4	g2334.t1 g2	4	CORT_0A11680	7	CPAR2_203150	Amino acid/polyamine transporter I	-3	0	
14	15	3	g1683.t1 g1	7	CORT_0G03030	5	CPAR2_702920	Major facilitator superfamily domain, general substrate transporter; Major facilitator superfamily	-2	-4	
15	15	9	g39.t1 g40.t	2	CORT_0C01430	4	CPAR2_807600	Acyl-CoA oxidase/dehydrogenase, central domain; Acyl-CoA oxidase; Acyl-CoA oxidase, C-terminal; Acyl-CoA oxidase/dehydrogenase, type 1;	5	7	
16	13	6	g1973.t1 g2	2	CORT_0D03890	5	CPAR2_203780	Cytochrome P450, E-class, CYP52; Cytochrome P450, E-class, group II; Cytochrome P450; Cytochrome P450, conserved site	1	4	
17	13	4	g2033.t1 g2	4	CORT_0C06190	5	CPAR2_204150	Flavodoxin/nitric oxide synthase; Flavoprotein WrbA; NADPH-dependent FMN reductase	-1	0	
18	12	5	g5254.t1 g5	4	CORT_0B06800	3	CPAR2_105580	Metallophosphoesterase domain; RNA recognition motif domain; Nucleotide-binding, alpha-beta plait	2	1	
19	12	5	g904.t1 g90	2	CORT_0H01780	5	CPAR2_701660	AMP-dependent synthetase/ligase	0	3	
20	11	4	g2576.t1 g2	4	CORT_0A09370	3	CPAR2_210110	Ferredoxin reductase-type FAD-binding domain; Riboflavin synthase-like beta-barrel; Ferric reductase transmembrane component-like domain;	1	0	
21	11	2	g3470.t1 g3	4	CORT_0D00610	5	CPAR2_110430	D-isomer specific 2-hydroxyacid dehydrogenase, catalytic domain; Pre-ATP-grasp fold; NAD(P)-binding domain; D-isomer specific 2-hydroxyaci	-3	-2	
22	10	4	g1904.t1 g4	3	CORT_0A01380	3	CPAR2_405490	ABC transporter, transmembrane domain; CheY-like superfamily; ABC transporter-like; HAMP linker domain; ABC transporter, conserved site;	1	1	
23	10	6	g5214.t1 g5	2	CORT_0C02740	2	CPAR2_806390	Hyphally-regulated cell wall protein, N-terminal	4	4	
24	10	4	g4583.t1 g4	2	CORT_0A02750	4	CPAR2_802690	Major facilitator superfamily domain, general substrate transporter; Major facilitator superfamily; Major facilitator superfamily domain	0	2	
25	10	5	g5128.t1 g5	3	CORT_0B05600	2	CPAR2_104460	NAD(P)-binding domain; NAD-dependent epimerase/dehydratase	3	2	
26	10	3	g3442.t1 g4	3	CORT_0A03070	4	CPAR2_203160	ATP-grasp fold, subdomain 1; ATP-grasp fold; ATP-grasp fold, subdomain 2; Glutamine amidotransferase type 1; Pre-ATP-grasp fold; Aminoac	-1	0	
27	9	4	g1700.t1 g2	1	CORT_0A00240	4	CPAR2_800120	NAD(P)-binding domain; Short-chain dehydrogenase/reductase, conserved site; Short-chain dehydrogenase/reductase SDR; Glucose/ribitol de	0	3	
28	9	4	g2255.t1 g2	2	CORT_0A12540	3	CPAR2_213230	Amino acid permease, conserved site; Amino acid/polyamine transporter I	1	2	
29	9	2	g4953.t1 g4	3	CORT_0B03810	4	CPAR2_102400	Peptidase aspartic, catalytic; Peptidase aspartic; Peptidase aspartic, active site; Peptidase A1	-2	-1	
30	9	4	g3562.t1 g3	3	CORT_0D04510	2	CPAR2_204420	Alcohol dehydrogenase, long-chain fatty; Glucose-methanol-choline oxidoreductase, N-terminal; Glucose-methanol-choline oxidoreductase, C-t	2	1	
31	9	4	g1437.t1 g1	2	CORT_0C07330	3	CPAR2_407980	Peptidase S10, serine carboxypeptidase, active site; Propeptide, carboxypeptidase Y; Peptidase S10, serine carboxypeptidase	1	2	
32	9	4	g3014.t1 g3	3	CORT_0D04710	2	CPAR2_101160	Methyltransferase type 11	2	1	
33	8	2	g34.t1 g598	3	CORT_0A05690	3	CPAR2_206340	HAD-like domain; Phosphoribosyl pyrophosphate synthetase, conserved site; Ribose-phosphate diphosphokinase; Phosphoribosyltransferase d	-1	-1	
34	8	3	g2797.t1 g4	3	CORT_0A03860	2	CPAR2_207780	Catalase-like domain; Catalase, mono-functional, haem-containing; Catalase, mono-functional, haem-containing, clades 1 and 3; Catalase core	1	0	
35	8	2	g3476.t1 g4	3	CORT_0B03410	3	CPAR2_102100	Major facilitator superfamily domain, general substrate transporter; Major facilitator superfamily; Major facilitator superfamily domain	-1	-1	
36	8	4	g1505.t1 g1	2	CORT_0G00410	2	CPAR2_700290	RHO protein GDP dissociation inhibitor; Immunoglobulin E-set; Rho GDP-dissociation inhibitor domain	2	2	
37	8	3	g3918.t1 g3	1	CORT_0B01330	4	CPAR2_501140	NAD(P)-binding domain; Short-chain dehydrogenase/reductase, conserved site; Short-chain dehydrogenase/reductase SDR; Glucose/ribitol de	-1	2	
38	8	4	g516.t1 g51	1	CORT_0E02090	3	CPAR2_107230	Major facilitator superfamily domain, general substrate transporter; General substrate transporter; Major facilitator superfamily domain	1	3	
39	8	2	g4716.t2 g4	3	CORT_0A04060	3	CPAR2_804030	Peptidase S8, subtilisin, Ser-active site; Peptidase S8/S53, subtilisin/kexin/sedolisin; Glycosyl transferase, family 48; Proteinase inhibitor, prope	-1	-1	
40	8	4	g4874.t1 g4	2	CORT_0B03080	2	CPAR2_101790	Mg2+ transporter protein, CorA-like/Zinc transport protein ZntB	2	2	
41	8	2	g4216.t1 g4	2	CORT_0F02740	4	CPAR2_601730	Acyl transferase/acyl hydrolase/lysophospholipase; Lysophospholipase, catalytic domain	-2	0	
42	8	2	g358.t1 g84	3	CORT_0D06640	3	CPAR2_403590	Glycosyl transferase, family 15	-1	-1	
43	8	2	g5735.t1 g5	3	CORT_0C01230	3	CPAR2_806680	Anp1; Structural maintenance of chromosomes protein; RecF/RecN/SMC; SMCs flexible hinge	-1	-1	
44	7	3	g1880.t1 g1	2	CORT_0C04670	2	CPAR2_405260	Zn(2)-C6 fungal-type DNA-binding domain; Transcription factor, fungi	1	1	
45	7	3	g3448.t1 g5	1	CORT_0C00280	3	CPAR2_107010	Histone-fold; Histone H3; Histone core	0	2	
46	7	3	g3480.t1 g4	2	CORT_0B03160	2	CPAR2_101860	Amino acid permease, conserved site; Amino acid permease domain; Amino acid/polyamine transporter I	1	1	
47	7	3	g225.t1 g24	1	CORT_0A04310	3	CPAR2_800760	Major facilitator superfamily domain, general substrate transporter; Proton-dependent oligopeptide transporter family; PTR2 family proton/oligop	0	2	
48	7	5	g1076.t1 g1	1	CORT_0A00100	1	CPAR2_704320	Monooxygenase, FAD-binding; Aromatic-ring hydroxylase-like	4	4	
49	7	5	g4408.t1 g4	1	CORT_0B03710	1	CPAR2_102550	AMP-dependent synthetase/ligase	4	4	
50	7	3	g1422.t1 g1	2	CORT_0G01300	2	CPAR2_701130	Acyl transferase/acyl hydrolase/lysophospholipase; Lysophospholipase, catalytic domain	1	1	
51	7	2	g4819.t1 g4	2	CORT_0A05100	3	CPAR2_805010	Carboxymuconolactone decarboxylase	-1	0	

52	7	3	g4603.t1 g5	2	CORT_0A02940	2	CPAR2_802890	Major facilitator superfamily domain, general substrate transporter; Major facilitator superfamily; Major facilitator superfamily domain	1	1
53	7	3	g1810.t1 g2	2	CORT_0A11980	2	CPAR2_212680	Helicase, superfamily 1/2, ATP-binding domain; Homeodomain-like; SNF2-related; SANT/Myb domain; ATPase, nucleosome remodelling ISWI,	1	1
54	7	3	g2851.t1 g2	2	CORT_0A06360	2	CPAR2_207000	Enolase, N-terminal; Enolase, C-terminal; Enolase, conserved site; Enolase	1	1
55	7	1	g2722.t1	3	CORT_0A07900	3	CPAR2_208620	Peptidase C13, legumain; Helicase, superfamily 1/2, ATP-binding domain; DNA polymerase alpha, subunit B N-terminal; Helicase, C-terminal;	-2	-2
56	7	2	g3858.t1 g4	3	CORT_0A04760	2	CPAR2_501780	Polyketide synthase, enoylreductase; Alcohol dehydrogenase superfamily, zinc-type; GroES-like; Alcohol dehydrogenase GroES-like; Alcohol d	0	-1
57	7	4	g1565.t1 g2	2	CORT_0G04170	1	CPAR2_103100	F-box domain, cyclin-like	3	2
58	7	2	g127.t1 g64	3	CORT_0B11030	2	CPAR2_109920	Major facilitator superfamily domain, general substrate transporter; Sugar transporter, conserved site; General substrate transporter; Major facili	0	-1
59	7	2	g4044.t1 g5	2	CORT_0B06970	3	CPAR2_105760	ATPase, P-type, K/Mg/Cd/Cu/Zn/Na/Ca/Na/H-transporter; ATPase, P-type, cytoplasmic domain N; ATPase, P-type, transmembrane domain; A	-1	0
60	7	3	g1661.t1 g1	2	CORT_0G03360	2	CPAR2_703190	Lipase, secreted	1	1
61	7	2	g4999.t1 g6	2	CORT_0B04350	3	CPAR2_103080	ThiJ/PfpI	-1	0
62	7	3	g1933.t1 g5	2	CORT_0B08170	2	CPAR2_106960	Acetate-CoA ligase; Acyl-CoA synthase, domain of unknown function DUF3448; AMP-dependent synthetase/ligase; Domain of unknown functio	1	1
63	7	3	g1525.t1 g1	2	CORT_0G00220	2	CPAR2_700110	Major facilitator superfamily domain, general substrate transporter; Major facilitator superfamily; Major facilitator superfamily domain	1	1
64	7	3	g1219.t2 g2	2	CORT_0C06580	2	CPAR2_302950	Luciferase-like domain; Nitrilotriacetate monooxygenase component A/pristinamycin IIA synthase subunit A	1	1
65	7	3	g212.t1 g21	2	CORT_0A00770	2	CPAR2_800690 CPAR2_801040		1	1
66	7	4	g3081.t1 g3	2	CORT_0F00580	1	CPAR2_100490	Major facilitator superfamily domain, general substrate transporter; Major facilitator superfamily; General substrate transporter; Major facilitator s	3	2
67	7	1	g3303.t1	3	CORT_0D01800	3	CPAR2_201740	Neuraminidase; VPS10	-2	-2
68	7	2	g2798.t1 g2	3	CORT_0A07100	2	CPAR2_207750	LicD	0	-1
69	7	1	g3530.t1	3	CORT_0D04210	3	CPAR2_204110	Alpha-mannosyltransferase	-2	-2
70	7	1	g4468.t1	3	CORT_0A01725	3	CPAR2_801655	Major facilitator superfamily domain, general substrate transporter; Sugar transporter, conserved site; General substrate transporter; Major facili	-2	-2
71	6	3	g2456.t1 g2	2	CORT_0A10520	1	CPAR2_101810	Short-chain dehydrogenase/reductase, conserved site; NAD(P)-binding domain; Glucose/ribitol dehydrogenase; Short-chain dehydrogenase/re	2	1
72	6	3	g4227.t1 g4	1	CORT_0F02900	2	CPAR2_601860	Glycine cleavage T-protein, N-terminal; Glycine cleavage system T protein; Glycine cleavage T-protein, C-terminal barrel	1	2
73	6	1	g5028.t1	3	CORT_0B04580	2	CPAR2_103330	Domain of unknown function DUF3533	-1	-2
74	6	1	g5362.t1	3	CORT_0B07920	2	CPAR2_106670	Amino acid permease domain; Amino acid/polyamine transporter I	-1	-2
75	6	2	g4613.t1 g4	1	CORT_0A03060	3	CPAR2_803020	Major facilitator superfamily domain, general substrate transporter; Major facilitator superfamily	-1	1
76	6	1	g4235.t1	3	CORT_0B04740	2	CPAR2_103530	Major facilitator superfamily domain, general substrate transporter; Major facilitator superfamily	-1	-2
77	6	3	g55.t1 g561	1	CORT_0B10520	2	CPAR2_109400	NADH:ubiquinone oxidoreductase intermediate-associated protein 30; Galactose-binding domain-like	1	2
78	6	3	g669.t1 g67	1	CORT_0E00570	2	CPAR2_400440 CPAR2_400540		1	2
79	6	1	g181.t1	2	CORT_0E03680	3	CPAR2_403670	Amino acid permease, conserved site; Amino acid permease domain; Amino acid/polyamine transporter I	-2	-1
80	6	4	g366.t1 g82	1	CORT_0E03470	1	CPAR2_403560		3	3
81	6	1	g1016.t1	2	CORT_0H00650	3	CPAR2_300580	Ferredoxin reductase-type FAD-binding domain; Riboflavin synthase-like beta-barrel; Ferric reductase transmembrane component-like domain;	-2	-1
82	6	4	g1320.t1 g1	1	CORT_0C03440	1	CPAR2_805720		3	3
83	5	1	g5815.t1	2	CORT_0C01600	2	CPAR2_807530	Manganese/iron superoxide dismutase, C-terminal; Manganese/iron superoxide dismutase; Pal1 cell morphology; Manganese/iron superoxide	-1	-1
84	5	2	g1825.t1 g1	2	CORT_0A05820	1	CPAR2_404710		1	0
85	5	1	g3074.t1	2	CORT_0F00660	2	CPAR2_100560	Amino acid permease, conserved site; Amino acid permease domain; Amino acid permease, fungi; Amino acid/polyamine transporter I	-1	-1
86	5	1	g5000.t1	2	CORT_0B04360	2	CPAR2_103090	Alkaline-phosphatase-like, core domain; Alkaline phosphatase-like, alpha/beta/alpha; Sulfatase; Sulfatase, conserved site	-1	-1
87	5	1	g888.t1	2	CORT_0H01930	2	CPAR2_805560	Ribosomal protein L27; Ribosomal protein L27, conserved site; Uncharacterised protein family UPF0303; Domain of unknown function DUF336	-1	-1
88	5	1	g5765.t1	2	CORT_0C02160	2	CPAR2_807000 CPAR2_807010		-1	-1
89	5	1	g4082.t1	2	CORT_0H02410	2	CPAR2_600370	Cytochrome c oxidase, subunit VIb; DNA/RNA-binding protein Alba-like	-1	-1
90	5	1	g5789.t1	2	CORT_0C01880	2	CPAR2_807260	Zn(2)-C6 fungal-type DNA-binding domain; Transcription factor, fungi	-1	-1
91	5	3	g54.t1 g561	1	CORT_0B10510	1	CPAR2_109450	HAD-like domain; IMP-specific 5-nucleotidase	2	2
92	5	1	g1438.t1	2	CORT_0A01170	2	CPAR2_700990	Sodium/solute symporter	-1	-1
93	5	2	g4226.t1 g4	1	CORT_0F02910	2	CPAR2_601850 CPAR2_601940		0	1
94	5	2	g3481.t1 g3	1	CORT_0D03660	2	CPAR2_203550	Pleckstrin homology-like domain	0	1
95	5	2	g4030.t1 g5	1	CORT_0B00180	2	CPAR2_500090	Ribosomal protein S27a; Ubiquitin conserved site; Ubiquitin supergroup; Ribosomal protein, zinc-binding domain; Ubiquitin; Ubiquitin subgroup	0	1
96	5	1	g5671.t1	2	CORT_0C03080	2	CPAR2_806050	Rieske [2Fe-2S] iron-sulphur domain; Ubiquinol-cytochrome c reductase, iron-sulphur subunit; Rieske iron-sulphur protein; HECT; Zinc finger, L	-1	-1
97	5	1	g1877.t1	2	CORT_0C04630	2	CPAR2_405220	Ribonuclease H2, subunit B; Zinc finger, CHCC-type	-1	-1
98	5	1	g1446.t1	2	CORT_0G01050	2	CPAR2_700890	Zn(2)-C6 fungal-type DNA-binding domain; Protein of unknown function DUF3468	-1	-1
99	5	2	g2031.t1 g2	1	CORT_0C01990	2	CPAR2_406780	RNA polymerase II-associated, Paf1	0	1
100	5	3	g508.t1 g50	1	CORT_0D06520	1	CPAR2_503040	Short-chain dehydrogenase/reductase, conserved site; Short-chain dehydrogenase/reductase SDR; Glucose/ribitol dehydrogenase; Armadillo-li	2	2
101	5	1	g4343.t1	2	CORT_0A06890	2	CPAR2_207540	Major facilitator superfamily domain, general substrate transporter; Major facilitator superfamily; Major facilitator superfamily domain	-1	-1
102	5	2	g126.t1 g64	1	CORT_0B11040	2	CPAR2_109930	Glycoside hydrolase, superfamily; Glycosyl hydrolase, family 13, all-beta; Glycoside hydrolase, catalytic domain; Glycosyl hydrolase, family 13,	0	1
103	5	1	g580.t1	2	CORT_0E01450	2	CPAR2_401400	Zinc finger, HIT-type; Lipase, class 3	-1	-1

104	5	3	g4435.t1 g4	1	CORT_0A01550	1	CPAR2_801480	Major facilitator superfamily domain, general substrate transporter; Major facilitator superfamily; Major facilitator superfamily domain	2	2	
105	5	1	g2133.t1	2	CORT_0C07200	2	CPAR2_407860	RNA recognition motif domain; Nucleotide-binding, alpha-beta plait	-1	-1	
106	5	1	g950.t1	2	CORT_0H01360	2	CPAR2_301360	ATPase, P-type, K/Mg/Cd/Cu/Zn/Na/Ca/Na/H-transporter; YrdC-like domain; ATPase, P-type, cytoplasmic domain N; ATPase, P-type cation-tra	-1	-1	
107	5	1	g3830.t1	2	CORT_0B02240	2	CPAR2_502080	Major facilitator superfamily domain, general substrate transporter; Major facilitator superfamily; Per1-like	-1	-1	
108	5	1	g3169.t1	2	CORT_0D00450	2	CPAR2_200360	Translation Initiation factor eIF- 4e; Eukaryotic translation initiation factor 4E (eIF-4E), conserved site; Translation Initiation factor eIF- 4e-like d	-1	-1	
109	5	1	g2777.t1	2	CORT_0A07340	2	CPAR2_208010	Translation elongation/initiation factor/Ribosomal, beta-barrel; Translation elongation factor EFG/EF2, C-terminal; Translation elongation factor	-1	-1	
110	5	1	g4244.t1	2	CORT_0F03030	2	CPAR2_602060	Zn(2)-C6 fungal-type DNA-binding domain; Aflatoxin biosynthesis regulatory protein	-1	-1	
111	5	2	g4669.t1 g4	1	CORT_0A03600	2	CPAR2_803580	RNA polymerase, alpha subunit; RNA polymerase, N-terminal; DNA-directed RNA pol I, largest subunit; RNA polymerase Rpb1, domain 4; RNA	0	1	
112	5	2	g5505.t1 g5	1	CORT_0B09425	2	CPAR2_108240 CPAR2_108310		0	1	
113	5	1	g4955.t2	1	CORT_0B03860	3	CPAR2_102610	Peptidase aspartic, catalytic; Peptidase aspartic; Peptidase aspartic, active site; Peptidase A1	-2	0	
114	5	1	g3814.t1	2	CORT_0B02410	2	CPAR2_502230	ATPase, AAA-type, core; DNA polymerase III, clamp loader complex, gamma/delta/delta subunit, C-terminal; AAA+ ATPase domain; Stretch-ac	-1	-1	
115	5	2	g5893.t1 g5	1	CORT_0C00800	2	CPAR2_808350 CPAR2_808370		0	1	
116	5	2	g4611.t1 g4	1	CORT_0A03090	2	CPAR2_802980 CPAR2_803090		0	1	
117	5	1	g2824.t2	2	CORT_0A06850	2	CPAR2_207500	Protein kinase, ATP binding site; tRNA delta(2)-isopentenylpyrophosphate transferase; Serine/threonine-protein kinase, active site; Protein kina	-1	-1	
118	5	1	g1185.t1	2	CORT_0E05430	2	CPAR2_303290	Peroxin/Dysferlin domain; Sirtuin family; Sirtuin family, catalytic core domain; Sirtuin family, catalytic core small domain	-1	-1	
119	5	1	g5760.t1	2	CORT_0C02230	2	CPAR2_806930	Thioredoxin-like fold; Glutathione S-transferase/chloride channel, C-terminal; Glutathione S-transferase, C-terminal; Glutathione S-transferase,	-1	-1	
120	5	1	g1463.t1	2	CORT_0G00870	2	CPAR2_700710	Protein kinase, ATP binding site; Translation protein SH3-like; Serine/threonine-protein kinase, active site; Protein kinase, catalytic domain; Seri	-1	-1	
121	5	1	g4895.t2	2	CORT_0B03290	2	CPAR2_101990	Uncharacterised protein family UPF0041; Zn(2)-C6 fungal-type DNA-binding domain	-1	-1	
122	5	1	g4066.t1	2	CORT_0H02580	2	CPAR2_600210	DNA polymerase V; Armadillo-type fold; ATPase, F1 complex, epsilon subunit, mitochondrial; Armadillo-like helical	-1	-1	
123	5	1	g1060.t2	2	CORT_0H00190	2	CPAR2_300130	Adenosine deaminase/editase; Ferric reductase transmembrane component-like domain; FAD-binding 8; Ferric reductase, NAD binding; Tetratr	-1	-1	
124	5	1	g5557.t1	2	CORT_0B09860	2	CPAR2_108140	Transcription factor TFIIB; Cyclin-like; Zinc finger, TFIIB-type; Zinc finger, GATA-type; Transcription factor TFIIB, cyclin-like domain; Transcriptio	-1	-1	
125	5	1	g5487.t1	2	CORT_0B09180	2	CPAR2_108000	Major facilitator superfamily domain, general substrate transporter; N-acetylglutamate synthase, fungal; Hly-III-related; Domain of unknown func	-1	-1	
126	5	1	g5223.t1	2	CORT_0B06430	2	CPAR2_105230	CTLH/CRA C-terminal to LisH motif domain; tRNA (uracil-O(2)-)-methyltransferase	-1	-1	
127	5	1	g5552.t1	2	CORT_0B09800	2	CPAR2_108710	HAD-superfamily hydrolase, subfamily IIB; Trehalose-phosphatase; Glycosyl transferase, family 20; HAD-like domain; Translocation protein Sec	-1	-1	
128	5	1	g2391.t1	2	CORT_0A11200	2	CPAR2_211860	AMP deaminase; Adenosine/AMP deaminase domain; Adenosine/AMP deaminase active site	-1	-1	
129	5	1	g5869.t1	2	CORT_0C01050	2	CPAR2_808080	TATA element modulatory factor 1 DNA binding; TATA element modulatory factor 1 TATA binding	-1	-1	
130	5	1	g1305.t1	2	CORT_0E04510	2	CPAR2_302360	Myb domain; Homeodomain-like; SANT/Myb domain; NAD(P)-binding domain; Tetrahydrofolate dehydrogenase/cyclohydrolase, catalytic domai	-1	-1	
131	5	2	g4935.t1 g4	2	CORT_0B03690	1	CPAR2_102410	Peptidase A1; Peptidase aspartic; Peptidase aspartic, active site; Peptidase aspartic, catalytic	1	0	
132	5	1	g2288.t1	2	CORT_0A12220	2	CPAR2_212920 CPAR2_212925		-1	-1	
133	5	1	g1665.t1	2	CORT_0G03320	2	CPAR2_703160	XPA; Histidine biosynthesis; Imidazole glycerol phosphate synthase, subunit H; Aldolase-type TIM barrel; DNA binding domain, putative; Imidaz	-1	-1	
134	5	1	g495.t1	2	CORT_0E02290	2	CPAR2_402240	LUC7-related; F-box domain, cyclin-like	-1	-1	
135	5	1	g3105.t1	2	CORT_0F00340	2	CPAR2_100230	Cytochrome b, succinate dehydrogenase small subunit, CybS; Ribosomal protein S14	-1	-1	
136	5	1	g3345.t1	2	CORT_0D02240	2	CPAR2_202190	Ribosomal protein S4, conserved site; Ribosomal protein S4/S9; RNA-binding S4 domain; Modification methylase HemK	-1	-1	
137	5	1	g1473.t1	2	CORT_0G00750	2	CPAR2_700600	Zinc finger, C6HC-type; Zinc finger, RING-type; Zinc finger, RING/FYVE/PHD-type; Zinc finger, RING-type, conserved site	-1	-1	
138	5	1	g4080.t1	2	CORT_0H02440	2	CPAR2_600340	Domain of unknown function DUF814; CCR4-Not complex component, Not1, C-terminal; CCR4-Not complex, Not1 subunit, domain of unknown	-1	-1	
139	5	1	g2025.t1	2	CORT_0C06120	2	CPAR2_406700	Sterol O-acyltransferase, ACAT/DAG/ARE types; Protein of unknown function DUF3112; Membrane bound O-acyl transferase, MBOAT	-1	-1	
140	5	1	g5250.t1	2	CORT_0B06730	2	CPAR2_105510	Single hybrid motif; Biotin carboxylation domain; Allophanate hydrolase subunit 1; Biotin carboxylase, C-terminal; Cyclophilin-like peptidyl-prolyl	-1	-1	
141	5	1	g1699.t1	2	CORT_0G02880	2	CPAR2_702770	WD40/YVTN repeat-like-containing domain; Late secretory pathway protein AVL9; WD40 repeat; WD40-repeat-containing domain	-1	-1	
142	5	1	g3772.t1	2	CORT_0B02870	2	CPAR2_502680	Aminoacyl-tRNA synthetase, class 1a, anticodon-binding; Valyl/Leucyl/Isoleucyl-tRNA synthetase, class I, anticodon-binding; Methionyl-tRNA s	-1	-1	
143	5	1	g390.t1	2	CORT_0E03290	2	CPAR2_403270	Thioredoxin-like fold; ER lumen protein retaining receptor; Thioredoxin domain	-1	-1	
144	5	1	g5188.t3	2	CORT_0B06160	2	CPAR2_104960	Concanavalin A-like lectin/glucanases superfamily; Legume-like lectin; Concanavalin A-like lectin/glucanase, subgroup	-1	-1	
145	5	1	g1096.t3	2	CORT_0E06390	2	CPAR2_304200	tRNA-dihydrouridine synthase; Aldolase-type TIM barrel; tRNA-dihydrouridine synthase, conserved site; Bromodomain; Bromodomain, conserv	-1	-1	
146	5	1	g4721.t1	2	CORT_0A04130	2	CPAR2_804100	Zinc finger, RanBP2-type; ATPase-like, ATP-binding domain; Ribonuclease H-like domain; Signal transduction histidine kinase, core; Branched-	-1	-1	
147	5	3	g325.t1 g33	1	CORT_0E03930	1	CPAR2_403920	Protein of unknown function DUF3445	2	2	
148	5	1	g5372.t1	2	CORT_0B08010	2	CPAR2_106780	Immunoglobulin E-set; RHO protein GDP dissociation inhibitor; Nucleotide-binding, alpha-beta plait; Cleavage stimulation factor subunit 2, hing	-1	-1	
149	5	1	g2656.t1	2	CORT_0A08560	2	CPAR2_209270	HSP20-like chaperone; Alpha crystallin/Hsp20 domain	-1	-1	
150	5	3	g3.t1 g3037	1	CORT_0F01030	1	CPAR2_100760	Tetratricopeptide repeat; Tetratricopeptide-like helical; Tetratricopeptide repeat-containing domain; Tetratricopeptide TPR-1	2	2	
151	5	1	g1172.t1	2	CORT_0E05560	2	CPAR2_303420	Pseudouridine synthase, catalytic domain; Zinc finger, C2H2-type matrin; CMP/dCMP deaminase, zinc-binding; Domain of unknown function D	-1	-1	
152	5	2	g3216.t1 g3	1	CORT_0D01210	2	CPAR2_200890 CPAR2_201160		0	1	
153	5	1	g3346.t1	2	CORT_0D02260	2	CPAR2_202210	DNA mismatch repair protein MutS-like, N-terminal; DNA mismatch repair protein MutS, core; DNA mismatch repair protein MutS, connector; D	-1	-1	
154	5	1	g943.t1	2	CORT_0H01440	2	CPAR2_301440	NmrA-like; NAD(P)-binding domain	-1	-1	
155	5	1	g3593.t1	2	CORT_0D04810	2	CPAR2_204700	Acyl transferase/acyl hydrolase/lysophospholipase; Acyl transferase; Malonyl-CoA ACP transacylase, ACP-binding; Formate-tetrahydrofolate lig	-1	-1	

156	5	2	g2603.t1 g3	1	CORT_0A09090	2	CPAR2_107990	Acyl-CoA thioesterase	0	1		
157	5	1	g1211.t1	2	CORT_0E05160	2	CPAR2_303020	Ham1-like protein; Domain of unknown function DUF1713, mitochondria	-1	-1		
158	5	1	g5934.t1	2	CORT_0C00450	2	CPAR2_808690	MIR motif; Glycosyl transferase, family 39; FeS cluster insertion protein; Glycosyltransferase 39 like; FeS cluster biogenesis	-1	-1		
159	5	2	g3826.t1 g4	1	CORT_0B02300	2	CPAR2_502140	Glycoside hydrolase, superfamily; Glycoside hydrolase, family 18, catalytic domain; Flocculin type 3 repeat; Glycoside hydrolase, chitinase activ	0	1		
160	5	1	g5614.t1	2	CORT_0B10530	2	CPAR2_109390	Short-chain dehydrogenase/reductase SDR; NAD(P)-binding domain; Glucose/ribitol dehydrogenase	-1	-1		
161	5	1	g2032.t1	2	CORT_0C01980	2	CPAR2_406790	NADP-dependent oxidoreductase domain; Aldo/keto reductase, conserved site; Aldo/keto reductase; Aldo/keto reductase subgroup	-1	-1		
162	5	1	g2176.t1	2	CORT_0A13300	2	CPAR2_213980	Transmembrane receptor, eukaryota; Winged helix-turn-helix transcription repressor DNA-binding; Proteasome component (PCI) domain	-1	-1		
163	5	1	g4707.t1	2	CORT_0A03960	2	CPAR2_803930	Rossmann-like alpha/beta/alpha sandwich fold; Zinc finger, U1-type; Phosphoadenosine phosphosulphate reductase	-1	-1		
164	5	2	g583.t1 g58	2	CORT_0E01500	1	CPAR2_401450	Major facilitator superfamily domain, general substrate transporter; Sugar transporter, conserved site; General substrate transporter; Sugar/inosi	1	0		
165	5	1	g1604.t1	2	CORT_0G03730	2	CPAR2_703550	Translation elongation factor EF1A/initiation factor IF2gamma, C-terminal; Translation elongation/initiation factor/Ribosomal, beta-barrel; Amida	-1	-1		
166	5	1	g2890.t1	2	CORT_0A06080	2	CPAR2_206730	Mitochondrial carrier domain; Histidine phosphatase superfamily, clade-1; Mitochondrial substrate/solute carrier	-1	-1		
167	5	1	g763.t1	2	CORT_0D07350	2	CPAR2_503880	WD40-repeat-containing domain; Major facilitator superfamily domain, general substrate transporter; Quinoprotein amine dehydrogenase, beta	-1	-1		
168	5	1	g3693.t1	2	CORT_0D05830	2	CPAR2_205710	Peptidase cysteine/serine, trypsin-like; Peptidase S64, Ssy5	-1	-1		
169	5	1	g1711.t2	2	CORT_0G02630	2	CPAR2_702450	Cyclin-like; DNA glycosylase; Cyclin, N-terminal; HhH-GPD domain; 8-oxoguanine DNA glycosylase, N-terminal; Cyclin, C-terminal; Helix-turn-h	-1	-1		
170	5	2	g1540.t1 g3	1	CORT_0D00920	2	CPAR2_200830	Amino acid permease domain; Amino acid/polyamine transporter I	0	1		
171	5	3	g3111.t2 g3	1	CORT_0F00410	1	CPAR2_100300	Major facilitator superfamily domain, general substrate transporter; Major facilitator superfamily; Major facilitator superfamily domain	2	2		
172	5	1	g1085.t1	2	CORT_0E06520	2	CPAR2_304320	Beta-hexosaminidase subunit alpha/beta; Streptomyces cyclase/dehydrase; Glycoside hydrolase, superfamily; Glycoside hydrolase, catalytic d	-1	-1		
173	5	3	g2942.t1 g2	1	CORT_0A05750	1	CPAR2_206400	Armadillo-type fold; HEAT, type 2; Armadillo-like helical; Importin-beta, N-terminal	2	2		
174	5	1	g348.t1	1	CORT_0E03950	3	CPAR2_301840 CPAR2_403940 CPAR2_805910		-2	0		
175	5	1	g2447.t1	2	CORT_0A10660	2	CPAR2_211320	Glycoside hydrolase, family 47	-1	-1		
176	5	1	g3202.t3	2	CORT_0D00810	2	CPAR2_200720	YL1 nuclear, C-terminal; Glucosidase II beta subunit-like	-1	-1		
177	5	2	g394.t1 g52	1	CORT_0B06750	2	CPAR2_105530	Sodium/solute symporter; Sodium/solute symporter, subgroup	0	1		
178	5	1	g5035.t1	2	CORT_0B04670	2	CPAR2_103460	Glycosyl transferase, family 8; WLM; UDP-glucose:Glycoprotein Glucosyltransferase	-1	-1		
179	5	1	g2336.t1	2	CORT_0A11730	2	CPAR2_212410	Heat shock chaperonin-binding; Tetratricopeptide repeat; Tetratricopeptide-like helical; Tetratricopeptide repeat-containing domain; Tetratricope	-1	-1		
180	5	1	g380.t1	2	CORT_0E03390	2	CPAR2_403370	Pyridine nucleotide-disulphide oxidoreductase, FAD/NAD(P)-binding domain; Domain of unknown function DUF202; SPX, N-terminal; Pyridine n	-1	-1		
181	5	1	g4936.t1	2	CORT_0B03680	2	CPAR2_102390	GPI-Mannosyltransferase II co-activator, Pga1; Endoplasmic reticulum, stress-associated Ramp4	-1	-1		
182	5	2	g1341.t1 g1	1	CORT_0G02190	2	CPAR2_600710	Aldolase-type TIM barrel; NADH:flavin oxidoreductase/NADH oxidase, N-terminal	0	1		
183	5	1	g5300.t1	2	CORT_0B07250	2	CPAR2_106020	INO80 complex, subunit Ies4; Anticodon-binding; Aminoacyl-tRNA synthetase, class II; Aminoacyl-tRNA synthetase, class II (G/ H/ P/ S), conse	-1	-1		
184	5	1	g5131.t2	2	CORT_0B05620	2	CPAR2_104380	Winged helix-turn-helix transcription repressor DNA-binding; SMAD/FHA domain; Transcription factor, fork head; Forkhead-associated (FHA) d	-1	-1		
185	5	1	g3335.t1	2	CORT_0D02130	2	CPAR2_202080	U6 snRNA-associated Sm-like protein LSm2; p53-like transcription factor, DNA-binding; Ribonucleoprotein LSM domain, eukaryotic/archaea-ty	-1	-1		
186	5	3	g4475.t1 g5	1	CORT_0C01070	1	CPAR2_808070	Epoxide hydrolase-like; Alpha/beta hydrolase fold-1	2	2		
187	5	1	g137.t1	2	CORT_0B10890	2	CPAR2_109810	Nucleic acid-binding, OB-fold-like; Kelch-type beta propeller; Exosome complex RNA-binding protein 1/RRP40/RRP4; Leucine carboxyl methyltr	-1	-1		
188	5	2	g5014.t1 g5	1	CORT_0B04520	2	CPAR2_103310	Amino acid permease domain; Amino acid/polyamine transporter I	0	1		
189	5	1	g5618.t1	2	CORT_0B10480	2	CPAR2_109420	IGR protein motif; Succinyl-CoA synthetase-like; NAD(P)-binding domain; Succinyl-CoA ligase, alpha subunit; ATP-citrate lyase/succinyl-CoA li	-1	-1		
190	5	1	g4642.t1	2	CORT_0A03410	2	CPAR2_803390	Ribosomal S11, conserved site; Ribosomal protein S11	-1	-1		
191	5	2	g3217.t1 g3	1	CORT_0D01200	2	CPAR2_200900	Carbon-nitrogen hydrolase; Uncharacterised protein family UPF0012, conserved site	0	1		
192	5	1	g810.t1	2	CORT_0D06930	2	CPAR2_503460	Telomerase holoenzyme, Est3 subunit	-1	-1		
193	5	1	g5467.t1	2	CORT_0B08960	2	CPAR2_107770	Small GTPase superfamily, Rab type; Small GTPase superfamily, Ras type; PAP/25A-associated; Small GTPase superfamily, Rho type; Nucleo	-1	-1		
194	5	1	g609.t1	2	CORT_0E01200	2	CPAR2_401160	Density-regulated protein DRP1; Tetratricopeptide-like helical; Translation initiation factor SUI1	-1	-1		
195	5	1	g3038.t1	2	CORT_0F00890	2	CPAR2_100790	Manganese/iron superoxide dismutase, C-terminal; Manganese/iron superoxide dismutase; TIP41-like protein; Manganese/iron superoxide dis	-1	-1		
196	5	1	g1990.t1	2	CORT_0C05690	2	CPAR2_406290	Protein kinase, ATP binding site; SMAD/FHA domain; Calcium/calmodulin-dependent/calcium-dependent protein kinase; Serine/threonine-prote	-1	-1		
197	5	1	g5461.t1	2	CORT_0B08890	2	CPAR2_107700	Dcp1-like decapping; Spt20 family	-1	-1		
198	5	1	g5064.t1	1	CORT_0B04940	3	CPAR2_103740	NPL4, zinc-binding putative; Polyubiquitin-tagged protein recognition complex, Npl4 component; Nuclear pore localisation protein NPL4; Nuclea	-2	0		
199	5	1	g3510.t1	2	CORT_0D03970	2	CPAR2_203860	Major facilitator superfamily domain, general substrate transporter; Major facilitator superfamily; Cytochrome P450; Major facilitator superfamily	-1	-1		
200	5	1	g4034.t1	2	CORT_0B00130	2	CPAR2_500040	Helicase, superfamily 1/2, ATP-binding domain; Sec63 domain; Helicase, C-terminal; DNA/RNA helicase, DEAD/DEAH box type, N-terminal; Im	-1	-1		
201	5	1	g5183.t2	2	CORT_0B06100	2	CPAR2_104900	MIR motif; Ribose-phosphate diphosphokinase; Phosphoribosyltransferase domain; Phosphoribosyl pyrophosphate synthetase, conserved site;	-1	-1		
202	5	2	g2073.t1 g2	1	CORT_0C06600	2	CPAR2_407230 CPAR2_407280		0	1		
203	5	1	g1599.t1	2	CORT_0G03900	2	CPAR2_703740	WD40/YVTN repeat-like-containing domain; Isy1-like splicing; WD40 repeat; WD40-repeat-containing domain; NUC153	-1	-1		
204	5	1	g2194.t1	2	CORT_0A13110	2	CPAR2_213850	Protein of unknown function DUF3661, vaculolar transmembrane; Protein of unknown function DUF1752, fungi	-1	-1		
205	5	1	g4967.t2	2	CORT_0B03990	2	CPAR2_102730	Ankyrin repeat; KilA, N-terminal/APSES-type HTH, DNA-binding; Ankyrin repeat-containing domain; Transcription regulator HTH, APSES-type	-1	-1		
206	5	1	g109.t1	2	CORT_0B08560	2	CPAR2_107360	3-oxo-5-alpha-steroid 4-dehydrogenase, C-terminal	-1	-1		
207	5	1	g4121.t1	2	CORT_0F01850	2	CPAR2_600790	Cytochrome d ubiquinol oxidase, 14kDa subunit; HIT-like domain; ATP adenylyltransferase, C-terminal; ATP adenylyltransferase	-1	-1		

208	5	2	g570.t1 g57	1	CORT_0E01590	2	CPAR2_401540	Peptidase M20; Peptidase M20, carboxypeptidase S; ArgE/DapE/ACY1/CPG2/YscS, conserved site; Peptidase M20, dimerisation	0	1		
209	5	1	g2068.t1	2	CORT_0C06550	2	CPAR2_407180	Protein kinase, ATP binding site; WW/Rsp5/WWP; Histone-lysine N-methyltransferase, SET2, fungi; Protein kinase, catalytic domain; Transcript	-1	-1		
210	5	1	g5687.t1	2	CORT_0C02920	2	CPAR2_806210	Pyridine nucleotide-disulphide oxidoreductase, FAD/NAD(P)-binding domain; Pyridine nucleotide-disulphide oxidoreductase, NAD-binding dom	-1	-1		
211	5	1	g623.t1	2	CORT_0E01040	2	CPAR2_401000	Eukaryotic translation initiation factor 3 subunit 8, N-terminal; tRNA(Ile)-lysidine/2-thiocytidine synthase; Uncharacterised protein family UPF002	-1	-1		
212	5	1	g5173.t1	2	CORT_0B05980	2	CPAR2_104780	Phosphoserine aminotransferase, subgroup; Phosphoserine aminotransferase; Pyridoxal phosphate-dependent transferase, major region, subd	-1	-1		
213	5	1	g5885.t1	2	CORT_0C00890	2	CPAR2_808250 CPAR2_808260		-1	-1		
214	5	1	g73.t1	2	CORT_0B11520	2	CPAR2_110410	Cystinosin/ERS1p repeat; Gamma-glutamyltranspeptidase	-1	-1		
215	4	1	g1111.t1	2	CORT_0E06220	1	CPAR2_304040	Concanavalin A-like lectin/glucanases superfamily; Beta-glucan synthesis-associated, SKN1; Concanavalin A-like lectin/glucanase, subgroup	0	-1		
216	4	2	g4207.t1 g4	1	CORT_0F02660	1	CPAR2_601650	Endonuclease/exonuclease/phosphatase	1	1		
217	4	2	g3775.t1 g3	1	CORT_0B02910	1	CPAR2_502650	GPI mannosyltransferase	1	1		
218	4	2	g1023.t1 g1	1	CORT_0H00580	1	CPAR2_300510	ATP11	1	1		
219	4	2	g226.t1 g22	1	CORT_0A00890	1	CPAR2_800820	Protein of unknown function DUF726	1	1		
220	4	1	g5892.t1	2	CORT_0C00820	1	CPAR2_808340		0	-1		
221	4	2	g2864.t1 g2	1	CORT_0A06340	1	CPAR2_206980		1	1		
222	4	1	g3241.t1	1	CORT_0D01190	2	CPAR2_200910	Nuclear pore assembly and biogenesis protein, APQ12	-1	0		
223	4	2	g4333.t1 g4	1	CORT_0F03890	1	CPAR2_602920	Heme A synthase, type 2; Heme A synthase	1	1		
224	4	1	g4739.t1	1	CORT_0A04320	2	CPAR2_804230	Alkaline-phosphatase-like, core domain; Alkaline phosphatase-like, alpha/beta/alpha; Alkaline phosphatase; Alkaline phosphatase, active site	-1	0		
225	4	2	g4433.t1 g4	1	CORT_0A01570	1	CPAR2_801500	Major facilitator superfamily domain, general substrate transporter; Major facilitator superfamily	1	1		
226	4	2	g2000.t1 g4	1	CORT_0F02360	1	CPAR2_601360	DnaJ homologue, subfamily C, member 28, conserved domain	1	1		
227	4	2	g10.t1 g305	1	CORT_0F00840	1	CPAR2_100720	Metallophosphoesterase domain	1	1		
228	4	2	g1818.t1 g1	1	CORT_0C04090	1	CPAR2_404660	Sin3 binding protein	1	1		
229	4	1	g2661.t1	1	CORT_0A08510	2	CPAR2_209220	6-phosphofructo-2-kinase; Fructose-2,6-bisphosphatase	-1	0		
230	4	2	g4352.t1 g4	1	CORT_0F04060	1	CPAR2_603090	SUN	1	1		
231	4	2	g2944.t1 g3	1	CORT_0A05730	1	CPAR2_206380	Nucleic acid-binding, OB-fold; Nucleic acid-binding, OB-fold-like; Tetratricopeptide repeat-containing domain; RNA-processing protein, HAT heli	1	1		
232	4	2	g4542.t1 g4	1	CORT_0A02400	1	CPAR2_802350		1	1		
233	4	2	g4885.t1 g4	1	CORT_0B03190	1	CPAR2_101900	Helicase, C-terminal; SNF2-related; Helicase, superfamily 1/2, ATP-binding domain	1	1		
234	4	2	g2501.t1 g2	1	CORT_0A09880	1	CPAR2_210550	Transcriptional adaptor 2; Zinc finger, ZZ-type; Homeodomain-like; SANT/Myb domain; SWIRM domain; SANT domain	1	1		
235	4	1	g3960.t1	2	CORT_0B00920	1	CPAR2_500770	Small GTP-binding protein domain; Small GTPase superfamily, Rho type; Small GTPase superfamily, Rab type; Small GTPase superfamily, Ra	0	-1		
236	4	1	g5558.t1	1	CORT_0B09870	2	CPAR2_108150	Monooxygenase, FAD-binding; Ubiquinone biosynthesis hydroxylase, UbiH/UbiF/VisC/COQ6, conserved site; Ubiquinone biosynthesis hydroxyl	-1	0		
237	4	1	g5043.t1	1	CORT_0E04800	2	CPAR2_100750	Peptidase aspartic, catalytic; Peptidase aspartic; Peptidase A1	-1	0		
238	4	2	g3180.t1 g3	1	CORT_0D00650	1	CPAR2_200570	Ribosomal RNA large subunit methyltransferase E; Ribosomal RNA methyltransferase FtsJ domain	1	1		
239	4	2	g2663.t1 g2	1	CORT_0A08490	1	CPAR2_209190	RmlC-like cupin domain; Acireductone dioxygenase ARD family; RmlC-like jelly roll fold	1	1		
240	4	1	g3857.t1	1	CORT_0B01960	2	CPAR2_501800	Protein of unknown function DUF3112	-1	0		
241	4	2	g5405.t1 g5	1	CORT_0B08330	1	CPAR2_107130	WD40/YVTN repeat-like-containing domain; WD40 repeat; WD40-repeat-containing domain; BOP1, N-terminal domain; WD40 repeat, conserve	1	1		
242	4	1	g5147.t1	1	CORT_0B05695	2	CPAR2_104510 CPAR2_104640		-1	0		
243	4	1	g3155.t1	2	CORT_0C05110	1	CPAR2_200220	Major facilitator superfamily domain, general substrate transporter; Major facilitator superfamily	0	-1		
244	4	2	g177.t1 g17	1	CORT_0E03810	1	CPAR2_403800	Exoribonuclease, phosphorolytic domain 1; Exoribonuclease, phosphorolytic domain 2; Ribosomal protein S5 domain 2-type fold	1	1		
245	4	2	g4105.t1 g4	1	CORT_0H02190	1	CPAR2_600610	CRAL-TRIO domain; CRAL/TRIO, N-terminal domain	1	1		
246	4	2	g1158.t1 g1	1	CORT_0E05730	1	CPAR2_303580	Phosphatidylinositol 4-kinase; Phosphatidylinositol Kinase; Armadillo-type fold; Phosphoinositide 3-kinase, accessory (PIK) domain; Phosphatid	1	1		
247	4	1	g3679.t1	1	CORT_0D05690	2	CPAR2_205560	Crotonase, core	-1	0		
248	4	2	g2140.t1 g2	1	CORT_0C07290	1	CPAR2_407940	Armadillo-type fold; Domain of unknown function DUF3554; HEAT, type 2; Armadillo-like helical; Proteasome component ECM29/Translational	1	1		
249	4	1	g2575.t1	2	CORT_0A09400	1	CPAR2_210120	Ferredoxin reductase-type FAD-binding domain; Ferric reductase transmembrane component-like domain; FAD-binding 8; Ferric reductase, NA	0	-1		
250	4	2	g3606.t1 g3	1	CORT_0D04960	1	CPAR2_204850	ATPase domain, prokaryote; Kinesin, motor domain	1	1		
251	4	2	g178.t1 g18	1	CORT_0E03820	1	CPAR2_403810	Armadillo-type fold; tRNA/rRNA methyltransferase, SpoU	1	1		
252	4	2	g4784.t1 g4	1	CORT_0A04780	1	CPAR2_804770	Pleckstrin homology-like domain; Pleckstrin homology domain; Oxysterol-binding protein; Oxysterol-binding protein, conserved site; Ankyrin rep	1	1		
253	4	2	g3370.t1 g3	1	CORT_0D02530	1	CPAR2_202460		1	1		
254	4	2	g5721.t1 g5	1	CORT_0C02620	1	CPAR2_806530	DNA mismatch repair protein MutS-like, N-terminal; DNA mismatch repair protein MutS, core; DNA mismatch repair protein MutS, connector; D	1	1		
255	4	2	g3503.t1 g3	1	CORT_0D03830	1	CPAR2_203720	ATPase, P-type, K/Mg/Cd/Cu/Zn/Na/Ca/Na/H-transporter; ATPase, P-type phosphorylation site; ATPase, P-type, cytoplasmic domain N; ATPas	1	1		
256	4	2	g2913.t1 g2	1	CORT_0A05880	1	CPAR2_206520	WD40/YVTN repeat-like-containing domain; Nucleoporin, Nup133/Nup155-like, N-terminal; Nucleoporin, Nup133/Nup155-like, C-terminal	1	1		
257	4	1	g5569.t1	2	CORT_0B10010	1	CPAR2_108910	D-amino-acid oxidase; NAD(P)-binding domain; FAD dependent oxidoreductase	0	-1		
258	4	2	g30.t1 g31.t	1	CORT_0A05670	1	CPAR2_206320	ATPase, P-type, K/Mg/Cd/Cu/Zn/Na/Ca/Na/H-transporter; ATPase, P-type, cytoplasmic domain N; ATPase, P-type, unknown pump specificity (t	1	1		
259	4	2	g5641.t1 g5	1	CORT_0E04020	1	CPAR2_301930	Armadillo-type fold; HEAT, type 2; Armadillo-like helical	1	1		

260	4	1	g3779.t1	2	CORT_0B02720	1	CPAR2_502520	Membrane insertase OXA1/ALB3/YidC	0	-1	
261	4	2	g364.t1 g36	1	CORT_0E03460	1	CPAR2_403550	Gamma-glutamyltranspeptidase	1	1	
262	4	2	g1339.t1 g1	1	CORT_0G02250	1	CPAR2_702100	Taurine catabolism dioxygenase TauD/TfdA	1	1	
263	4	1	g1240.t1	1	CORT_0E04930	2	CPAR2_302770	Steryl acetyl hydrolase; Lipase, GDXG, active site	-1	0	
264	4	1	g1870.t1	2	CORT_0C04550	1	CPAR2_405150	Proteasome Inhibitor PI31; PI31 proteasome regulator	0	-1	
265	4	2	g2500.t1 g2	1	CORT_0A09870	1	CPAR2_210540	Ribosomal protein S17e, conserved site; Ribosomal protein S17e	1	1	
266	4	2	g3764.t1 g3	1	CORT_0B02790	1	CPAR2_502750	Chaperone, tailless complex polypeptide 1; T-complex protein 1, gamma subunit; Chaperonin TCP-1, conserved site; Chaperonin Cpn60/TCP-1	1	1	
267	4	2	g575.t1 g59	1	CORT_0E01400	1	CPAR2_401350	Barwin-related endoglucanase; Barwin-like endoglucanase	1	1	
268	4	2	g2865.t1 g2	1	CORT_0A06350	1	CPAR2_206990	Cytochrome P450, E-class, CYP52; Cytochrome P450, E-class, group II; Cytochrome P450; Cytochrome P450, conserved site	1	1	
269	4	2	g3399.t1 g3	1	CORT_0D02830	1	CPAR2_202750	Spa2 homology (SHD) of GIT	1	1	
270	4	2	g3615.t1 g3	1	CORT_0D05050	1	CPAR2_204940	Cation efflux protein	1	1	
271	4	2	g5534.t1 g5	1	CORT_0B09630	1	CPAR2_108540	Phox-associated domain	1	1	
272	4	2	g5651.t1 g5	1	CORT_0C03300	1	CPAR2_805850	SNF5/SMARCB1/INI1	1	1	
273	4	1	g3339.t1	2	CORT_0D02080	1	CPAR2_202130	Major facilitator superfamily domain, general substrate transporter; Major facilitator superfamily; Major facilitator superfamily domain	0	-1	
274	4	2	g4913.t1 g4	1	CORT_0B03490	1	CPAR2_102200	Ribonuclease III domain	1	1	
275	4	1	g1017.t1	1	CORT_0H00640	2	CPAR2_300570	TM1410 hypothetical-related protein; Glycoside hydrolase, superfamily	-1	0	
276	4	2	g3759.t1 g3	1	CORT_0F01710	1	CPAR2_502630	C2 calcium/lipid-binding domain, CaLB; Phosphatidylserine decarboxylase-related; C2 calcium-dependent membrane targeting; Phosphatidylse	1	1	
277	4	1	g349.t1	1	CORT_0E03940	2	CPAR2_301830	Acyl-CoA N-acyltransferase; GNAT domain	-1	0	
278	4	1	g1385.t1	2	CORT_0G01730	1	CPAR2_701590	Peptidase S10, serine carboxypeptidase, active site; Peptidase S10, serine carboxypeptidase	0	-1	
279	4	1	g2009.t1	2	CORT_0C05960	1	CPAR2_406550	Polyketide synthase, enoylreductase; Alcohol dehydrogenase superfamily, zinc-type; Alcohol dehydrogenase GroES-like; Alcohol dehydrogena	0	-1	
280	4	2	g452.t1 g45	1	CORT_0E02720	1	CPAR2_402660	Nucleoporin Nup120/160	1	1	
281	4	1	g2443.t1	1	CORT_0A10620	2	CPAR2_211230	Polyketide synthase, enoylreductase; Alcohol dehydrogenase superfamily, zinc-type; NAD(P)-binding domain; GroES-like; Alcohol dehydrogen	-1	0	
282	4	1	g1625.t1	1	CORT_0G03670	2	CPAR2_703480	Regulatory subunit Dfp1/Him1, central region; Zinc finger, DBF-type	-1	0	
283	4	2	g93.t1 g94.t	1	CORT_0B11350	1	CPAR2_110240	Hid-1	1	1	
284	4	2	g2542.t1 g2	1	CORT_0A09740	1	CPAR2_210420	Globin; Globin-like; Globin, structural domain	1	1	
285	4	2	g4109.t1 g4	1	CORT_0H02160	1	CPAR2_600650	Peptidase M16, core; Metalloenzyme, LuxS/M16 peptidase-like, metal-binding; Peptidase M16, N-terminal; Peptidase M16, C-terminal	1	1	
286	4	1	g320.t1	1	CORT_0A00120	2	CPAR2_100010	Bul1, N-terminal; Bul1, C-terminal	-1	0	
287	4	2	g87.t1 g88.t	1	CORT_0B11400	1	CPAR2_110290	Ribosomal protein S3Ae; Ribosomal protein S3Ae, conserved site	1	1	
288	4	2	g3110.t1 g3	1	CORT_0F00290	1	CPAR2_100180	WD40/YVTN repeat-like-containing domain; WD40-repeat-containing domain; WD40 repeat	1	1	
289	4	2	g2763.t1 g5	1	CORT_0B07830	1	CPAR2_106580	LicD	1	1	
290	4	2	g3415.t1 g3	1	CORT_0D02990	1	CPAR2_202910	5'-3' exoribonuclease 1; Putative 5-3 exonuclease	1	1	
291	4	2	g4415.t1 g4	1	CORT_0F04650	1	CPAR2_603690	Na+/H+ exchanger; Cation/H+ exchanger, CPA1 family; Cation/H+ exchanger	1	1	
292	4	2	g4544.t1 g4	1	CORT_0A02420	1	CPAR2_802370	Target SNARE coiled-coil domain	1	1	
293	4	1	g5565.t1	1	CORT_0B09950	2	CPAR2_108860	Major facilitator superfamily domain, general substrate transporter; Major facilitator superfamily; Major facilitator superfamily domain	-1	0	
294	4	2	g3865.t1 g3	1	CORT_0B01870	1	CPAR2_501710	ABC transporter-like; HEAT, type 2; ABC transporter, conserved site; Armadillo-like helical; Armadillo-type fold; Elongation Factor 3; AAA+ ATP	1	1	
295	4	2	g3736.t1 g3	1	CORT_0B02930	1	CPAR2_101770	Pleckstrin homology-like domain	1	1	
296	4	2	g595.t1 g59	1	CORT_0E01350	1	CPAR2_401300	Acyltransferase ChoActase/COT/CPT	1	1	
297	4	2	g3234.t1 g3	1	CORT_0D01020	1	CPAR2_200970	Major facilitator superfamily domain, general substrate transporter; Major facilitator superfamily; Tetracycline resistance protein TetB/drug resist	1	1	
298	4	1	g2035.t1	1	CORT_0C06210	2	CPAR2_406800	Peptidase S8, subtilisin, Ser-active site; Peptidase S8/S53, subtilisin/kexin/sedolisin; Proteinase inhibitor, propeptide; Peptidase S8, subtilisin, H	-1	0	
299	4	2	g1539.t1 g3	1	CORT_0A00130	1	CPAR2_800020	Thiolase, conserved site; Thiolase-like, subgroup; Thiolase, active site; Thiolase-like; Thiolase, C-terminal; Thiolase; Thiolase, N-terminal; Thiol	1	1	
300	4	2	g3098.t1 g3	1	CORT_0F00430	1	CPAR2_100330	Major facilitator superfamily domain, general substrate transporter; Major facilitator superfamily	1	1	
301	4	2	g2936.t1 g2	1	CORT_0A05790	1	CPAR2_206430	Ribosomal protein S35, mitochondrial	1	1	
302	4	1	g1442.t1	1	CORT_0G01100	2	CPAR2_700940	Alternative oxidase	-1	0	
303	4	1	g4225.t1	1	CORT_0F02920	2	CPAR2_601840	Polyketide synthase, enoylreductase; Alcohol dehydrogenase superfamily, zinc-type; GroES-like; Alcohol dehydrogenase GroES-like; Alcohol d	-1	0	
304	4	2	g2498.t1 g4	1	CORT_0A09850	1	CPAR2_210520	RNA-processing protein, HAT helix; Tetratricopeptide-like helical; U3 small nucleolar RNA-associated protein 6	1	1	
305	4	2	g550.t1 g55	1	CORT_0E01790	1	CPAR2_401740	Ferredoxin reductase-type FAD-binding domain; Riboflavin synthase-like beta-barrel; Ferric reductase transmembrane component-like domain;	1	1	
306	4	2	g4312.t1 g4	1	CORT_0F03710	1	CPAR2_602730	Peptidase aspartic, catalytic; Peptidase aspartic; Peptidase aspartic, active site; Peptidase A1	1	1	
307	4	2	g5103.t1 g5	1	CORT_0B05330	1	CPAR2_104120	WD40/YVTN repeat-like-containing domain; WD40 repeat; Histone-binding protein RBBP4; WD40-repeat-containing domain	1	1	
308	4	2	g3371.t1 g3	1	CORT_0D02540	1	CPAR2_202470	Tetratricopeptide-like helical	1	1	
309	4	2	g2977.t1 g2	1	CORT_0F01600	1	CPAR2_101510		1	1	
310	4	2	g3307.t1 g3	1	CORT_0D01860	1	CPAR2_201800	Bul1, N-terminal; Bul1, C-terminal	1	1	
311	4	1	g4929.t1	2	CORT_0A13080	1	CPAR2_809010		0	-1	

312	4	2	g5369.t1 g5	1	CORT_0B07990	1	CPAR2_106760	Cysteine synthase/cystathionine beta-synthase P-phosphate-binding site; Cystathionine beta-synthase, core; Cystathionine beta-synthase; Tryp	1	1		
313	4	1	g1621.t1	1	CORT_0G03880	2	CPAR2_703670	Metallophosphoesterase domain	-1	0		
314	4	2	g2689.t1 g2	1	CORT_0A08250	1	CPAR2_208980	Six-hairpin glycosidase; Six-hairpin glycosidase-like; Glycoside hydrolase, family 65, N-terminal; Glycoside hydrolase-type carbohydrate-binding	1	1		
315	4	2	g1247.t1 g1	1	CORT_0E04830	1	CPAR2_302650	Acyl transferase/acyl hydrolase/lysophospholipase; Acyl transferase; Fatty acid synthase; Domain of unknown function DUF1729; Fatty acid sy	1	1		
316	4	2	g3034.t1 g6	1	CORT_0F01050	1	CPAR2_100970	HAD-like domain; Pyrimidine 5-nucleotidase	1	1		
317	4	1	g5837.t1	2	CORT_0C01380	1	CPAR2_807750		0	-1		
318	4	2	g1030.t1 g1	1	CORT_0H00520	1	CPAR2_300450	Folylpolyglutamate synthetase, conserved site; Mur ligase, C-terminal; Folylpolyglutamate synthetase; Mur ligase, central	1	1		
319	4	1	g2515.t1	2	CORT_0A09980	1	CPAR2_210650	Phospholipid/glycerol acyltransferase	0	-1		
320	4	2	g20.t1 g422	1	CORT_0F02890	1	CPAR2_601925		1	1		
321	4	2	g1345.t1 g1	1	CORT_0G02170	1	CPAR2_702040	Peptidase aspartic, catalytic; Peptidase aspartic; Peptidase A1	1	1		
322	4	2	g554.t1 g55	1	CORT_0E01750	1	CPAR2_401700	Alpha/gamma-adaptin-binding protein p34	1	1		
323	4	2	g1119.t1 g2	1	CORT_0A12880	1	CPAR2_213550	Arginine-tRNA-protein transferase, C-terminal; Acyl-CoA N-acyltransferase; Arginine-tRNA-protein transferase 1, eukaryotic; Arginine-tRNA-prot	1	1		
324	4	2	g2226.t1 g2	1	CORT_0A12810	1	CPAR2_213500	Zinc finger, RING-type; Zinc finger, RING/FYVE/PHD-type; Zinc finger, CCCH-type	1	1		
325	4	1	g2660.t1	1	CORT_0A08520	2	CPAR2_209230	Heat shock protein DnaJ, N-terminal	-1	0		
326	4	2	g4545.t1 g4	1	CORT_0A02430	1	CPAR2_802380	GRIP	1	1		
327	4	2	g1386.t1 g3	1	CORT_0D04740	1	CPAR2_204630	CheY-like superfamily; Signal transduction response regulator, receiver domain	1	1		
328	4	2	g3744.t1 g5	1	CORT_0B03010	1	CPAR2_101690	ATPase, AAA-type, core; AAA+ ATPase domain; ATPase, AAA-type, conserved site	1	1		
329	4	2	g2693.t1 g2	1	CORT_0A08220	1	CPAR2_208950		1	1		
330	4	1	g3750.t1	1	CORT_0A05590	2	CPAR2_101630	Uracil-DNA glycosylase-like; DNA glycosylase, G/T mismatch	-1	0		
331	4	2	g1115.t1 g1	1	CORT_0E06190	1	CPAR2_304010	Ras GTPase-activating protein; C2 membrane targeting protein; C2 calcium/lipid-binding domain, CaLB; Ras GTPase-activating protein, conser	1	1		
332	4	2	g4519.t1 g4	1	CORT_0A02190	1	CPAR2_802140	GTP binding domain; GTP-binding protein, orthogonal bundle domain	1	1		
333	4	2	g1831.t1 g1	1	CORT_0C04180	1	CPAR2_404740		1	1		
334	4	2	g5636.t1 g5	1	CORT_0E04060	1	CPAR2_301970	Inositol-pentakisphosphate 2-kinase	1	1		
335	4	2	g460.t1 g46	1	CORT_0E02640	1	CPAR2_402580	ATPase domain, prokaryote	1	1		
336	4	1	g5003.t2	1	CORT_0B04390	2	CPAR2_103160	ABC transporter, transmembrane domain; ABC transporter-like; ABC transporter, conserved site; ABC transporter, integral membrane type 1; A	-1	0		
337	4	1	g4411.t1	2	CORT_0F04600	1	CPAR2_603630	Like-Sm (LSM) domain; Rad52/22 double-strand break repair protein; Ribonucleoprotein LSM domain, eukaryotic/archaea-type; Ribonucleoprot	0	-1		
338	4	1	g4700.t1	2	CORT_0A03880	1	CPAR2_803860	Src homology-3 domain; Phox homologous domain	0	-1		
339	4	2	g3035.t1 g5	1	CORT_0F01040	1	CPAR2_100960		1	1		
340	4	2	g4635.t1 g4	1	CORT_0A03260	1	CPAR2_803210	Armadillo-type fold	1	1		
341	4	2	g3055.t1 g8	1	CORT_0F00860	1	CPAR2_100740	Cyclin, N-terminal; Cyclin-like; Cyclin PHO80-like	1	1		
342	4	2	g5127.t2 g5	1	CORT_0B05580	1	CPAR2_104340	Zinc finger, PHD-type; Zinc finger, RING-type; Zinc finger, FYVE/PHD-type; Zinc finger, RING/FYVE/PHD-type; Zinc finger, PHD-finger; Zinc fin	1	1		
343	4	2	g4521.t1 g4	1	CORT_0A02200	1	CPAR2_802150	Chromatin associated protein KTI12	1	1		
344	4	1	g5822.t1	2	CORT_0C01510	1	CPAR2_807620	FAR-17a/AIG1-like protein	0	-1		
345	4	2	g4434.t1 g4	1	CORT_0A01560	1	CPAR2_801490	Major facilitator superfamily domain, general substrate transporter; Major facilitator superfamily	1	1		
346	4	2	g5049.t1 g5	1	CORT_0B04810	1	CPAR2_103610	Drug/metabolite transporter	1	1		
347	4	1	g4591.t1	1	CORT_0A02810	2	CPAR2_100860	WD40-repeat-containing domain; F-box domain, cyclin-like; WD40 repeat, conserved site; WD40/YVTN repeat-like-containing domain; G-protei	-1	0		
348	4	1	g5150.t1	1	CORT_0B05760	2	CPAR2_104540	NAD(P)-binding domain; NAD-dependent epimerase/dehydratase	-1	0		
349	4	1	g2525.t1	2	CORT_0A10050	1	CPAR2_210750	Major facilitator superfamily domain, general substrate transporter; Major facilitator superfamily; Major facilitator superfamily domain	0	-1		
350	4	2	g2502.t1 g2	1	CORT_0A09890	1	CPAR2_210560		1	1		
351	4	1	g4934.t1	1	CORT_0B03820	2	CPAR2_102420	Peptidase A1; Peptidase aspartic; Peptidase aspartic, active site; Peptidase aspartic, catalytic	-1	0		
352	4	2	g5134.t1 g5	1	CORT_0B05650	1	CPAR2_104420	Guanine-nucleotide dissociation stimulator CDC25; Ras guanine nucleotide exchange factor, domain; Ras guanine-nucleotide exchange factor,	1	1		
353	4	1	g668.t1	1	CORT_0E00580	2	CPAR2_400430	Clathrin light chain	-1	0		
354	4	2	g2388.t1 g2	1	CORT_0A11230	1	CPAR2_211890	Domain of unknown function DUF1720; WH2 domain; Actin cytoskeleton-regulatory complex protein PAN1; EF-hand-like domain; EPS15 homol	1	1		
355	4	2	g4902.t1 g4	1	CORT_0B03390	1	CPAR2_102080	Fatty acid/sphingolipid desaturase; Cytochrome b5; Fatty acid desaturase, type 1	1	1		
356	4	2	g1744.t1 g1	1	CORT_0G02440	1	CPAR2_702250	CheY-like superfamily; AGC-kinase, C-terminal; Signal transduction response regulator, receiver domain; Serine/threonine-protein kinase, activ	1	1		
357	4	2	g2908.t1 g2	1	CORT_0A05980	1	CPAR2_206630	Alcohol dehydrogenase superfamily, zinc-type; Alcohol dehydrogenase, C-terminal; NAD(P)-binding domain; GroES-like	1	1		
358	4	1	g2940.t1	1	CORT_0A05760	2	CPAR2_206500	Protein of unknown function DUF3445	-1	0		
359	4	1	g685.t1	1	CORT_0E00440	2	CPAR2_400270	Zinc finger, HIT-type	-1	0		
360	4	1	g1090.t1	1	CORT_0E06470	2	CPAR2_100480	NAD(P)-binding domain; NAD-dependent epimerase/dehydratase	-1	0		
361	4	2	g5682.t1 g5	1	CORT_0C02970	1	CPAR2_806170	Zinc finger, DNA-directed DNA polymerase, family B, alpha; DNA-directed DNA polymerase, family B, exonuclease domain; DNA polymerase, p	1	1		
362	4	2	g27.t1 g311	1	CORT_0F00130	1	CPAR2_100030	BAR domain; Src homology-3 domain	1	1		
363	4	2	g2675.t1 g2	1	CORT_0A08380	1	CPAR2_209100		1	1		

364	4	2	g3124.t1 g4	1	CORT_0F00220	1	CPAR2_100110	Glycoside hydrolase, superfamily; Glucanosyltransferase; Glycoside hydrolase, catalytic domain	1	1		
365	4	1	g1752.t1	1	CORT_0C03500	2	CPAR2_404060	Cell wall beta-glucan synthesis; Yeast cell wall synthesis KRE9/KNH1	-1	0		
366	4	2	g4756.t1 g4	1	CORT_0A04510	1	CPAR2_804520	6-phosphogluconate dehydrogenase, C-terminal-like; Ketopantoate reductase ApbA/PanE, C-terminal; Dehydrogenase, multihelical	1	1		
367	4	1	g5232.t1	2	CORT_0B06470	1	CPAR2_105310	Peptidyl-prolyl cis-trans isomerase, PpiC-type; WW/Rsp5/WWP	0	-1		
368	4	2	g4296.t1 g4	1	CORT_0F03540	1	CPAR2_602570	FMP27, GFWDK domain; FMP27, SW domain; FMP27, domain of unknown function DUF2405; FMP27, N-terminal; FMP27, WPPW domain; F	1	1		
369	4	2	g75.t1 g77.t	1	CORT_0B11500	1	CPAR2_110390	Domain of unknown function DUF1708	1	1		
370	4	2	g4672.t1 g4	1	CORT_0A03630	1	CPAR2_803610		1	1		
371	4	2	g1631.t1 g1	1	CORT_0G03620	1	CPAR2_703450	Domain of unknown function DUF1212; Domain of unknown function DUF3815	1	1		
372	4	2	g5888.t1 g5	1	CORT_0C00860	1	CPAR2_808290	GTP binding domain	1	1		
373	4	2	g5126.t1 g5	1	CORT_0B05570	1	CPAR2_104330	Actin-binding, cofilin/tropomyosin type	1	1		
374	4	1	g5498.t1	1	CORT_0B09310	2	CPAR2_108170	Ribonuclease P, Rpm2, mitochondrial	-1	0		
375	4	1	g3907.t1	1	CORT_0B01440	2	CPAR2_501280	Zn(2)-C6 fungal-type DNA-binding domain	-1	0		
376	4	2	g2072.t1 g2	1	CORT_0C06590	1	CPAR2_407220	Oxoglutarate/iron-dependent dioxygenase; Non-haem dioxygenase N-terminal domain	1	1		
377	4	2	g2383.t1 g2	1	CORT_0A11270	1	CPAR2_211930	Zinc finger, FYVE/PHD-type; Chaperonin Cpn60/TCP-1; Zinc finger, RING/FYVE/PHD-type; Zinc finger, FYVE-type; Phosphatidylinositol-4-pho	1	1		
378	4	1	g3884.t1	1	CORT_0A07490	2	CPAR2_208160	Phosphate permease; Major facilitator superfamily domain, general substrate transporter; General substrate transporter; Major facilitator superf	-1	0		
379	4	2	g3426.t1 g3	1	CORT_0D03090	1	CPAR2_203010	Kinesin, motor domain; Kinesin, motor region, conserved site	1	1		
380	4	2	g4165.t1 g4	1	CORT_0F02270	1	CPAR2_601270		1	1		
381	4	2	g4276.t1 g4	1	CORT_0F03370	1	CPAR2_602390	Aldolase-type TIM barrel; NADH:flavin oxidoreductase/NADH oxidase, N-terminal	1	1		
382	4	2	g236.t1 g23	1	CORT_0A00980	1	CPAR2_800910		1	1		
383	4	2	g4386.t1 g4	1	CORT_0F04370	1	CPAR2_603410		1	1		
384	4	2	g2347.t1 g2	1	CORT_0A11620	1	CPAR2_212290	Exportin/Importin, Cse1-like; Armadillo-type fold; Armadillo-like helical; Importin-beta, N-terminal	1	1		
385	4	1	g4838.t1	1	CORT_0A05290	2	CPAR2_301640	Major facilitator superfamily domain, general substrate transporter; Major facilitator superfamily; Major facilitator superfamily domain	-1	0		
386	4	2	g2984.t1 g2	1	CORT_0F01460	1	CPAR2_101370	Rab-GTPase-TBC domain	1	1		
387	4	2	g4543.t1 g4	1	CORT_0A02410	1	CPAR2_802360	Vacuolar protein sorting 55	1	1		
388	4	2	g2420.t1 g2	1	CORT_0A10920	1	CPAR2_211580	Mitochondrial distribution and morphology family 33, fungi	1	1		
389	4	2	g1950.t1 g1	1	CORT_0C05390	1	CPAR2_405990		1	1		
390	4	1	g5497.t1	1	CORT_0B09300	2	CPAR2_108130	WD40/YVTN repeat-like-containing domain; Ubiquitin interacting motif; Quinonprotein alcohol dehydrogenase-like; WD40-repeat-containing do	-1	0		
391	4	2	g5040.t1 g5	1	CORT_0B04750	1	CPAR2_103540		1	1		
392	4	2	g3272.t1 g3	1	CORT_0D01490	1	CPAR2_201440	Major facilitator superfamily domain, general substrate transporter; Major facilitator superfamily; Major facilitator superfamily domain	1	1		
393	4	2	g2002.t1 g2	1	CORT_0C05580	1	CPAR2_406180	Aldehyde dehydrogenase, N-terminal; Aldehyde dehydrogenase, conserved site; Aldehyde/histidinol dehydrogenase; Aldehyde dehydrogenase	1	1		
394	4	2	g1833.t1 g5	1	CORT_0C01970	1	CPAR2_807180	Flavin monooxygenase-like; NAD(P)-binding domain	1	1		
395	4	2	g3899.t1 g3	1	CORT_0B01530	1	CPAR2_501390	Tryptophan synthase beta chain/beta chain-like; Tryptophan synthase, alpha chain, active site; Aldolase-type TIM barrel; Tryptophan synthase,	1	1		
396	4	2	g4531.t1 g4	1	CORT_0A02290	1	CPAR2_802240	Major facilitator superfamily domain, general substrate transporter; Major facilitator superfamily; Major facilitator superfamily domain	1	1		
397	4	1	g5504.t1	1	CORT_0B09370	2	CPAR2_108230	Rhodopsin, archaeal/bacterial/fungal	-1	0		
398	4	1	g2799.t1	2	CORT_0A07110	1	CPAR2_207760	LicD	0	-1		
399	4	2	g2270.t1 g2	1	CORT_0A12410	1	CPAR2_213100	Zinc finger, C2H2	1	1		
400	4	2	g2499.t1 g2	1	CORT_0A09860	1	CPAR2_210530		1	1		
401	10	9	g1775.t1 g2	1	CORT_0B02750				9	8		
402	5	3	g5971.t1 g8	2	CORT_0D06960 CORT_0E04360				3	1		
403	3	2	g2225.t1 g2	1	CORT_0A12820				2	1		
404	3	2	g4427.t1 g7	1	CORT_0F04750				2	1		
405	3	1	g1344.t1	2	CORT_0F00880 CORT_0G02030			Peptidase aspartic, catalytic; Peptidase aspartic; Peptidase A1	1	-1		
406	3	2	g3999.t1 g6	1	CORT_0B00510			Leucine-rich repeat	2	1		
407	2	1	g2342.t1	1	CORT_0A11670			CENP-A-nucleosome distal centromere subunit CENP-Q	1	0		
408	2	1	g1215.t1	1	CORT_0E05130				1	0		
409	2	1	g1236.t1	1	CORT_0E04900			Steryl acetyl hydrolase	1	0		
410	2	1	g2962.t1	1	CORT_0F01800				1	0		
411	2	1	g2951.t1	1	CORT_0A05640				1	0		
412	2	1	g2916.t1	1	CORT_0A05900				1	0		
413	2	1	g1866.t1	1	CORT_0C04515				1	0		
414	2	1	g1257.t1	1	CORT_0E04740			Bacterial transferase hexapeptide repeat; Trimeric LpxA-like; Hexapeptide transferase, conserved site	1	0		
415	2	1	g3731.t1	1	CORT_0D06210			Chromosome segregation protein Spc25	1	0		

416	2	1	g4559.t1	1	CORT_0A02500				1	0	
417	2	1	g4912.t1	1	CORT_0B03480			Nucleolar protein Dnt1-like, N-terminal	1	0	
418	2	1	g5908.t1	1	CORT_0G04050			F-box domain, cyclin-like	1	0	
419	2	1	g4175.t1	1	CORT_0E04090			F-box domain, cyclin-like	1	0	
420	2	1	g2022.t1	1	CORT_0C06090			Leucine-rich repeat; Leucine-rich repeat, typical subtype; Leucine rich repeat 4	1	0	
421	2	1	g3930.t1	1	CORT_0B01240				1	0	
422	2	1	g2242.t1	1	CORT_0A12680			Peptidase A1; Peptidase aspartic; Peptidase aspartic, catalytic	1	0	
423	2	1	g732.t1	1	CORT_0A00110			Leucine-rich repeat; Leucine-rich repeat, typical subtype; Leucine rich repeat 4	1	0	
424	2	1	g5956.t1	1	CORT_0C00240				1	0	
425	2	1	g5282.t1	1	CORT_0E04080				1	0	
426	2	1	g2281.t1	1	CORT_0A12300				1	0	
427	2	1	g1827.t1	1	CORT_0C04150				1	0	
428	2	1	g3801.t1	1	CORT_0B02530				1	0	
429	2	1	g5949.t2	1	CORT_0C00305			Leucine-rich repeat	1	0	
430	7	6	g1077.t1 g11.t1 g2153.t1 g4431.t1			1	CPAR2_704330	Major facilitator superfamily domain, general substrate transporter; Major facilitator superfamily; Major facilitator superfamily domain	5	6	
431	7	6	g1078.t1 g2152.t1 g4039.t1 g4430.			1	CPAR2_704340	RmlC-like cupin domain; RmlC-like jelly roll fold; Cupin 2, conserved barrel	5	6	
432	7	6	g1079.t1 g2151.t1 g4038.t1 g4429.			1	CPAR2_704350	Fumarylacetoacetase, C-terminal-related; Fumarylacetoacetase, C-terminal	5	6	
433	6	5	g1080.t1 g2150.t1 g4428.t1 g721.t			1	CPAR2_704360	Mss4-like; Glutathione-dependent formaldehyde-activating enzyme/centromere protein V	4	5	
434	4	3	g2497.t1 g4667.t1 g4675.t1			1	CPAR2_807120		2	3	
435	4	2	g280.t1 g281.t1			2	CPAR2_101750 CPAR2_105740		0	2	
436	4	2	g3538.t1 g3539.t1			2	CPAR2_204210	Cytochrome P450, E-class, CYP52; Cytochrome P450, E-class, group II; Cytochrome P450; Cytochrome P450, conserved site	0	2	
437	4	2	g3319.t1 g3324.t1			2	CPAR2_201910 CPAR2_201960		0	2	
438	4	2	g5363.t1 g5364.t1			2	CPAR2_106690	Amino acid permease, conserved site; Amino acid permease domain; Amino acid/polyamine transporter I	0	2	
439	4	2	g344.t1 g345.t1			2	CPAR2_301850	Thioredoxin-like fold; UAS; UBX	0	2	
440	4	2	g3042.t1 g3051.t1			2	CPAR2_100850	RNA recognition motif domain; Nucleotide-binding, alpha-beta plait	0	2	
441	4	2	g4483.t1 g4489.t1			2	CPAR2_801770	Domain of unknown function DUF4149	0	2	
442	4	2	g585.t1 g589.t1			2	CPAR2_401460	Thioredoxin-like fold; Glutathione S-transferase/chloride channel, C-terminal; Glutathione S-transferase, C-terminal; Glutathione S-transferase,	0	2	
443	4	2	g2117.t1 g2868.t1			2	CPAR2_207060	Translation elongation/initiation factor/Ribosomal, beta-barrel; Translation elongation factor EF1A, eukaryotic/archaeal; Elongation factor, GTP-	0	2	
444	4	2	g1690.t1 g1701.t1			2	CPAR2_702760	Cytochrome C oxidase subunit II, transmembrane domain	0	2	
445	4	2	g697.t1 g704.t1			2	CPAR2_400160	Major facilitator superfamily domain, general substrate transporter; Major facilitator superfamily; Major facilitator superfamily domain	0	2	
446	3	1	g1361.t1			2	CPAR2_701870	Peptidase aspartic, catalytic; Peptidase aspartic; Peptidase A1	-1	1	
447	3	2	g4088.t1 g4163.t1			1	CPAR2_806420	Hyphally-regulated cell wall protein, N-terminal	1	2	
448	3	1	g4484.t1			2	CPAR2_801780 CPAR2_801830		-1	1	
449	3	2	g2909.t1 g2920.t1			1	CPAR2_206620	Alcohol dehydrogenase superfamily, zinc-type; Alcohol dehydrogenase, C-terminal; NAD(P)-binding domain; GroES-like	1	2	
450	3	2	g4369.t1 g4370.t1			1	CPAR2_603250		1	2	
451	3	2	g1249.t1 g1250.t1			1	CPAR2_302640	Peptidase A1; Peptidase aspartic; Peptidase aspartic, active site; Peptidase aspartic, catalytic	1	2	
452	3	1	g5446.t1			2	CPAR2_107350	Pirin, N-terminal domain; RmlC-like jelly roll fold; Pirin, C-terminal domain; Pirin; RmlC-like cupin domain	-1	1	
453	3	1	g2795.t1			2	CPAR2_207810 CPAR2_207860		-1	1	
454	2	1	g3827.t1			1	CPAR2_502130	Glycoside hydrolase, family 18, catalytic domain; Flocculin type 3 repeat; Glycoside hydrolase, superfamily; Glycoside hydrolase, catalytic doma	0	1	
455	2	1	g546.t1			1	CPAR2_401770	Heat shock protein DnaJ, N-terminal; Heat shock protein DnaJ; RNA recognition motif domain; Nucleotide-binding, alpha-beta plait; Heat shock	0	1	
456	2	1	g1081.t1			1	CPAR2_704370	Zn(2)-C6 fungal-type DNA-binding domain; Transcription factor, fungi	0	1	
457	2	1	g16.t1			1	CapafMp06	Cytochrome c oxidase, subunit I domain; Cytochrome c oxidase, subunit I; Cytochrome c oxidase, subunit I, copper-binding site	0	1	
458	2	1	g3604.t1			1	CPAR2_204830	Cyclin-like; Cyclin PHO80-like	0	1	
459	2	1	g15.t1			1	CapafMp10	ATPase, F0/V0 complex, subunit C; ATPase, F0 complex, subunit C; ATPase, F0 complex, subunit C, DCCD-binding site	0	1	
460	2	1	g5132.t1			1	CPAR2_104400	Glycoside hydrolase, superfamily; Glycoside hydrolase, family 5; Glycoside hydrolase, catalytic domain	0	1	
461	2	1	g1702.t1			1	CPAR2_702750	Thioredoxin-like fold; Redoxin	0	1	
462	2	1	g4108.t1			1	CPAR2_600640		0	1	
463	2	1	g376.t1			1	CPAR2_403420	Peptidase S10, serine carboxypeptidase, active site; Peptidase S10, serine carboxypeptidase	0	1	
464	2	1	g5047.t1			1	CPAR2_103590		0	1	
465	2	1	g3043.t1			1	CPAR2_100850.1		0	1	
466	2	1	g1272.t1			1	CPAR2_302450		0	1	
467	2	1	g3048.t1			1	CPAR2_100900.1		0	1	

468	2	1	g1270.t1			1	CPAR2_302470	Ribosomal protein MRP10, mitochondrial; MTCP1	0	1
469	2	1	g14.t1			1	CapafMp12	NADH:ubiquinone oxidoreductase, subunit 1, conserved site; NADH:ubiquinone oxidoreductase, subunit 1/F420H2 oxidoreductase subunit H	0	1
470	2	1	g5460.t1			1	CPAR2_107690	SMAD/FHA domain; Forkhead-associated (FHA) domain	0	1
471	2	1	g13.t1			1	CapafMp15	NADH dehydrogenase subunit 5, C-terminal; NADH:ubiquinone oxidoreductase, chain 5/L, N-terminal; NADH:ubiquinone/plastoquinone oxidore	0	1
472	2	1	g1256.t1			1	CPAR2_302580	Phosphatidic acid phosphatase type 2/haloperoxidase; Phosphatidic acid phosphatase/chloroperoxidase, N-terminal	0	1
473	2	1	g2070.t1			1	CPAR2_407200.	Zinc finger, C2H2	0	1
474	11			6	CORT_0D00770	5	CPAR2_105750	Homeodomain-like	-5	-6
475	4			2	CORT_0G01470	2	CPAR2_701300	Amine oxidase	-2	-2
476	4			1	CORT_0B03750	3	CPAR2_102490 CPAR2_102510 CPAR2_102520		-3	-1
477	4			2	CORT_0C01410	2	CPAR2_404720 CPAR2_500700		-2	-2
478	4			1	CORT_0D00100	3	CPAR2_300020 CPAR2_504210 CPAR2_603790		-3	-1
479	3			2	CORT_0B07390	1	CPAR2_106150	Sybindin-like protein; Longin-like domain	-1	-2
480	3			1	CORT_0E03030	2	CPAR2_403000	Amino acid transporter, transmembrane	-2	-1
481	3			1	CORT_0E00560	2	CPAR2_400450 CPAR2_400530		-2	-1
482	3			1	CORT_0D00600	2	CPAR2_200510	Dynactin p62	-2	-1
483	2			1	CORT_0F00360	1	CPAR2_100250		-1	-1
484	2			1	CORT_0B04530	1	CPAR2_103300		-1	-1
485	2			1	CORT_0H02200	1	CPAR2_600600		-1	-1
486	2			1	CORT_0C04470	1	CPAR2_405060		-1	-1
487	2			1	CORT_0D02950	1	CPAR2_202870		-1	-1
488	2			1	CORT_0B10620	1	CPAR2_109545		-1	-1
489	2			1	CORT_0D03780	1	CPAR2_203670	GTP binding domain	-1	-1
490	2			1	CORT_0B02230	1	CPAR2_502070		-1	-1
491	2			1	CORT_0E01140	1	CPAR2_401100		-1	-1
492	2			1	CORT_0C06170	1	CPAR2_406750	Gtr1/RagA G protein	-1	-1
493	2			1	CORT_0E05960	1	CPAR2_303800		-1	-1
494	2			1	CORT_0F04335	1	CPAR2_603375	Uncharacterised protein family YNL211C	-1	-1
495	2			1	CORT_0F00400	1	CPAR2_100290		-1	-1
496	2			1	CORT_0G00700	1	CPAR2_702530		-1	-1
497	2			1	CORT_0C02210	1	CPAR2_806960	EF-hand-like domain; EPS15 homology (EH)	-1	-1
498	2			1	CORT_0A00460	1	CPAR2_800380		-1	-1
499	2			1	CORT_0B11590	1	CPAR2_110490	Leucine-rich repeat; Leucine-rich repeat, typical subtype; Leucine rich repeat 4	-1	-1
500	2			1	CORT_0D03910	1	CPAR2_203800	Nucleotide excision repair, TFIIH, subunit TTDA	-1	-1
501	2			1	CORT_0B09070	1	CPAR2_107880	RNA recognition motif domain; Nucleotide-binding, alpha-beta plait	-1	-1
502	2			1	CORT_0A04490	1	CPAR2_804500	Adaptor protein complex, sigma subunit; AP complex, mu/sigma subunit; Longin-like domain	-1	-1
503	2			1	CORT_0A03440	1	CPAR2_803420	Ribosomal protein L39e domain; Ribosomal protein L39e, conserved site; Ribosomal protein L39e	-1	-1
504	2			1	CORT_0G01245	1	CPAR2_701090	Translation machinery associated TMA7	-1	-1
505	2			1	CORT_0E05240	1	CPAR2_303100		-1	-1
506	2			1	CORT_0F03110	1	CPAR2_602135		-1	-1
507	2			1	CORT_0B01500	1	CPAR2_501340	Cation efflux protein	-1	-1
508	2			1	CORT_0C01630	1	CPAR2_807510	Proteolipid membrane potential modulator	-1	-1
509	2			1	CORT_0D04190	1	CPAR2_204090		-1	-1
510	2			1	CORT_0B07940	1	CPAR2_106710	SET domain	-1	-1
511	2			1	CORT_0C05160	1	CPAR2_405750		-1	-1
512	2			1	CORT_0B02290	1	CPAR2_502120	Glycoside hydrolase, family 18, catalytic domain; Flocculin type 3 repeat; Glycoside hydrolase, superfamily; Glycoside hydrolase, catalytic doma	-1	-1
513	2			1	CORT_0A06200	1	CPAR2_206850	Mitochondrial outer membrane translocase complex, subunit Tom5	-1	-1
514	2			1	CORT_0F04210	1	CPAR2_603240	Ribosomal protein L1; Ribosomal protein L1, 3-layer alpha/beta-sandwich; Ribosomal protein L1, superfamily	-1	-1
515	2			1	CORT_0B07290	1	CPAR2_106060	G-patch domain	-1	-1
516	2			1	CORT_0H01110	1	CPAR2_301090	Nucleotide binding protein, PINc	-1	-1
517	2			1	CORT_0F04010	1	CPAR2_603040		-1	-1
518	2			1	CORT_0A10230	1	CPAR2_210880		-1	-1
519	2			1	CORT_0D04040	1	CPAR2_203930	Terpenoid cyclases/protein prenyltransferase alpha-alpha toroid; Prenyltransferase/squalene oxidase	-1	-1

520	2			1	CORT_0D02960	1	CPAR2_202880		-1	-1		
521	2			1	CORT_0A01680	1	CPAR2_801610		-1	-1		
522	2			1	CORT_0B02380	1	CPAR2_502200	Cyclin-dependent kinase, regulatory subunit	-1	-1		
523	2			1	CORT_0A11950	1	CPAR2_212660		-1	-1		
524	2			1	CORT_0F00877	1	CPAR2_100780		-1	-1		
525	2			1	CORT_0G02210	1	CPAR2_701980	Proteasome chaperone 3/4, fungi	-1	-1		
526	2			1	CORT_0A13370	1	CPAR2_214050		-1	-1		
527	2			1	CORT_0B11560	1	CPAR2_110460	Actin cortical patch SUR7/pH-response regulator PalI	-1	-1		
528	2			1	CORT_0H02370	1	CPAR2_600420		-1	-1		
529	2			1	CORT_0E03380	1	CPAR2_403360		-1	-1		
530	2			1	CORT_0B06820	1	CPAR2_105600	Ribosomal protein L36	-1	-1		
531	2			1	CORT_0G02550	1	CPAR2_702370	F-box domain, cyclin-like	-1	-1		
532	2			1	CORT_0E03040	1	CPAR2_403010	Mediator complex, subunit Med12	-1	-1		
533	2			1	CORT_0A09180	1	CPAR2_209920		-1	-1		
534	2			1	CORT_0A09470	1	CPAR2_210190		-1	-1		
535	2			1	CORT_0C07170	1	CPAR2_407830	DNA-directed RNA polymerase, M/15kDa subunit; DNA-directed RNA polymerase, subunit C11/M/9; Zinc finger, TFIIS-type	-1	-1		
536	2			1	CORT_0G02970	1	CPAR2_702860	C-CAP/cofactor C-like domain; Tubulin binding cofactor C	-1	-1		
537	2			1	CORT_0A07970	1	CPAR2_208690	WD40/YVTN repeat-like-containing domain; WD40 repeat; WD40-repeat-containing domain	-1	-1		
538	2			1	CORT_0F02140	1	CPAR2_601140		-1	-1		
539	2			1	CORT_0E01380	1	CPAR2_401330	Complex 1 LYR protein	-1	-1		
540	2			1	CORT_0F03450	1	CPAR2_602480		-1	-1		
541	2			1	CORT_0E06540	1	CPAR2_304340	Armadillo-type fold; Armadillo-like helical; Ataxin-10 domain	-1	-1		
542	2			1	CORT_0D04880	1	CPAR2_204770	UBA/THIF-type NAD/FAD binding fold; NAD(P)-binding domain; Molybdenum cofactor biosynthesis, MoeB; E1-like protein-activating enzyme G	-1	-1		
543	2			1	CORT_0B01990	1	CPAR2_501840	GSKIP/TIF31 domain; Protein of unknown function DUF727	-1	-1		
544	2			1	CORT_0E03890	1	CPAR2_403870		-1	-1		
545	2			1	CORT_0D05410	1	CPAR2_205290		-1	-1		
546	2			1	CORT_0E05780	1	CPAR2_303630		-1	-1		
547	2			1	CORT_0F04070	1	CPAR2_603100	Zinc finger C2H2-type/integrase DNA-binding domain	-1	-1		
548	2			1	CORT_0E05040	1	CPAR2_302900	Zn(2)-C6 fungal-type DNA-binding domain	-1	-1		
549	2			1	CORT_0A07495	1	CPAR2_208200		-1	-1		
550	2			1	CORT_0A03220	1	CPAR2_803245		-1	-1		
551	2			1	CORT_0H01050	1	CPAR2_301030		-1	-1		
552	2			1	CORT_0B10000	1	CPAR2_108900	GRASP55/65; GRASP55/65 PDZ-like domain	-1	-1		
553	2			1	CORT_0A09930	1	CPAR2_210600	Allergen V5/Tpx-1-related, conserved site; Allergen V5/Tpx-1-related; CAP domain	-1	-1		
554	2			1	CORT_0G01920	1	CPAR2_701770	H/ACA ribonucleoprotein complex, subunit Nop10	-1	-1		
555	2			1	CORT_0H00270	1	CPAR2_300210		-1	-1		
556	2			1	CORT_0A07730	1	CPAR2_208450		-1	-1		
557	2			1	CORT_0B09530	1	CPAR2_108440	Uncharacterised protein family SERF	-1	-1		
558	2			1	CORT_0G02520	1	CPAR2_702340	Vacuolar protein sorting 55	-1	-1		
559	2			1	CORT_0E01550	1	CPAR2_401500		-1	-1		
560	2			1	CORT_0D02630	1	CPAR2_202550	Ribosomal protein S30	-1	-1		
561	2			1	CORT_0B04310	1	CPAR2_103040		-1	-1		
562	2			1	CORT_0F00874	1	CPAR2_100770		-1	-1		
563	2			1	CORT_0A13130	1	CPAR2_213840	Cytochrome c oxidase copper chaperone; MTCP1	-1	-1		
564	2			1	CORT_0B06570	1	CPAR2_105360		-1	-1		
565	2			1	CORT_0C01440	1	CPAR2_807690		-1	-1		
566	2			1	CORT_0F00110	1	CPAR2_200010	Leucine-rich repeat; Leucine-rich repeat, typical subtype; Leucine rich repeat 4	-1	-1		
567	2			1	CORT_0B01560	1	CPAR2_501415	Protein of unknown function DUF3743	-1	-1		
568	2			1	CORT_0H00210	1	CPAR2_300150	Adenosine deaminase/editase	-1	-1		
569	2			1	CORT_0F02420	1	CPAR2_601420		-1	-1		
570	2			1	CORT_0G03650	1	CPAR2_703460.1		-1	-1		
571	2			1	CORT_0B07810	1	CPAR2_106560	Proteolipid membrane potential modulator	-1	-1		

572	2			1	CORT_0E01480	1	CPAR2_401430	Ribosomal protein, zinc-binding domain; Ribosomal protein L40e	-1	-1		
573	2			1	CORT_0D01375	1	CPAR2_201330	Micro-fibrillar-associated protein 1, C-terminal	-1	-1		
574	2			1	CORT_0B07540	1	CPAR2_106300	EKC/KEOPS complex, subunit Gon7	-1	-1		
575	2			1	CORT_0A07640	1	CPAR2_208350	Acyl-CoA N-acyltransferase; GNAT domain	-1	-1		
576	2			1	CORT_0B06040	1	CPAR2_104840		-1	-1		
577	2			1	CORT_0E06090	1	CPAR2_303910	Appr-1-p processing	-1	-1		
578	2			1	CORT_0F00550	1	CPAR2_100450	Peptidase S26A, signal peptidase I; Peptidase S26A, signal peptidase I, conserved site; Peptidase S24/S26A/S26B/S26C, beta-ribbon domain;	-1	-1		
579	2			1	CORT_0A06270	1	CPAR2_206910		-1	-1		
580	2			1	CORT_0C03230	1	CPAR2_805920		-1	-1		
581	2			1	CORT_0A10515	1	CPAR2_211170		-1	-1		
582	2			1	CORT_0D00940	1	CPAR2_200850	Prefoldin; Prefoldin beta-like	-1	-1		
583	2			1	CORT_0C03510	1	CPAR2_404080		-1	-1		
584	2			1	CORT_0A04360	1	CPAR2_804320		-1	-1		
585	2			1	CORT_0A08640	1	CPAR2_209370	RNA recognition motif domain; Nucleotide-binding, alpha-beta plait	-1	-1		
586	2			1	CORT_0A08810	1	CPAR2_209540	GRIP	-1	-1		
587	2			1	CORT_0A11570	1	CPAR2_212240	Nucleotide-binding, alpha-beta plait; RNA recognition motif domain	-1	-1		
588	2			1	CORT_0C04270	1	CPAR2_404850		-1	-1		
589	2			1	CORT_0B08100	1	CPAR2_106870	Zinc finger, DHHC-type, palmitoyltransferase	-1	-1		
590	2			1	CORT_0F00850	1	CPAR2_100730		-1	-1		
591	2			1	CORT_0E02080	1	CPAR2_402030		-1	-1		
592	2			1	CORT_0B02730	1	CPAR2_502530	Transcription initiation Spt4-like; Transcription initiation Spt4; Spt4/RpoE2 zinc finger	-1	-1		
593	2			1	CORT_0B05590	1	CPAR2_104350	PHF5-like	-1	-1		
594	2			1	CORT_0G04240	1	CPAR2_704060	HSP20-like chaperone; Alpha crystallin/Hsp20 domain	-1	-1		
595	2			1	CORT_0D00260	1	CPAR2_200180	Zinc finger, RING-type; Zinc finger, RING/FYVE/PHD-type; Anaphase-promoting complex subunit 11	-1	-1		
596	2			1	CORT_0C01530	1	CPAR2_807610	FAR-17a/AIG1-like protein	-1	-1		
597	2			1	CORT_0E05600	1	CPAR2_303460	Non-classical export protein 1	-1	-1		
598	2			1	CORT_0C05105	1	CPAR2_405695		-1	-1		
599	2			1	CORT_0A09130	1	CPAR2_209870	Cytochrome c oxidase, subunit VIIa, fungal	-1	-1		
600	2			1	CORT_0C02810	1	CPAR2_806330	Oligosaccaryltransferase	-1	-1		
601	2			1	CORT_0A00180	1	CPAR2_800070		-1	-1		
602	2			1	CORT_0E00880	1	CPAR2_400840	Transmembrane protein 93	-1	-1		
603	2			1	CORT_0B05420	1	CPAR2_104195		-1	-1		
604	2			1	CORT_0B06790	1	CPAR2_105570	RNA recognition motif domain; Nucleotide-binding, alpha-beta plait	-1	-1		
605	2			1	CORT_0B04710	1	CPAR2_103500	Ribonuclease P/MRP protein subunit	-1	-1		
606	2			1	CORT_0A04090	1	CPAR2_804060	Acetyl-coenzyme A carboxyltransferase, N-terminal; Single hybrid motif; Biotin carboxylation domain; Acetyl-CoA carboxylase, central domain;	-1	-1		
607	2			1	CORT_0A04420	1	CPAR2_804260	Ornithine cyclodeaminase/mu-crystallin; NAD(P)-binding domain	-1	-1		
608	2			1	CORT_0B01630	1	CPAR2_501480	Histone deacetylase domain; Histone deacetylase superfamily	-1	-1		
609	2			1	CORT_0C06265	1	CPAR2_406905		-1	-1		
610	2			1	CORT_0F00100	1	CPAR2_100020	Leucine-rich repeat; Leucine rich repeat 4	-1	-1		
611	2			1	CORT_0D02110	1	CPAR2_202060		-1	-1		
612	2			1	CORT_0E01770	1	CPAR2_401720		-1	-1		
613	2			1	CORT_0A00170	1	CPAR2_800060		-1	-1		
614	2			1	CORT_0B03110	1	CPAR2_101820	Glutaredoxin-like; Thioredoxin-like fold	-1	-1		
615	2			1	CORT_0G02840	1	CPAR2_702710		-1	-1		
616	2			1	CORT_0A04850	1	CPAR2_804850		-1	-1		
617	2			1	CORT_0F00670	1	CPAR2_100565		-1	-1		
618	2			1	CORT_0B06660	1	CPAR2_105440		-1	-1		
619	2			1	CORT_0B03960	1	CPAR2_801735		-1	-1		
620	2			1	CORT_0A03020	1	CPAR2_802970	GPI-GlcNAc transferase complex, PIG-H component, conserved domain	-1	-1		
621	2			1	CORT_0C02650	1	CPAR2_806500	Zinc finger C2H2-type/integrase DNA-binding domain; Zinc finger, C2H2-like; Zinc finger, C2H2	-1	-1		
622	2			1	CORT_0A11930	1	CPAR2_212610	ATPase, V0 complex, subunit E	-1	-1		
623	2			1	CORT_0D06010	1	CPAR2_205880	Ribosomal protein S14, conserved site; Ribosomal protein S14	-1	-1		

624	2			1	CORT_0B04300	1	CPAR2_103030	Ribosomal protein L30, ferredoxin-like fold domain; Ribosomal protein L30, conserved site; Ribosomal protein L30, bacterial-type	-1	-1		
625	2			1	CORT_0C06930	1	CPAR2_407590		-1	-1		
626	2			1	CORT_0G03420	1	CPAR2_703250		-1	-1		
627	2			1	CORT_0D02620	1	CPAR2_202540		-1	-1		
628	2			1	CORT_0B00830	1	CPAR2_500680.1		-1	-1		
629	2			1	CORT_0D03740	1	CPAR2_203630	Fat storage-inducing transmembrane protein	-1	-1		
630	2			1	CORT_0E04120	1	CPAR2_302000	Leucine-rich repeat; Leucine rich repeat 4	-1	-1		
631	2			1	CORT_0B07240	1	CPAR2_106015		-1	-1		
632	2			1	CORT_0E04300	1	CPAR2_302200		-1	-1		
633	2			1	CORT_0F01260	1	CPAR2_101170		-1	-1		
634	2			1	CORT_0F03580	1	CPAR2_602610	Uncharacterised domain Flo11-reatedl, N-terminal	-1	-1		
635	2			1	CORT_0F04605	1	CPAR2_603640		-1	-1		
636	2			1	CORT_0D04140	1	CPAR2_204040		-1	-1		
637	2			1	CORT_0G03230	1	CPAR2_703090	Tetratricopeptide repeat; Tetratricopeptide-like helical; Tetratricopeptide repeat-containing domain; Tetratricopeptide TPR2	-1	-1		
638	2			1	CORT_0D02600	1	CPAR2_202520		-1	-1		
639	2			1	CORT_0E06360	1	CPAR2_304170	Ribosomal protein L34	-1	-1		
640	2			1	CORT_0C03700	1	CPAR2_404350		-1	-1		
641	2			1	CORT_0F03360	1	CPAR2_602380		-1	-1		
642	13	13	g1708.t1 g1777.t1 g2963.t1 g3733.t2 g3751.t1 g3768.t1 g4126.t1 g4133.t1 g4138.t1 g4816.t2 g4817.t1 g4837.t1 g909.t1						13	13		
643	10	10	g1774.t1 g29.t1 g2935.t1 g2946.t1 g2994.t1 g326.t1 g333.t1 g3776.t1 g3791.t1 g4847.t1						10	10		
644	10	10	g1072.t1 g2149.t1 g2155.t1 g3132.t1 g323.t1 g4041.t1 g4					Leucine-rich repeat; Leucine rich repeat 4	10	10		
645	8	8	g1773.t1 g3713.t1 g5397.t1 g5701.t1 g5702.t1 g61.t1 g85					Protein of unknown function DUF3445	8	8		
646	7	7	g1717.t1 g1737.t1 g2932.t1 g2948.t1 g2966.t1 g42.t1 g56.t1						7	7		
647	6	6	g1538.t1 g2148.t1 g2156.t1 g3135.t3 g38.t1 g65.t1					Leucine-rich repeat; Leucine rich repeat 4	6	6		
648	6	6	g2224.t1 g278.t1 g308.t1 g4075.t1 g5622.t1 g5874.t1					DDE superfamily endonuclease, CENP-B-like; HTH CenpB-type DNA-binding domain	6	6		
649	6	6	g167.t1 g2941.t1 g2971.t1 g32.t1 g4848.t1 g58.t1						6	6		
650	5	5	g2310.t1 g3059.t1 g46.t1 g5275.t1 g856.t1						5	5		
651	4	4	g3822.t1 g3823.t1 g3824.t1 g3825.t1					Flocculin type 3 repeat; Cytadhesin P30P32	4	4		
652	4	4	g1291.t1 g3758.t1 g4854.t1 g883.t						4	4		
653	4	4	g1811.t1 g1821.t1 g1828.t1 g1829.t1						4	4		
654	3	3	g4914.t1 g66.t1 g67.t1					Retrotransposon gag protein	3	3		
655	3	3	g2147.t1 g3136.t1 g321.t1					Peptidase S10, serine carboxypeptidase, active site; Peptidase S10, serine carboxypeptidase	3	3		
656	3	3	g4287.t1 g4288.t1 g4292.t1						3	3		
657	3	3	g1753.t1 g1790.t1 g4881.t1					PT repeat	3	3		
658	3	3	g26.t1 g3112.t1 g3114.t1					CDR ABC transporter	3	3		
659	3	3	g2200.t1 g2201.t1 g4004.t1						3	3		
660	3	3	g4068.t1 g4323.t2 g637.t1						3	3		
661	3	3	g3036.t1 g3054.t1 g4.t1					Peptidase A1; Peptidase aspartic; Peptidase aspartic, catalytic	3	3		
662	3	3	g1726.t1 g1767.t1 g1778.t1						3	3		
663	3	3	g1295.t1 g3703.t1 g3796.t1						3	3		
664	2	2	g4164.t1 g4169.t1					Adhesion domain, bacterial; Agglutinin-like protein, N-terminal	2	2		
665	2	2	g446.t1 g450.t1						2	2		
666	2	2	g671.t1 g679.t1						2	2		
667	2	2	g1311.t1 g4872.t1						2	2		
668	2	2	g1278.t1 g350.t						2	2		
669	2	2	g1.t1 g4866.t1						2	2		
670	2	2	g4100.t1 g4477.t1						2	2		
671	2	2	g5015.t1 g5022.t1						2	2		
672	2	2	g2076.t1 g4481.t1						2	2		
673	2	2	g1638.t1 g783.t					F-box domain, cyclin-like	2	2		
674	2	2	g903.t1 g922.t1						2	2		
675	2	2	g1614.t1 g5916.t1						2	2		

676	2	2	g361.t1 g63.t2		2	2
677	2	2	g1706.t1 g1707.t1	Peptidase A1; Peptidase aspartic; Peptidase aspartic, catalytic	2	2
678	2	2	g1583.t1 g1584.t1	Leucine-rich repeat; Leucine rich repeat 4	2	2
679	2	2	g3056.t1 g9.t1		2	2
680	2	2	g2918.t1 g3073.t1		2	2
681	2	2	g2.t1 g3052.t1	Nucleotide-binding, alpha-beta plait	2	2
682	2	2	g2077.t1 g4482.t1		2	2
683	2	2	g2764.t1 g3883.t1		2	2
684	2	2	g2199.t1 g4005.t1	F-box domain, cyclin-like	2	2
685	2	2	g1639.t1 g1650.t1		2	2
686	2	2	g1073.t1 g1075.t1		2	2
687	1	1	g4346.t1		1	1
688	1	1	g5215.t1		1	1
689	1	1	g520.t1		1	1
690	1	1	g347.t1		1	1
691	1	1	g5353.t1		1	1
692	1	1	g4104.t1		1	1
693	1	1	g4753.t1		1	1
694	1	1	g1126.t1		1	1
695	1	1	g377.t1	DDE superfamily endonuclease, CENP-B-like	1	1
696	1	1	g4407.t1	Leucine-rich repeat; Leucine rich repeat 4	1	1
697	1	1	g4063.t2	Leucine-rich repeat	1	1
698	1	1	g5951.t1		1	1
699	1	1	g3719.t1		1	1
700	1	1	g76.t1		1	1
701	1	1	g21.t1	Capsid/spike protein, ssDNA virus; Major spike protein G; Microvirus H protein (pilot protein); Capsid protein F	1	1
702	1	1	g1230.t1		1	1
703	1	1	g4040.t1		1	1
704	1	1	g2398.t1		1	1
705	1	1	g590.t1		1	1
706	1	1	g2451.t1	F-box domain, cyclin-like	1	1
707	1	1	g1491.t1		1	1
708	1	1	g2761.t1	Small ubiquitin-related modifier, SUMO	1	1
709	1	1	g2452.t1		1	1
710	1	1	g2809.t1	Kinetochore subunit NKP2	1	1
711	1	1	g3412.t1		1	1
712	1	1	g322.t1		1	1
713	1	1	g68.t1	Reverse transcriptase	1	1
714	1	1	g4853.t1		1	1
715	1	1	g383.t1		1	1
716	1	1	g429.t1		1	1
717	1	1	g4147.t1		1	1
718	1	1	g789.t1	Zn(2)-C6 fungal-type DNA-binding domain	1	1
719	1	1	g5211.t1		1	1
720	1	1	g5021.t1	Ribonuclease H-like domain	1	1
721	1	1	g1477.t1		1	1
722	1	1	g4037.t1		1	1
723	1	1	g5608.t1	PINIT domain; DNA-binding SAP; Zinc finger, MIZ-type	1	1
724	1	1	g4941.t1		1	1
725	1	1	g5527.t1	Spo11/DNA topoisomerase VI, subunit A	1	1
726	1	1	g4368.t1		1	1
727	1	1	g684.t1		1	1

728	1	1	g2174.t1						1	1		
729	1	1	g628.t1						1	1		
730	1	1	g4578.t1						1	1		
731	1	1	g1837.t1						1	1		
732	1	1	g1074.t1						1	1		
733	1	1	g4128.t1						1	1		
734	1	1	g5348.t1						1	1		
735	1	1	g835.t1						1	1		
736	1	1	g1615.t1						1	1		
737	1	1	g5798.t1						1	1		
738	1	1	g4277.t1						1	1		
739	1	1	g1070.t1					Leucine-rich repeat, typical subtype	1	1		
740	1	1	g521.t1						1	1		
741	1	1	g1157.t1					Mating-type protein MAT alpha 1; Alpha box	1	1		
742	1	1	g3552.t1						1	1		
743	1	1	g3720.t1						1	1		
744	1	1	g836.t1					Winged helix-turn-helix transcription repressor DNA-binding; Spo11/DNA topoisomerase VI, subunit A, N-terminal; Spo11/DNA topoisomerase	1	1		
745	1	1	g4822.t1						1	1		
746	1	1	g3540.t1						1	1		
747	1	1	g5274.t1						1	1		
748	1	1	g958.t1					Domain of unknown function DUF2399; Spo11/DNA topoisomerase VI, subunit A	1	1		
749	1	1	g1343.t1						1	1		
750	1	1	g4940.t1						1	1		
751	1	1	g3256.t1						1	1		
752	1	1	g4055.t1					Leucine-rich repeat; Leucine-rich repeat, typical subtype; Leucine rich repeat 4	1	1		
753	1	1	g872.t1						1	1		
754	1	1	g25.t1						1	1		
755	1	1	g7.t1					Leucine-rich repeat; Leucine-rich repeat, typical subtype; Leucine rich repeat 4	1	1		
756	1	1	g5624.t1						1	1		
757	1	1	g5106.t1						1	1		
758	1	1	g1564.t1						1	1		
759	1	1	g4136.t1						1	1		
760	1	1	g1387.t1						1	1		
761	1	1	g4140.t1						1	1		
762	1	1	g1656.t1						1	1		
763	1	1	g172.t1						1	1		
764	1	1	g4638.t1						1	1		
765	1	1	g1663.t1						1	1		
766	1	1	g3293.t1						1	1		
767	1	1	g470.t1					Uncharacterised domain Flo11-reatedl, N-terminal	1	1		
768	1	1	g2357.t1						1	1		
769	1	1	g1204.t1						1	1		
770	1	1	g797.t1						1	1		
771	1	1	g834.t1						1	1		
772	1	1	g2598.t1					RNA polymerase II, large subunit, CTD; ENTH/VHS	1	1		
773	1	1	g1231.t1						1	1		
774	1	1	g1585.t1						1	1		
775	1	1	g2169.t1						1	1		
776	1	1	g1476.t1						1	1		
777	1	1	g5231.t1						1	1		
778	1	1	g547.t1					Heat shock protein DnaJ, N-terminal; Heat shock protein DnaJ; Heat shock protein DnaJ, conserved site	1	1		
779	1	1	g3767.t1						1	1		

780	1	1	g2104.t1						1	1		
781	1	1	g1704.t1						1	1		
782	1	1	g519.t1						1	1		
783	1	1	g1195.t1						1	1		
784	1	1	g1287.t1						1	1		
785	1	1	g5060.t1						1	1		
786	1	1	g3133.t1						1	1		
787	1	1	g2649.t1					RNA recognition motif domain; Nucleotide-binding, alpha-beta plait	1	1		
788	1	1	g5019.t1						1	1		
789	1	1	g540.t1					Hyphally-regulated cell wall protein, N-terminal	1	1		
790	1	1	g1629.t1						1	1		
791	1	1	g3134.t1						1	1		
792	1	1	g4896.t1						1	1		
793	1	1	g1633.t1						1	1		
794	1	1	g4178.t1						1	1		
795	1	1	g2681.t1					HTH CenpB-type DNA-binding domain	1	1		
796	1	1	g3101.t1						1	1		
797	1	1	g1712.t1						1	1		
798	1	1	g5731.t1						1	1		
799	1	1	g1601.t1						1	1		
800	1	1	g1581.t1						1	1		
801	1	1	g3631.t1						1	1		
802	1	1	g4930.t1						1	1		
803	1	1	g1546.t1						1	1		
804	1	1	g3551.t1						1	1		
805	1	1	g5020.t1						1	1		
806	1	1	g22.t1					C-protein; Replication gene A protein	1	1		
807	1	1	g3632.t1						1	1		
808	1	1	g3701.t1						1	1		
809	1	1	g1334.t1						1	1		
810	1	1	g4184.t1						1	1		
811	1	1	g5819.t1					Kinetochore Sim4 complex subunit Fta4	1	1		
812	1	1	g4556.t1						1	1		
813	1	1	g5913.t1						1	1		
814	1	1	g2559.t1						1	1		
815	1	1	g5154.t1						1	1		
816	1	1	g3923.t1						1	1		
817	1	1	g445.t1						1	1		
818	1	1	g5658.t1						1	1		
819	1	1	g884.t1						1	1		
820	1	1	g1263.t1						1	1		
821	1	1	g2237.t1					Amidohydrolase 2	1	1		
822	1	1	g5213.t1						1	1		
823	1	1	g449.t1						1	1		
824	1	1	g1183.t1						1	1		
825	1	1	g5646.t1						1	1		
826	1	1	g3832.t1						1	1		
827	1	1	g5577.t1						1	1		
828	1	1	g3952.t1						1	1		
829	1	1	g64.t1					Reverse transcriptase; Integrase, catalytic core; Ribonuclease H-like domain; Retroviral aspartyl protease	1	1		
830	1	1	g5208.t1						1	1		
831	1	1	g4074.t1						1	1		

832	1	1	g5914.t1						1	1		
833	1	1	g5210.t1					Spo11/DNA topoisomerase VI, subunit A	1	1		
834	1	1	g4043.t1					Leucine-rich repeat; Leucine rich repeat 4	1	1		
835	1	1	g1628.t1						1	1		
836	1	1	g808.t1						1	1		
837	1	1	g2308.t1					Leucine-rich repeat	1	1		
838	1	1	g4632.t1						1	1		
839	1	1	g4347.t1						1	1		
840	1	1	g4354.t1					Zinc finger C2H2-type/integrase DNA-binding domain	1	1		
841	1	1	g3259.t1					WD40/YVTN repeat-like-containing domain; WD40-repeat-containing domain; WD40 repeat; WD40 repeat, conserved site	1	1		
842	1	1	g5225.t1						1	1		
843	1	1	g5212.t1						1	1		
844	1	1	g782.t1						1	1		
845	1	1	g4087.t1						1	1		
846	1	1	g2939.t1						1	1		
847	1	1	g4054.t1					Leucine-rich repeat; Leucine-rich repeat, typical subtype; Leucine rich repeat 4	1	1		
848	1	1	g4767.t1					DDE superfamily endonuclease, CENP-B-like	1	1		
849	1	1	g929.t1						1	1		
850	1	1	g3735.t1						1	1		
851	1	1	g331.t1						1	1		
852	1	1	g5531.t1						1	1		
853	1	1	g5216.t1						1	1		
854	1	1	g472.t1						1	1		
855	1	1	g4806.t1						1	1		
856	1	1	g2748.t1						1	1		
857	1	1	g5303.t1					G-patch domain	1	1		
858	1	1	g200.t1						1	1		
859	1	1	g1816.t1						1	1		
860	1	1	g5973.t1						1	1		
861	1	1	g2514.t1						1	1		
862	1	1	g2706.t1					Cytochrome P450, conserved site	1	1		
863	1	1	g3652.t1						1	1		
864	1	1	g5209.t1						1	1		
865	1	1	g2491.t1						1	1		
866	1	1	g309.t1						1	1		
867	1	1	g5915.t1						1	1		
868	2			2	CORT_0A05480 CORT_0G02660				0	-2		
869	1			1	CORT_0E05750			Homeodomain-like; Homeodomain; Homeobox, conserved site	0	-1		
870	1			1	CORT_0G02280				0	-1		
871	1			1	CORT_0D06020				0	-1		
872	1			1	CORT_0D05210				0	-1		
873	1			1	CORT_0B03640				0	-1		
874	1			1	CORT_0C06760				0	-1		
875	1			1	CORT_0B00380			Leucine-rich repeat	0	-1		
876	1			1	CORT_0A05190				0	-1		
877	1			1	CORT_0D04410				0	-1		
878	1			1	CORT_0C00520				0	-1		
879	1			1	CORT_0B02920				0	-1		
880	1			1	CORT_0G03610				0	-1		
881	1			1	CORT_0C03240				0	-1		
882	1			1	CORT_0E02650				0	-1		
883	1			1	CORT_0A07620				0	-1		

884	1			1	CORT_0A00590				0	-1		
885	1			1	CORT_0A00260				0	-1		
886	1			1	CORT_0A06530				0	-1		
887	1			1	CORT_0E02060				0	-1		
888	1			1	CORT_0G01280				0	-1		
889	1			1	CORT_0A03610				0	-1		
890	1			1	CORT_0B00100				0	-1		
891	1			1	CORT_0B01680				0	-1		
892	1			1	CORT_0C02730				0	-1		
893	1			1	CORT_0A01815				0	-1		
894	1			1	CORT_0B05350				0	-1		
895	1			1	CORT_0C02520			Flocculin type 3 repeat	0	-1		
896	1			1	CORT_0E04960				0	-1		
897	1			1	CORT_0B09980				0	-1		
898	1			1	CORT_0F01640				0	-1		
899	1			1	CORT_0H02250			F-box domain, cyclin-like	0	-1		
900	1			1	CORT_0D07670			Leucine-rich repeat; Leucine rich repeat 4	0	-1		
901	1			1	CORT_0G00710				0	-1		
902	1			1	CORT_0B03430				0	-1		
903	1			1	CORT_0B03310				0	-1		
904	1			1	CORT_0F01610			Histone H5	0	-1		
905	1			1	CORT_0H02500				0	-1		
906	1			1	CORT_0F01920				0	-1		
907	1			1	CORT_0A09810			CsbD-like	0	-1		
908	1			1	CORT_0E00600				0	-1		
909	1			1	CORT_0A09590				0	-1		
910	1			1	CORT_0G03240				0	-1		
911	1			1	CORT_0B08430				0	-1		
912	1			1	CORT_0E04130				0	-1		
913	1			1	CORT_0E03960				0	-1		
914	1			1	CORT_0D04940			Cyclin PHO80-like	0	-1		
915	1			1	CORT_0C01560			Kinetochore Sim4 complex subunit Fta4	0	-1		
916	1			1	CORT_0G04490				0	-1		
917	1			1	CORT_0A04770				0	-1		
918	1			1	CORT_0B05520				0	-1		
919	1			1	CORT_0E00990				0	-1		
920	1			1	CORT_0D06240				0	-1		
921	1			1	CORT_0B02710				0	-1		
922	1			1	CORT_0A04610				0	-1		
923	1			1	CORT_0G04160				0	-1		
924	1			1	CORT_0E05330				0	-1		
925	1			1	CORT_0A11960				0	-1		
926	1			1	CORT_0E02550				0	-1		
927	1			1	CORT_0E03800				0	-1		
928	1			1	CORT_0A01530			Leucine-rich repeat	0	-1		
929	1			1	CORT_0A08100				0	-1		
930	1			1	CORT_0E04810			Peptidase aspartic, catalytic; Peptidase aspartic; Peptidase A1	0	-1		
931	1			1	CORT_0G00560				0	-1		
932	1			1	CORT_0F01980			Bul1, N-terminal; Bul1, C-terminal	0	-1		
933	1			1	CORT_0F00120			Leucine-rich repeat; Leucine rich repeat 4	0	-1		
934	1			1	CORT_0G03250				0	-1		
935	1			1	CORT_0B09290				0	-1		

936	1			1	CORT_0E00450				0	-1		
937	1			1	CORT_0B01480				0	-1		
938	1			1	CORT_0A09480				0	-1		
939	1			1	CORT_0D05000				0	-1		
940	1			1	CORT_0G01580				0	-1		
941	1			1	CORT_0E02540				0	-1		
942	1			1	CORT_0B07780				0	-1		
943	1			1	CORT_0G02670				0	-1		
944	1			1	CORT_0G04500				0	-1		
945	1			1	CORT_0B10915				0	-1		
946	1			1	CORT_0E04255				0	-1		
947	1			1	CORT_0G03550				0	-1		
948	1			1	CORT_0A09490				0	-1		
949	1			1	CORT_0C00110				0	-1		
950	1			1	CORT_0E02050				0	-1		
951	1			1	CORT_0B10410			PINIT domain; Zinc finger, MIZ-type	0	-1		
952	1			1	CORT_0E04970				0	-1		
953	1			1	CORT_0G01550				0	-1		
954	1			1	CORT_0H02260				0	-1		
955	1			1	CORT_0C01130				0	-1		
956	1			1	CORT_0G02180				0	-1		
957	1			1	CORT_0G02850				0	-1		
958	1			1	CORT_0A03280				0	-1		
959	1			1	CORT_0E00250				0	-1		
960	1			1	CORT_0C03590				0	-1		
961	1			1	CORT_0E06020				0	-1		
962	1			1	CORT_0A08340				0	-1		
963	1			1	CORT_0A09150			RNA polymerase II, large subunit, CTD; ENTH/VHS	0	-1		
964	1			1	CORT_0D01700				0	-1		
965	1			1	CORT_0G04060			Leucine-rich repeat; Leucine rich repeat 4	0	-1		
966	1			1	CORT_0A12370				0	-1		
967	1			1	CORT_0B02810				0	-1		
968	1			1	CORT_0E01870				0	-1		
969	1			1	CORT_0B02850				0	-1		
970	1			1	CORT_0H02030				0	-1		
971	1			1	CORT_0G03640				0	-1		
972	1			1	CORT_0B09880				0	-1		
973	1			1	CORT_0B08880			SMAD/FHA domain; Forkhead-associated (FHA) domain	0	-1		
974	1			1	CORT_0H02530				0	-1		
975	4					4	CPAR2_101640 CPAR2_301590 CPAR2_502600 CPAR2_600970		-4	0		
976	2					2	CPAR2_101780 CPAR2_600920		-2	0		
977	2					2	CPAR2_502970 CPAR2_702490		-2	0		
978	2					2	CPAR2_108120 CPAR2_109110		-2	0		
979	2					2	CPAR2_100840	Nucleotide-binding, alpha-beta plait	-2	0		
980	2					2	CPAR2_808310 CPAR2_808320		-2	0		
981	2					2	CapafMp06.1 Ca	Reverse transcriptase; HNH endonuclease; Group II intron, maturase-specific; HNH nuclease	-2	0		
982	2					2	CPAR2_200530 CPAR2_806630		-2	0		
983	2					2	CPAR2_400260 CPAR2_400370		-2	0		
984	2					2	CPAR2_101890 CPAR2_302130		-2	0		
985	2					2	CPAR2_502550 CPAR2_502640		-2	0		
986	1					1	CPAR2_803190		-1	0		
987	1					1	CPAR2_503010		-1	0		

988	1					1	CPAR2_703370		-1	0		
989	1					1	CPAR2_701380		-1	0		
990	1					1	CPAR2_600990		-1	0		
991	1					1	CPAR2_109320	PINIT domain; Zinc finger, MIZ-type	-1	0		
992	1					1	CPAR2_100950		-1	0		
993	1					1	CPAR2_503280		-1	0		
994	1					1	CPAR2_301870		-1	0		
995	1					1	CPAR2_802450		-1	0		
996	1					1	CPAR2_103320		-1	0		
997	1					1	CPAR2_402020		-1	0		
998	1					1	CapafMp03.2	LAGLIDADG DNA endonuclease	-1	0		
999	1					1	CPAR2_302810		-1	0		
1000	1					1	CPAR2_104135		-1	0		
1001	1					1	CPAR2_212640		-1	0		
1002	1					1	CPAR2_600290		-1	0		
1003	1					1	CPAR2_213890		-1	0		
1004	1					1	CPAR2_402590		-1	0		
1005	1					1	CPAR2_600940	Bul1, N-terminal; Bul1, C-terminal	-1	0		
1006	1					1	CapafMp05	Copper centre Cu(A); Cytochrome c oxidase, subunit II; Cytochrome C oxidase subunit II-like, transmembrane domain; Cytochrome C oxidase	-1	0		
1007	1					1	CapafMp17	ATPase, F0 complex, subunit A; ATPase, F0 complex, subunit A, active site	-1	0		
1008	1					1	CapafMp09	NADH:ubiquinone oxidoreductase, chain 4, N-terminal; NADH:ubiquinone/plastoquinone oxidoreductase; NADH-quinone oxidoreductase, chain	-1	0		
1009	1					1	CPAR2_502730		-1	0		
1010	1					1	CPAR2_808630		-1	0		
1011	1					1	CPAR2_602030		-1	0		
1012	1					1	CPAR2_402490		-1	0		
1013	1					1	CPAR2_805180		-1	0		
1014	1					1	CPAR2_800140		-1	0		
1015	1					1	CapafMp03.1	Intron endonuclease, group I; UvrABC system subunit C, N-terminal	-1	0		
1016	1					1	CPAR2_213060		-1	0		
1017	1					1	CPAR2_303600		-1	0		
1018	1					1	CPAR2_301890		-1	0		
1019	1					1	CPAR2_503640		-1	0		
1020	1					1	CPAR2_207870		-1	0		
1021	1					1	CPAR2_702570		-1	0		
1022	1					1	CPAR2_702470		-1	0		
1023	1					1	CPAR2_807580	Kinetochore Sim4 complex subunit Fta4	-1	0		
1024	1					1	CPAR2_703890		-1	0		
1025	1					1	CPAR2_212630		-1	0		
1026	1					1	CPAR2_102340		-1	0		
1027	1					1	CPAR2_212990		-1	0		
1028	1					1	CPAR2_102380	DDE superfamily endonuclease, CENP-B-like	-1	0		
1029	1					1	CPAR2_800500		-1	0		
1030	1					1	CapafMp03	Di-haem cytochrome, transmembrane; Cytochrome b/b6, C-terminal; Cytochrome b/b6, N-terminal; Cytochrome b/b6	-1	0		
1031	1					1	CPAR2_302720		-1	0		
1032	1					1	CPAR2_702130		-1	0		
1033	1					1	CPAR2_200020	Leucine-rich repeat; Leucine-rich repeat, typical subtype	-1	0		
1034	1					1	CapafMp02	NADH:ubiquinone/plastoquinone oxidoreductase	-1	0		
1035	1					1	CapafMp13	Cytochrome c/ubiquinol oxidase subunit III; Cytochrome c oxidase, subunit III; Cytochrome c oxidase, subunit III, 4-helical bundle	-1	0		
1036	1					1	CPAR2_204130.1		-1	0		
1037	1					1	CPAR2_400950		-1	0		
1038	1					1	CPAR2_209890	RNA polymerase II, large subunit, CTD; ENTH/VHS	-1	0		
1039	1					1	CPAR2_205090		-1	0		

1040	1					1	CPAR2_603650		-1	0		
1041	1					1	CPAR2_302820		-1	0		
1042	1					1	CapafMp01	NADH:ubiquinone/plastoquinone oxidoreductase, chain 3	-1	0		
1043	1					1	CPAR2_600010	Leucine-rich repeat; Leucine rich repeat 4	-1	0		
1044	1					1	CPAR2_600910		-1	0		
1045	1					1	CPAR2_101540		-1	0		
1046	1					1	CPAR2_211330		-1	0		
1047	1					1	CPAR2_803840		-1	0		
1048	1					1	CPAR2_106530		-1	0		
1049	1					1	CPAR2_503270		-1	0		
1050	1					1	CPAR2_704280		-1	0		
1051	1					1	CPAR2_700440		-1	0		
1052	1					1	CPAR2_808910		-1	0		
1053	1					1	CPAR2_102190	Nucleolar protein Dnt1-like, N-terminal	-1	0		
1054	1					1	CPAR2_208830		-1	0		
1055	1					1	CPAR2_203960	DDE superfamily endonuclease, CENP-B-like	-1	0		
1056	1					1	CPAR2_207660.	Kinetochore subunit NKP2	-1	0		
1057	1					1	CPAR2_600860		-1	0		
1058	1					1	CPAR2_701840		-1	0		
1059	1					1	CPAR2_213370		-1	0		
1060	1					1	CPAR2_208410		-1	0		
1061	1					1	CPAR2_206070	Chromosome segregation protein Spc25	-1	0		
1062	1					1	CPAR2_103110		-1	0		
1063	1					1	CPAR2_805490		-1	0		
1064	1					1	CPAR2_102120		-1	0		
1065	1					1	CPAR2_808850		-1	0		
1066	1					1	CPAR2_501370		-1	0		
1067	1					1	CPAR2_101650		-1	0		
1068	1					1	CPAR2_206100		-1	0		
1069	1					1	CPAR2_109330		-1	0		
1070	1					1	CapafMp16	ATPase, F0 complex, subunit 8, mitochondrial, fungal	-1	0		
1071	1					1	CPAR2_303190		-1	0		
1072	1					1	CPAR2_804470	Proline racemase	-1	0		
1073	1					1	CPAR2_208330		-1	0		
1074	1					1	CPAR2_208820		-1	0		
1075	1					1	CapafMp11	NADH:ubiquinone/plastoquinone oxidoreductase, chain 6	-1	0		
1076	1					1	CPAR2_102010		-1	0		
1077	1					1	CapafMp14	NADH-ubiquinone oxidoreductase chain 4L/K	-1	0		
1078	1					1	CPAR2_701410		-1	0		
1079	1					1	CPAR2_702050		-1	0		
1080	1					1	CPAR2_500010		-1	0		
1081	1					1	CPAR2_503060		-1	0		
1082	1					1	CPAR2_205875		-1	0		
1083	1					1	CPAR2_703380	Major coat protein, L-A virus	-1	0		
1084	1					1	CPAR2_212650		-1	0		
1085	1					1	CPAR2_204890		-1	0		
1086	1					1	CPAR2_703440		-1	0		
1087	1					1	CPAR2_102170		-1	0		
1088	1					1	CapafMp06.4	Homing endonuclease, LAGLIDADG/HNH	-1	0		
1089	1					1	CPAR2_207790		-1	0		
1090	1					1	CPAR2_503700		-1	0		
1091	1					1	CPAR2_102020		-1	0		

1092	1					1	CPAR2_206540		-1	0		
1093	1					1	CapafMp06.3	Homing endonuclease, LAGLIDADG/HNH	-1	0		
1094	1					1	CPAR2_212340	CENP-A-nucleosome distal centromere subunit CENP-Q	-1	0		
1095	1					1	CPAR2_501530		-1	0		
1096	1					1	CPAR2_703900		-1	0		
1097	1					1	CPAR2_104360		-1	0		
1098	1					1	CPAR2_405110		-1	0		
