# Supplementary material for: The Genomic Aftermath of Hybridization in the Opportunistic Pathogen Candida metapsilosis
Source: PLoS Genet. 2015 Oct 30;11(10):e1005626. doi: 10.1371/journal.pgen.1005626 (PMC4627764; doi:10.1371/journal.pgen.1005626)
Supplement: S6 Table — For each species, the table lists: NCBI Taxonomy species identifier, species name, PhylomeDB proteome code, genome source and date when the genome was downloaded. (PDF) [file pgen.1005626.s020.pdf]

| Taxon Id | Species Name | PhylomeDB proteome code | Source | Date |
|---|---|---|---|---|
| 4922 | Pichia pastoris | PICPA.1 | Ghent university | 10/06/2009 |
| 4924 | Pichia stipitis | PICST.2 | integr8 | 12/09/2010 |
| 4929 | Pichia guilliermondii | PICGU.1 | Broad_Institute | 01/04/2007 |
| 4932 | Saccharomyces cerevisiae | YEAST.8 | YGOB | 28/10/2013 |
| 4952 | Yarrowia lipolytica | YARLI.2 | integr8 | 12/09/2010 |
| 4959 | Debaryomyces hansenii | DEBHA.3 | Broad_Institute | 11/11/2010 |
| 5476 | Candida albicans | CANAL.3 | Broad_Institute | 11/11/2010 |
| 5478 | Candida glabrata | CANGA.4 | Genolevures | 02/04/2011 |
| 5480 | Candida parapsilosis | CANPA.1 | Broad Institute | 18/12/2008 |
| 5482 | Candida tropicalis | CANTR.1 | Broad_Institute | 01/04/2007 |
| 36911 | Clavispora lusitaniae | CLALS.1 | Broad_Institute | 01/04/2007 |
| 36914 | Lodderomyces elongisporus | LODEL.2 | Broad_Institute | 11/11/2010 |
| 42374 | Candida dubliniensis | CANDU.2 | Sanger Institute | 07/04/2010 |
| 45596 | Candida tenuis | CANTE.1 | JGI | 03/04/2013 |
| 46583 | Candida tanzawaensis | 46583.1 | JGI | 03/04/2013 |
| 51930 | Candida caseinolytica | 51930.1 | JGI | 03/04/2013 |
| 130810 | Candida arabinofermentans | 130810.1 | JGI | 03/04/2013 |
| 273131 | Candida bracarensis | 273131.1 | Nakaseomyces sequencing | 14/04/2011 |
| 273372 | Candida metapsilosis | 273372.1 | This work | 21/07/2014 |
| 284811 | Ashbya gossypii ATCC 10895 | ASHGO.3 | integr8 | 12/09/2010 |
| 418086 | Candida nivariensis | 418086.1 | Comparative genomics, CRG | 14/04/2011 |
| 561895 | Candida subhashii | 561895.3 | in preparation | 21/07/2014 |
| 619300 | Spathaspora passalidarum NRRL Y-27907 | 619300.1 | JGI | 05/09/2011 |
| 638633 | Pichia angusta NCYC 495 leu1.1 | 638633.1 | JGI | 05/09/2011 |
| 763406 | Pichia membranifaciens NRRL Y-2026 | 763406.1 | JGI | 05/09/2011 |
| 1136231 | Candida orthopsilosis Co 90-125 | CANO9.1 | UniProt | 03/04/2013 |
| 1382548 | Candida orthopsilosis MCO456 | 9999993.3 | Comparative genomics, CRG | 21/07/2014 |
