# Supplementary material for: The Genomic Aftermath of Hybridization in the Opportunistic Pathogen Candida metapsilosis
Source: PLoS Genet. 2015 Oct 30;11(10):e1005626. doi: 10.1371/journal.pgen.1005626 (PMC4627764; doi:10.1371/journal.pgen.1005626)
Supplement: S1 File — For each chromosome we have plotted: i) coding genes for +/- strand (grey bars) and GC-content in 1kb windows (blue plot) in the bottom track and log2 of observed vs expected value in 1kb windows for depth of coverage (blue) in the top fourteen tracks. In addition, loss of heterozygosity (LOH) regions have been marked in grey, if the same genotype as reference was kept (hapA), and orange, if alternative genotype was kept (hapB). Four replicas (pe300, pe600, mp500 and pe400ov) were analysed for PL429. C. metapsilosis genome is a mixture of heterozygous (light grey), haplotype B (dark grey) and haplotype A (orange) regions. We suspect all analysed strains originate from single hybridisation event, as most of LOH events are shared by all strains. Examples of large LOH, duplications and deletions have been annotated ie. rDNA cluster (scaffold5), scaffold5 triploidy in PL448, partial scaffold2 triploidy in SZMC21154 and PL448, and complete LOH in scaffold6 in PL448. For the sake of simplicity, only some selected duplications and deletions among those longer than 5Kb are represented (annotated as such in Supplementary S4 Table). rDNA cluster is found on the edge of the largest LOH (over 350kb, scaffold5). Interestingly, we have also found rDNA cluster in long (200kb) LOH track in C. orthopsilosis MCO448 (Pryszcz et al., 2014). (PDF) [file pgen.1005626.s021.pdf]

Supplementary file 3

Candida metapsilosis chromosomes graphs

For each chromosome we have plotted: i) coding genes for +/- strand (grey bars) and GC-content in 1kb windows (blue plot) in the bottom track and log2 of observed vs expected value in 1kb windows for depth of coverage (blue) in the top fourteen tracks. In the bottom panel, the X axis reflects the genomic position given in bp (from 0 up to 3.25Mb for the longest chromosome), while the Y axis the GC% content (0-100%). In the other panels the Y axis reflect the log2 of observed vs expected depth-of-coverage (from -4 to +4).

In addition, loss of heterozygosity (LOH) regions have been marked in grey, if the same genotype as reference was kept (hapA), and orange, if alternative genotype was kept (hapB). Four replicas (pe300, pe600, mp500 and pe400ov) were analysed for PL429.

C. metapsilosis genome is a mixture of heterozygous (light grey), haplotype B (dark grey) and haplotype A (orange) regions. We suspect all analysed strains originate from single hybridisation event, as most of LOH events are shared by all strains.

Examples of large LOH, duplications and deletions have been annotated ie. rDNA cluster (scaffold5), scaffold5 triploidy in PL448, partial scaffold2 triploidy in SZMC21154 and PL448, and complete LOH in scaffold6 in PL448. For the sake of simplicity, only some selected duplications and deletions among those longer than 5Kb are represented (annotated as such in Supplementary table S4).

rDNA cluster is found on the edge of the largest LOH (over 350kb, scaffold5). Interestingly, we have also found rDNA cluster in long (200kb) LOH track in *C. orthopsilosis* MCO448 (PMID: 24747362).

scaffold1

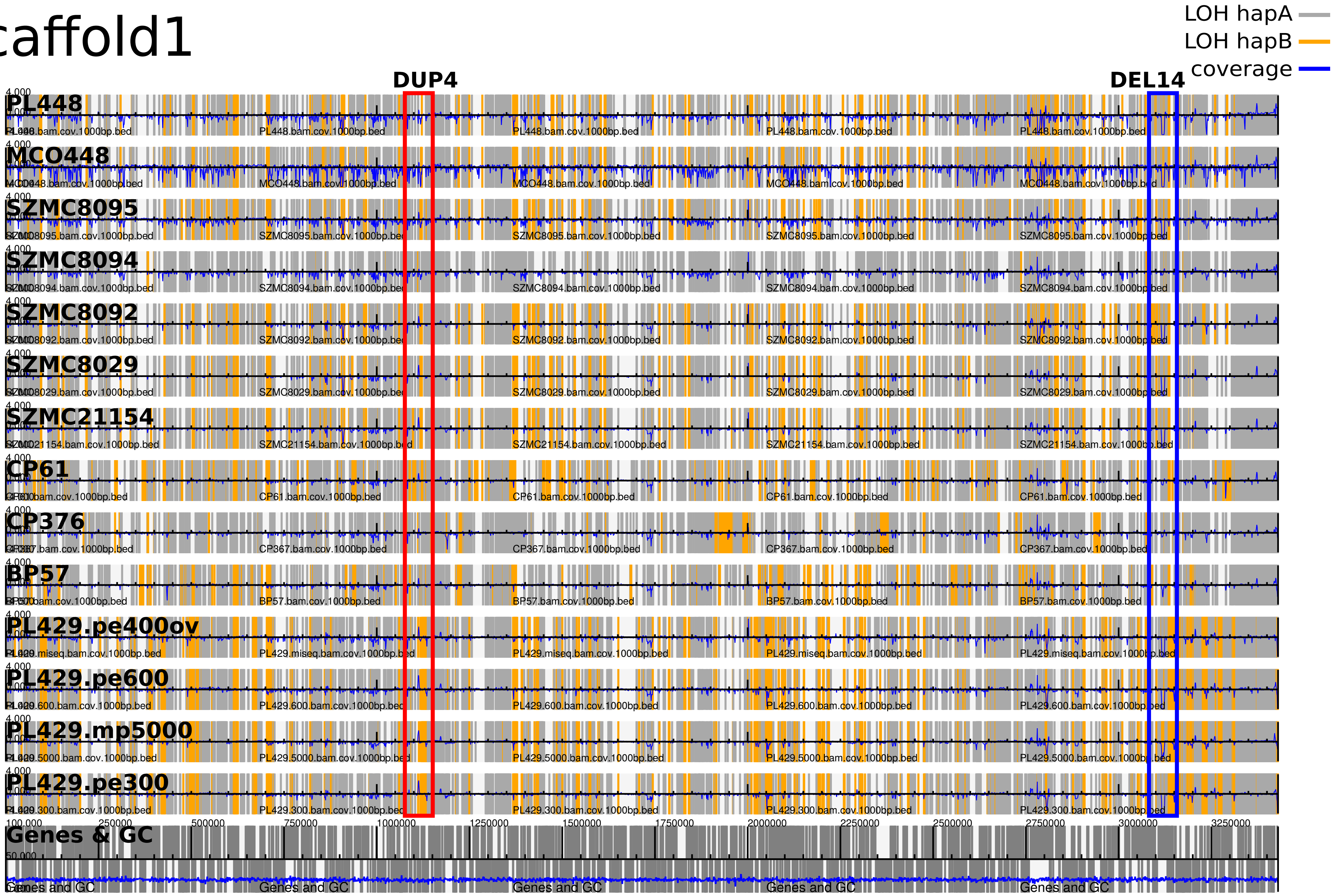

LOH hapA
LOH hapB
coverage
DUP4
DEL14
PL448
MCO448
SZMC8095
SZMC8094
SZMC8092
SZMC8029
SZMC21154
CP61
CP376
BP57
PL429.pe400ov
PL429.pe600
PL429.mp5000
PL429.pe300
Genes & GC
PL448.bam.cov.1000bp.bed
MCO448.bam.cov.1000bp.bed
SZMC8095.bam.cov.1000bp.bed
SZMC8094.bam.cov.1000bp.bed
SZMC8092.bam.cov.1000bp.bed
SZMC8029.bam.cov.1000bp.bed
SZMC21154.bam.cov.1000bp.bed
CP61.bam.cov.1000bp.bed
CP367.bam.cov.1000bp.bed
BP57.bam.cov.1000bp.bed
PL429.miseq.bam.cov.1000bp.bed
PL429.600.bam.cov.1000bp.bed
PL429.5000.bam.cov.1000bp.bed
PL429.300.bam.cov.1000bp.bed
Genes and GC
4.000
50.000
100.000
250000
500000
750000
1000000
1250000
1500000
1750000
2000000
2250000
2500000
2750000
3000000
3250000

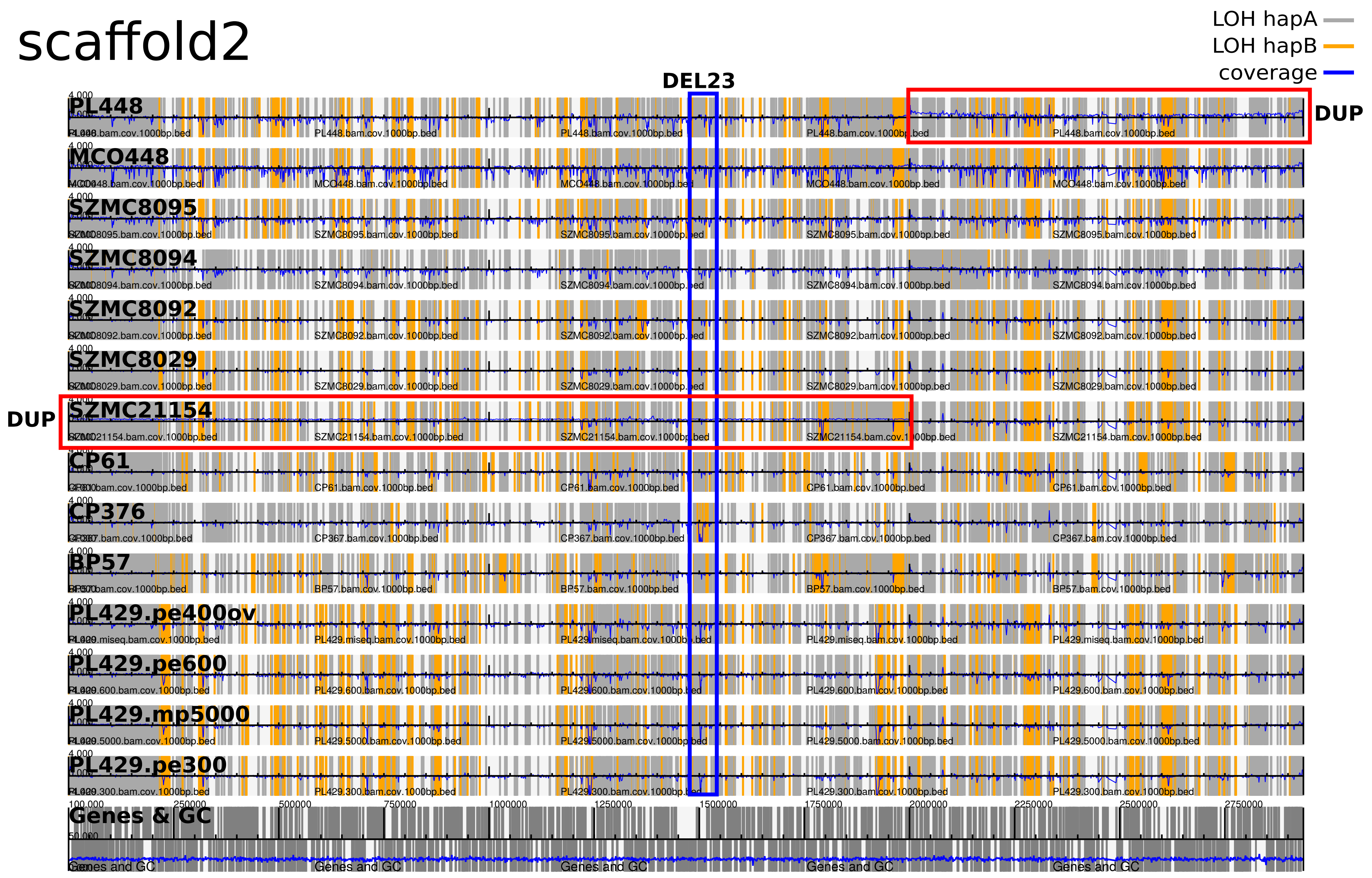
scaffold2
LOH hapA
LOH hapB
coverage
DEL23
DUP
DUP
PL448
MCO448
SZMC8095
SZMC8094
SZMC8092
SZMC8029
SZMC21154
CP61
CP376
BP57
PL429.pe400ov
PL429.pe600
PL429.mp5000
PL429.pe300
Genes & GC
100 000
250000
500000
750000
1000000
1250000
1500000
1750000
2000000
2250000
2500000
2750000
Genes and GC

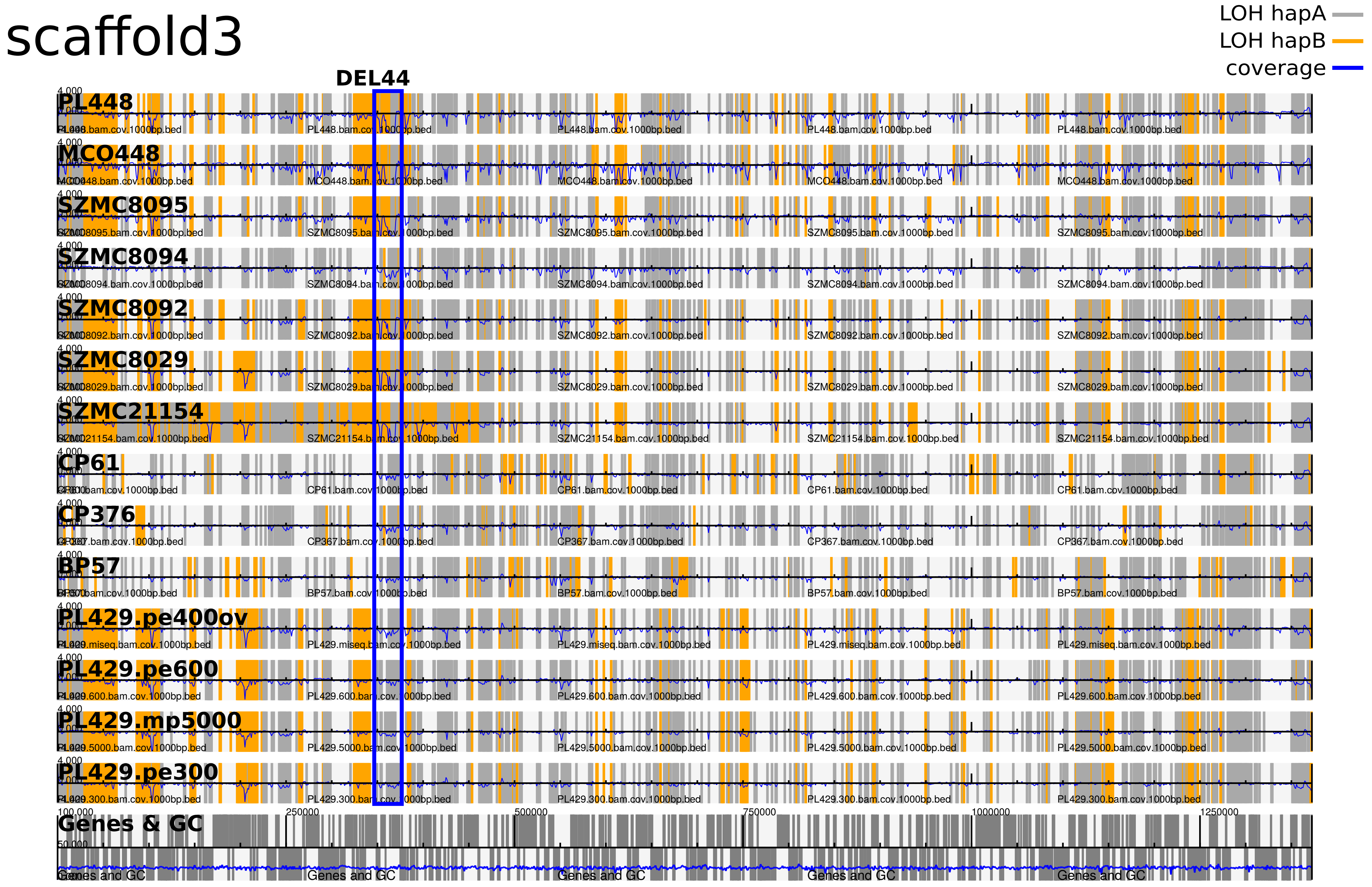
scaffold3
LOH hapA
LOH hapB
coverage
DEL44
PL448
MCO448
SZMC8095
SZMC8094
SZMC8092
SZMC8029
SZMC21154
CP61
CP376
BP57
PL429.pe400ov
PL429.pe600
PL429.mp5000
PL429.pe300
Genes & GC
100 000
250000
500000
750000
1000000
1250000
Genes and GC

scaffold4

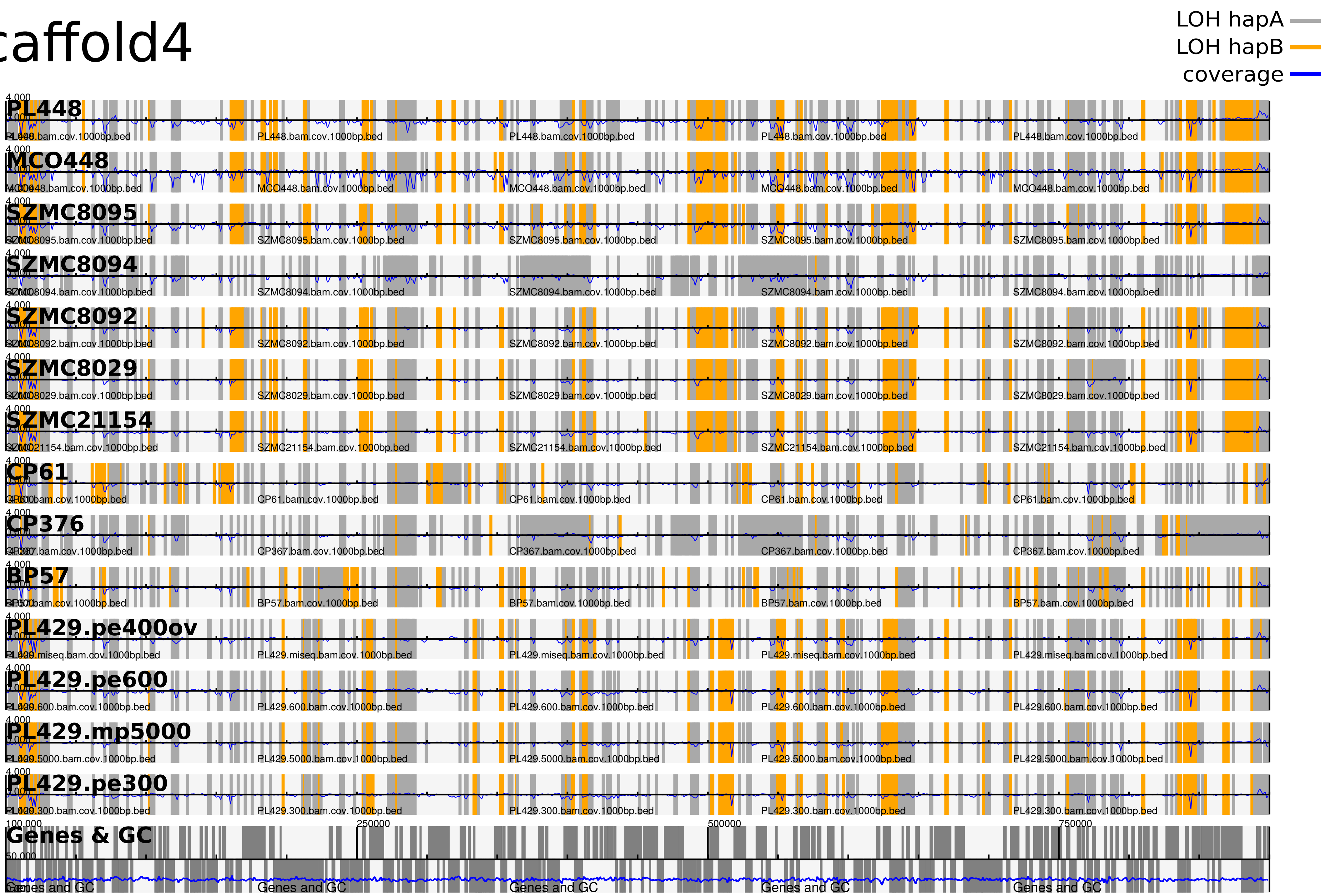

LOH hapA
LOH hapB
coverage
PL448
MCO448
SZMC8095
SZMC8094
SZMC8092
SZMC8029
SZMC21154
CP61
CP376
BP57
PL429.pe400ov
PL429.pe600
PL429.mp5000
PL429.pe300
Genes & GC
100,000
250000
500000
750000

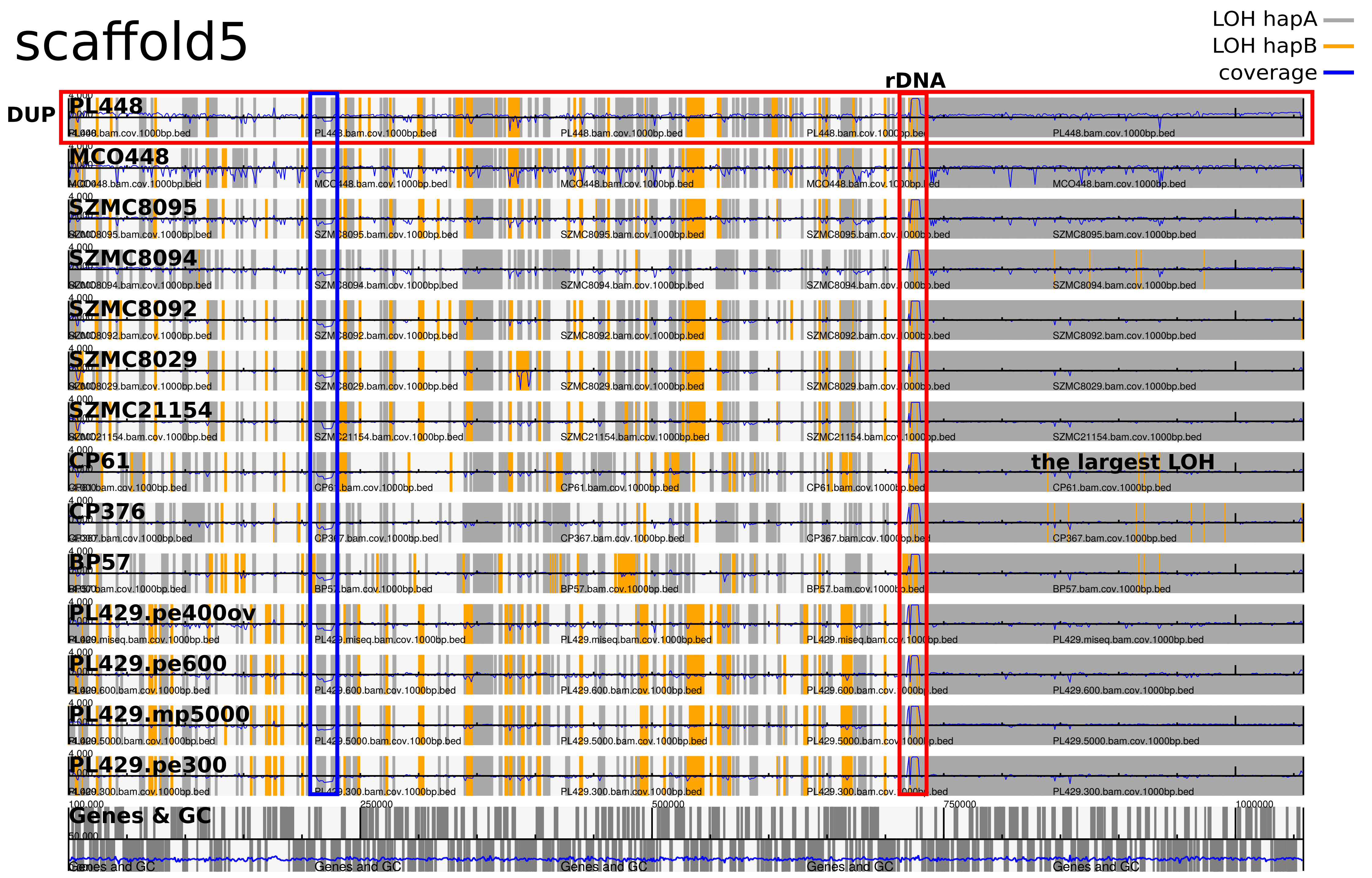
scaffold5
LOH hapA
LOH hapB
coverage
rDNA
DUP
PL448
PL448.bam.cov.1000bp.bed
MCO448
MCO448.bam.cov.1000bp.bed
SZMC8095
SZMC8095.bam.cov.1000bp.bed
SZMC8094
SZMC8094.bam.cov.1000bp.bed
SZMC8092
SZMC8092.bam.cov.1000bp.bed
SZMC8029
SZMC8029.bam.cov.1000bp.bed
SZMC21154
SZMC21154.bam.cov.1000bp.bed
CP61
CP61.bam.cov.1000bp.bed
the largest LOH
CP376
CP367.bam.cov.1000bp.bed
BP57
BP57.bam.cov.1000bp.bed
PL429.pe400ov
PL429.miseq.bam.cov.1000bp.bed
PL429.pe600
PL429.600.bam.cov.1000bp.bed
PL429.mp5000
PL429.5000.bam.cov.1000bp.bed
PL429.pe300
PL429.300.bam.cov.1000bp.bed
4.000
100.000
250000
500000
750000
1000000
Genes & GC
50.000
Genes and GC

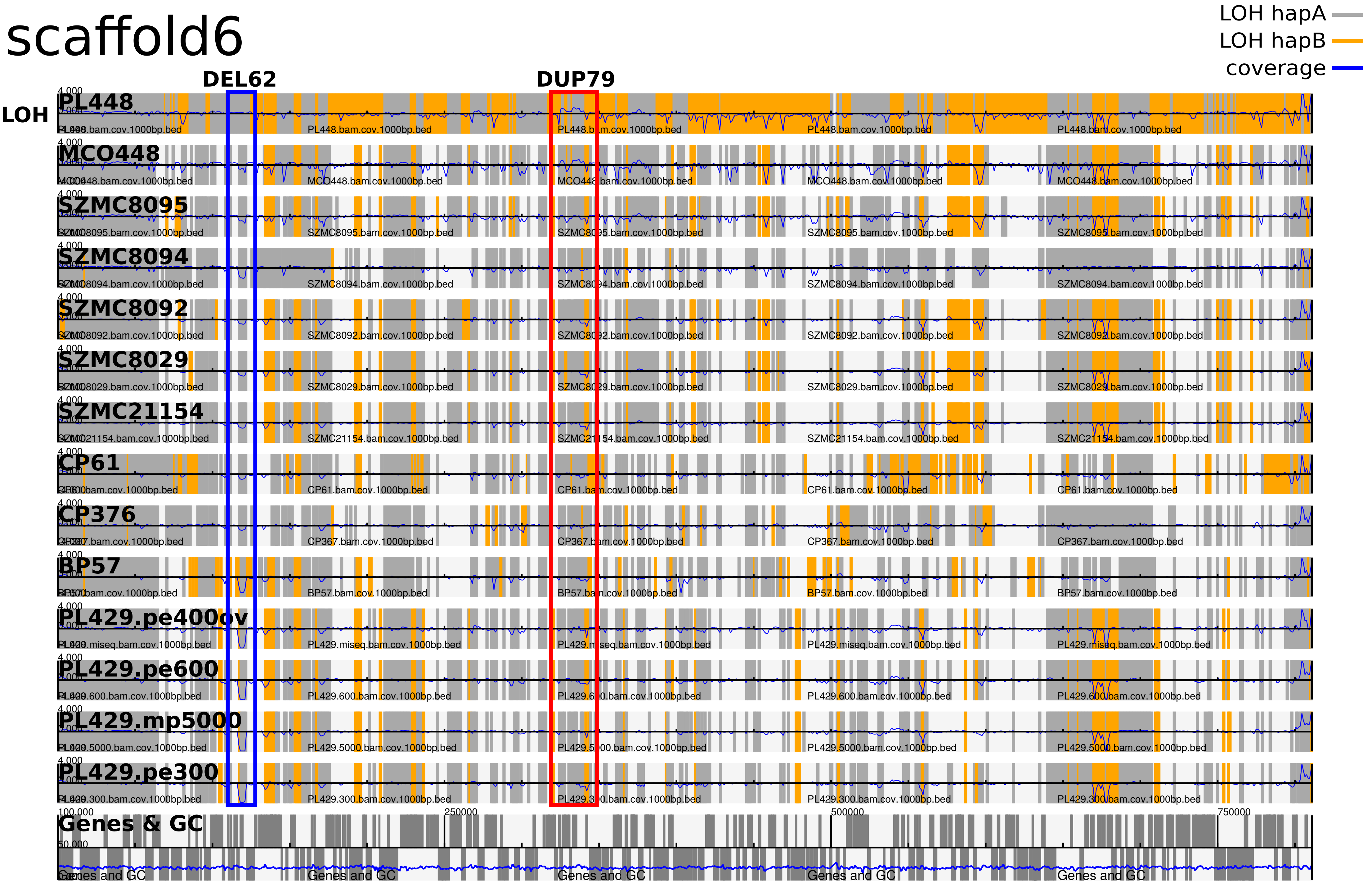
scaffold6
LOH hapA
LOH hapB
coverage
DEL62
DUP79
LOH
PL448
MCO448
SZMC8095
SZMC8094
SZMC8092
SZMC8029
SZMC21154
CP61
CP376
BP57
PL429.pe400ov
PL429.pe600
PL429.mp5000
PL429.pe300
Genes & GC
Genes and GC
250000
500000
750000

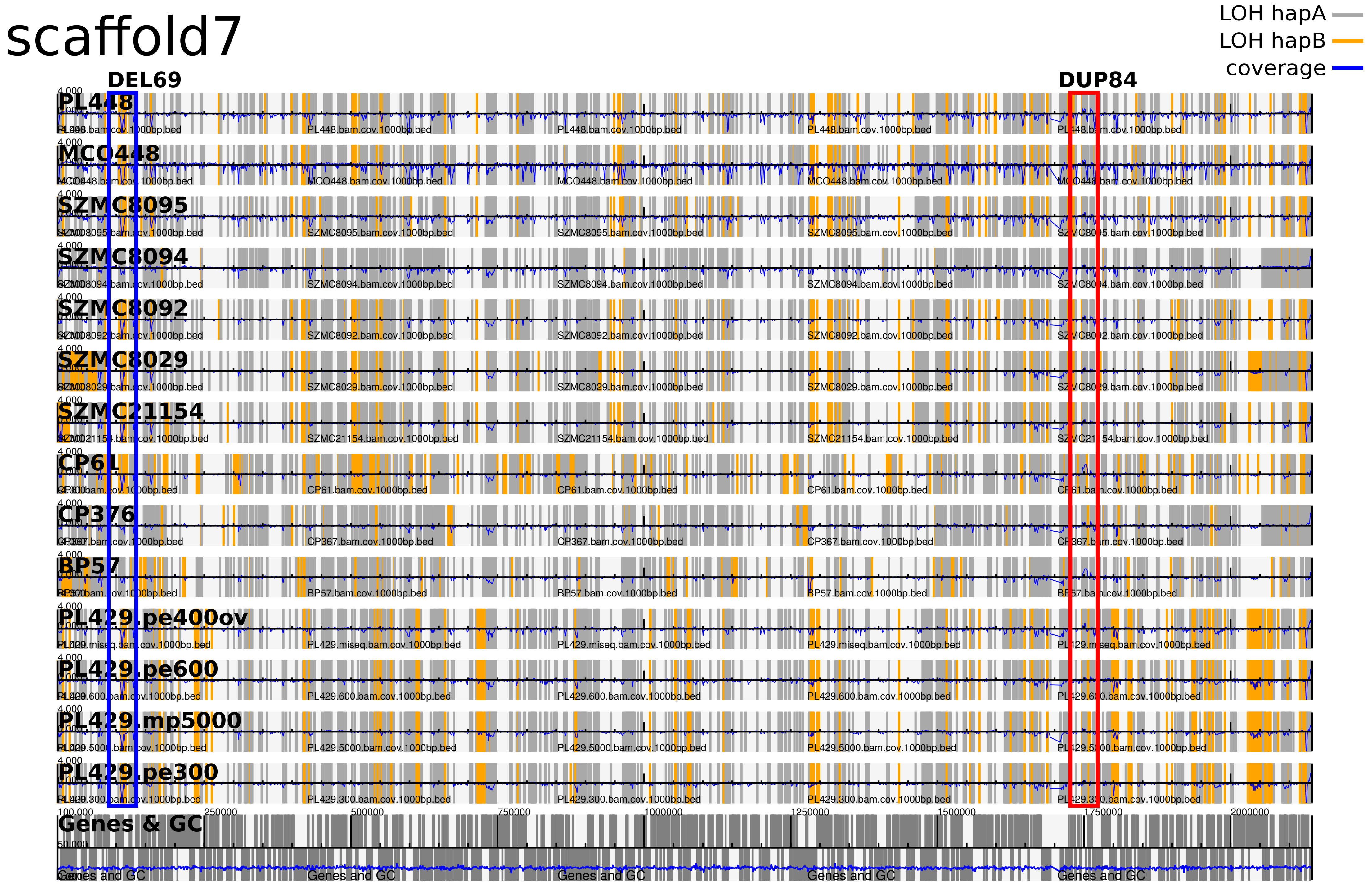
scaffold7
LOH hapA
LOH hapB
coverage
DEL69
DUP84
PL448
MCO448
SZMC8095
SZMC8094
SZMC8092
SZMC8029
SZMC21154
CP61
CP376
BP57
PL429.pe400ov
PL429.pe600
PL429.mp5000
PL429.pe300
Genes & GC
PL448.bam.cov.1000bp.bed
MCO448.bam.cov.1000bp.bed
SZMC8095.bam.cov.1000bp.bed
SZMC8094.bam.cov.1000bp.bed
SZMC8092.bam.cov.1000bp.bed
SZMC8029.bam.cov.1000bp.bed
SZMC21154.bam.cov.1000bp.bed
CP61.bam.cov.1000bp.bed
CP367.bam.cov.1000bp.bed
BP57.bam.cov.1000bp.bed
PL429.miseq.bam.cov.1000bp.bed
PL429.600.bam.cov.1000bp.bed
PL429.5000.bam.cov.1000bp.bed
PL429.300.bam.cov.1000bp.bed
Genes and GC
4.000
100.000
250000
500000
750000
1000000
1250000
1500000
1750000
2000000
50.000

scaffold8

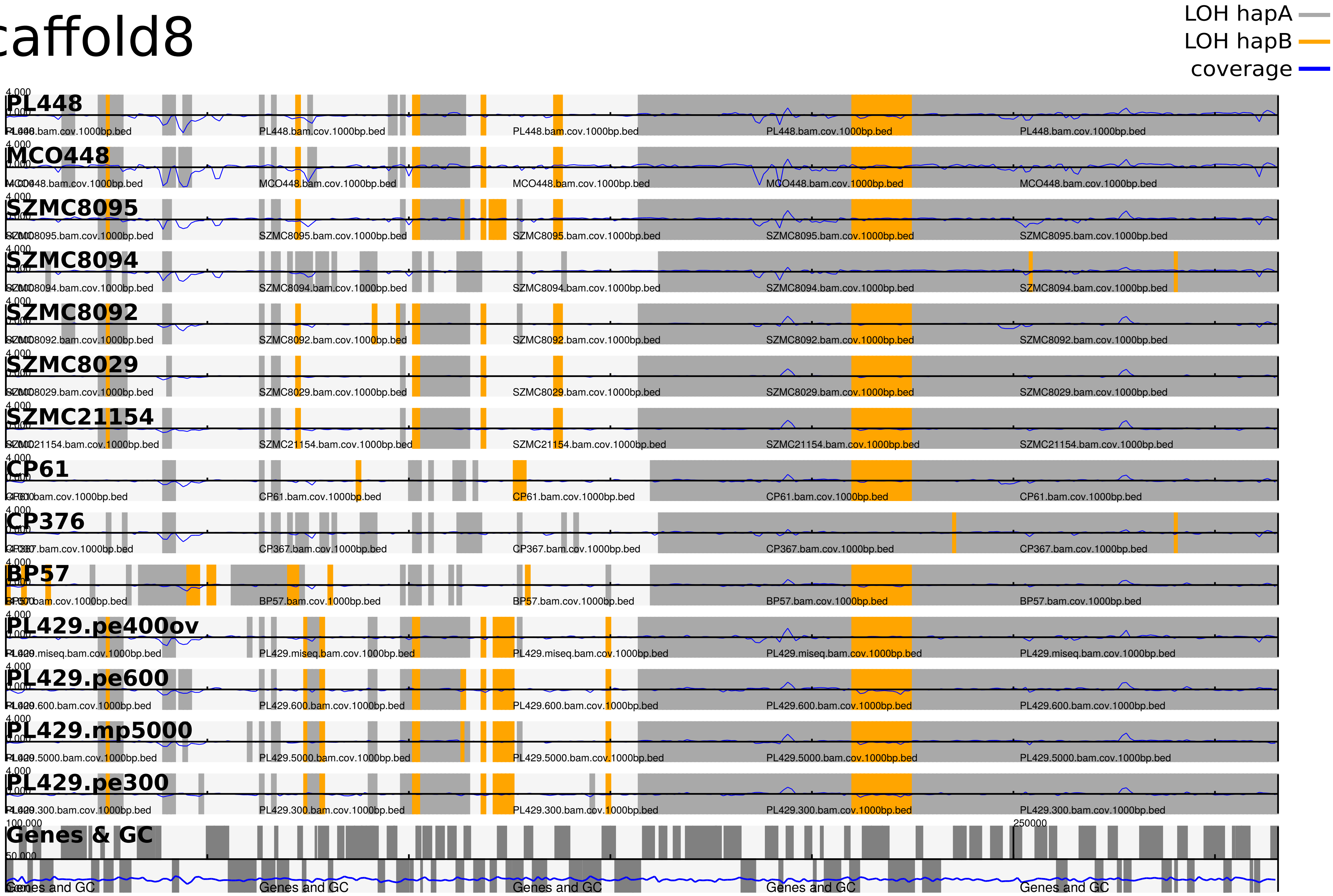

LOH hapA
LOH hapB
coverage
PL448
MCO448
SZMC8095
SZMC8094
SZMC8092
SZMC8029
SZMC21154
CP61
CP376
BP57
PL429.pe400ov
PL429.pe600
PL429.mp5000
PL429.pe300
Genes & GC
PL448.bam.cov.1000bp.bed
MCO448.bam.cov.1000bp.bed
SZMC8095.bam.cov.1000bp.bed
SZMC8094.bam.cov.1000bp.bed
SZMC8092.bam.cov.1000bp.bed
SZMC8029.bam.cov.1000bp.bed
SZMC21154.bam.cov.1000bp.bed
CP61.bam.cov.1000bp.bed
CP367.bam.cov.1000bp.bed
BP57.bam.cov.1000bp.bed
PL429.miseq.bam.cov.1000bp.bed
PL429.600.bam.cov.1000bp.bed
PL429.5000.bam.cov.1000bp.bed
PL429.300.bam.cov.1000bp.bed
Genes and GC
4.000
0.000
100.000
50.000
250000

scaffold9

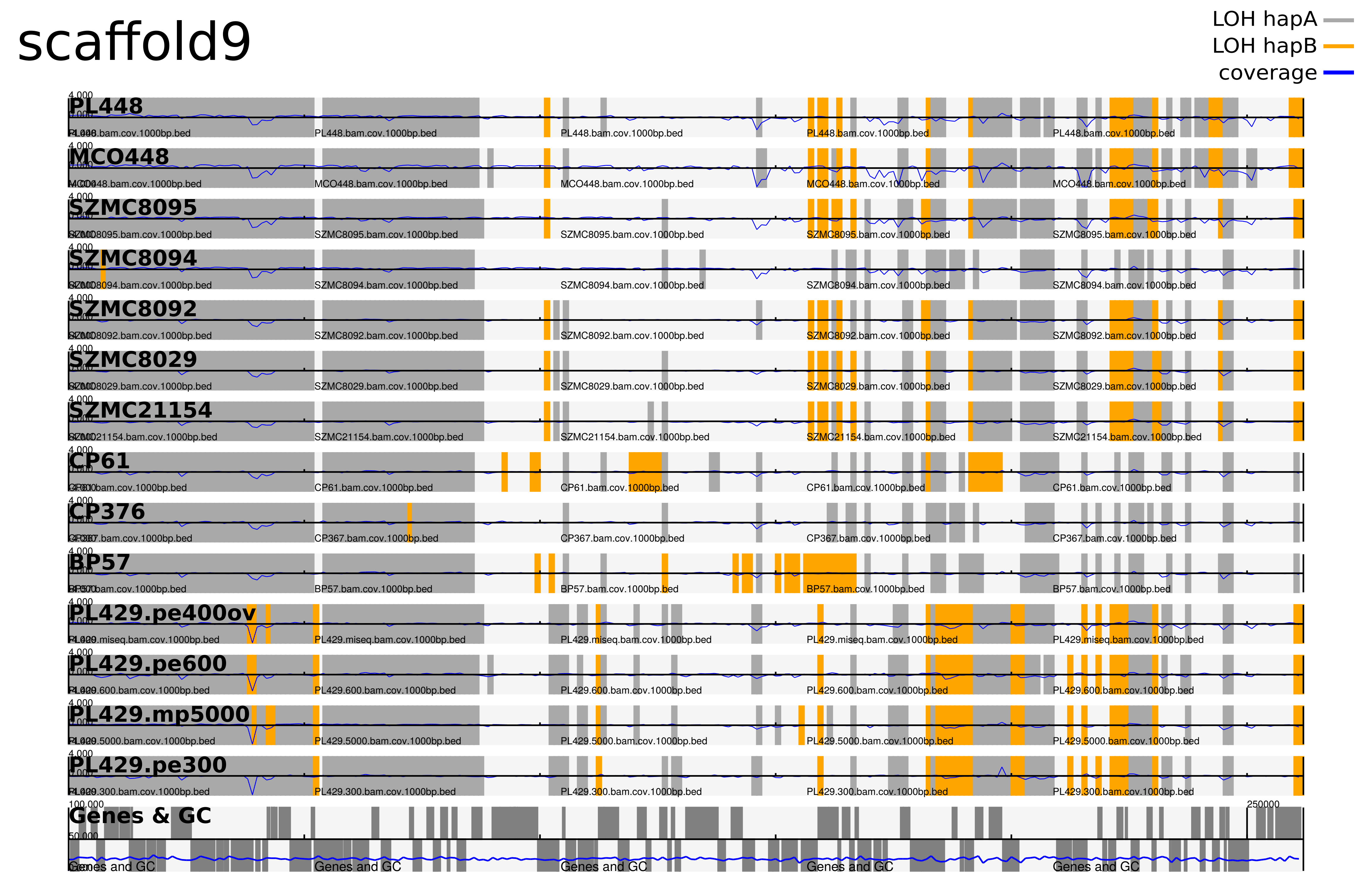
LOH hapA
LOH hapB
coverage
PL448
MCO448
SZMC8095
SZMC8094
SZMC8092
SZMC8029
SZMC21154
CP61
CP376
BP57
PL429.pe400ov
PL429.pe600
PL429.mp5000
PL429.pe300
Genes & GC
4.000
0.000
100.000
50.000
250000
PL448.bam.cov.1000bp.bed
MCO448.bam.cov.1000bp.bed
SZMC8095.bam.cov.1000bp.bed
SZMC8094.bam.cov.1000bp.bed
SZMC8092.bam.cov.1000bp.bed
SZMC8029.bam.cov.1000bp.bed
SZMC21154.bam.cov.1000bp.bed
CP61.bam.cov.1000bp.bed
CP367.bam.cov.1000bp.bed
BP57.bam.cov.1000bp.bed
PL429.miseq.bam.cov.1000bp.bed
PL429.600.bam.cov.1000bp.bed
PL429.5000.bam.cov.1000bp.bed
PL429.300.bam.cov.1000bp.bed
Genes and GC
