# Supplementary material for: The Genomic Aftermath of Hybridization in the Opportunistic Pathogen Candida metapsilosis
Source: PLoS Genet. 2015 Oct 30;11(10):e1005626. doi: 10.1371/journal.pgen.1005626 (PMC4627764; doi:10.1371/journal.pgen.1005626)
Supplement: S1 Text — (DOC) [file pgen.1005626.s022.doc]

# References

1. Boetzer, M., Henkel, C. V., Jansen, H. J., Butler, D., & Pirovano, W. (2011). Scaffolding pre-assembled contigs using SSPACE. Bioinformatics, 27, 578–579. doi:10.1093/bioinformatics/btq683
2. Gerhold, J. M., Sedman, T., Visacka, K., Slezakova, J., Tomaska, L., Nosek, J., & Sedman, J. (2014). Replication intermediates of the linear mitochondrial DNA of Candida parapsilosis suggest a common recombination based mechanism for yeast mitochondria. Journal of Biological Chemistry, 289(33), 22659–22670. doi:10.1074/jbc.M114.552828
3. Huang, S., Chen, Z., Huang, G., Yu, T., Yang, P., Li, J., … Xu, A. (2012). HaploMerger: reconstructing allelic relationships for polymorphic diploid genome assemblies. Genome Research, 22(8), 1581–8. doi:10.1101/gr.133652.111
4. Jung, P. P., Friedrich, A., Souciet, J.-L., Louis, V., Potier, S., de Montigny, J., & Schacherer, J. (2010). Complete mitochondrial genome sequence of the yeast Pichia farinosa and comparative analysis of closely related species. Current Genetics, 56(6), 507–15. doi:10.1007/s00294-010-0318-y
5. Kurtz, S., Phillippy, A., Delcher, A. L., Smoot, M., Shumway, M., Antonescu, C., & Salzberg, S. L. (2004). Versatile and open software for comparing large genomes. Genome Biology, 5, R12. doi:10.1186/gb-2004-5-2-r12
6. Letunic, I., & Bork, P. (2011). Interactive Tree Of Life v2: online annotation and display of phylogenetic trees made easy. Nucleic Acids Research, 39(Web Server issue), W475–8. doi:10.1093/nar/gkr201
7. Luo, R., Liu, B., Xie, Y., Li, Z., Huang, W., Yuan, J., … Lam, T. (2012). SOAPdenovo2: an empirically improved memory-efficient short-read de novo assembler. GigaScience, 1(1), 18. doi:10.1186/2047-217X-1-18
8. McKenna, A., Hanna, M., Banks, E., Sivachenko, A., Cibulskis, K., Kernytsky, A., … DePristo, M. A. (2010). The Genome Analysis Toolkit: a MapReduce framework for analyzing next-generation DNA sequencing data. Genome Research, 20, 1297–1303. doi:10.1101/gr.107524.110
9. Nosek, J., Rycovska, A., Makhov, A. M., Griffith, J. D., & Tomaska, L. (2005). Amplification of telomeric arrays via rolling-circle mechanism. The Journal of Biological Chemistry, 280, 10840–10845. doi:10.1074/jbc.M409295200
10. Stamatakis, A., Ludwig, T., & Meier, H. (2005). RAxML-III: A fast program for maximum likelihood-based inference of large phylogenetic trees. Bioinformatics, 21, 456–463. doi:10.1093/bioinformatics/bti191
11. Wehe, A., Bansal, M. S., Burleigh, J. G., & Eulenstein, O. (2008). DupTree: a program for large-scale phylogenetic analyses using gene tree parsimony. Bioinformatics (Oxford, England), 24(13), 1540–1. doi:10.1093/bioinformatics/btn230
